# Prenylated Isoflavanones with Antimicrobial Potential from the Root Bark of *Dalbergia melanoxylon*

**DOI:** 10.3390/metabo13060678

**Published:** 2023-05-23

**Authors:** Duncan Mutiso Chalo, Katrin Franke, Vaderament-A. Nchiozem-Ngnitedem, Esezah Kakudidi, Hannington Origa-Oryem, Jane Namukobe, Florian Kloss, Abiy Yenesew, Ludger A. Wessjohann

**Affiliations:** 1 Department of Plant Sciences, Microbiology and Biotechnology, Makerere University, Kampala P.O. Box 7062, Uganda; dunmutiso6@gmail.com (D.M.C.); esezahk@gmail.com (E.K.); horyemoriga@gmail.com (H.O.-O.); 2 Leibniz Institute of Plant Biochemistry, Weinberg 3, 06120 Halle (Saale), Germany; 3 Department of Biology, University of Nairobi, Nairobi P.O. Box 30197-0100, Kenya; 4 Institute of Biology/Geobotany and Botanical Garden, Martin Luther University Halle-Wittenberg, 06108 Halle (Saale), Germany; 5 German Centre for Integrative Biodiversity Research (iDiv) Halle-Jena-Leipzig, 04103 Leipzig, Germany; 6 Department of Chemistry, University of Nairobi, Nairobi P.O. Box 30197-0100, Kenya; n.vaderamentalexe@gmail.com; 7 Department of Chemistry, Makerere University, Kampala P.O. Box 7062, Uganda; jnamukobe@gmail.com; 8 Transfer Group Anti-Infectives, Leibniz Institute for Natural Product Research and Infection Biology, Leibniz-HKI, Beutenbergstr. 11a, 07745 Jena, Germany; florian.kloss@leibniz-hki.de

**Keywords:** *Dalbergia melanoxylon*, Fabaceae, prenylated isoflavanones, antibacterial, antifungal, anti-helminthic, cytotoxic activities

## Abstract

*Dalbergia melanoxylon* Guill. & Perr (Fabaceae) is widely utilized in the traditional medicine of East Africa, showing effects against a variety of ailments including microbial infections. Phytochemical investigation of the root bark led to the isolation of six previously undescribed prenylated isoflavanones together with eight known secondary metabolites comprising isoflavanoids, neoflavones and an alkyl hydroxylcinnamate. Structures were elucidated based on HR-ESI-MS, 1- and 2-D NMR and ECD spectra. The crude extract and the isolated compounds of *D. melanoxylon* were tested for their antibacterial, antifungal, anthelmintic and cytotoxic properties, applying established model organisms non-pathogenic to humans. The crude extract exhibited significant antibacterial activity against Gram-positive *Bacillus subtilis* (97% inhibition at 50 μg/mL) and antifungal activity against the phytopathogens *Phytophthora infestans*, *Botrytis cinerea* and *Septoria tritici* (96, 89 and 73% at 125 μg/mL, respectively). Among the pure compounds tested, kenusanone H and (3*R*)-tomentosanol B exhibited, in a panel of partially human pathogenic bacteria and fungi, promising antibacterial activity against Gram-positive bacteria including methicillin-resistant *Staphylococcus aureus* (MRSA) and *Mycobacterium* showing MIC values between 0.8 and 6.2 μg/mL. The observed biological effects support the traditional use of *D. melanoxylon* and warrant detailed investigations of its prenylated isoflavanones as antibacterial lead compounds.

## 1. Introduction

The genus *Dalbergia* L.f. (Fabaceae) consists of approximately 274 species distributed in the tropics and subtropics. Among these, eight species naturally occur in Kenya [1,2]. Plants of this genus vary from shrubs and lianas to small trees referred to as rosewoods (e.g., *D. odorifera* T.C. Chen., *D. latifolia* Roxb. and *D. melanoxylon* Guill. & Perr.) due to their fine timber of high economic value [3]. The genus is extensively used in the treatment of various ailments in traditional medicine globally [2,4]. Furthermore, pharmacological activities such as anti-inflammatory, antiallergic [5], antibacterial, antifungal [6], antiplasmodial [7], larvicidal and mosquito repellant [8], antidiarrheal [9], anthelmintic [10], anticancer [11], antidiabetic [12] and antigiardial [13] activities have been reported. Consequently, the genus *Dalbergia* has attracted wide phytochemical interest and exhibits a broad spectrum of biologically active secondary metabolites such as flavonoids, isoflavonoids, neoflavones, sterols, quinones, cinnamyl phenols and triterpenes [2,14]. Flavonoids are quite abundant in nature while prenylated flavonoids are much less common and their distribution is mostly restricted to the family Fabaceae, but to a lesser extent are reported from Cannabaceae, Guttiferae, Hypericaceae, Moraceae, Rutaceae and Umbelliferae [15]. Rare 3-hydroxisoflavanones [16] and flavonoids having isoprene or geranyl units attached to B- and C-rings have been described in the genus *Dalbergia* and also occur in a few other related genera such as *Sophora* and *Echinosophora* [17,18]. Prenylation in flavonoids increases lipophilicity and thus affinity to biological membranes. Interaction with target proteins is improved and therefore antibacterial, antifungal, anti-inflammatory, antioxidant and cytotoxic activities are enhanced compared to the parent compound [19,20,21]. Moreover, isoflavonoids have continued to gain tremendous attention due to their phytoestrogenic effects leading to a correlation between their dietary consumption and health-advantageous effects towards osteoporosis, hormone-related cancer, cardiovascular diseases and menopausal symptoms. The dietary intake of legumes represents the main source of these so-called phytoestrogens which play a pivotal role for both humans and animals [22].

*Dalbergia melanoxylon* Guill. & Perr. (known as African Blackwood) occurs as a shrub or small tree with a wide ecological versatility in semi-arid, sub-humid and tropical lowland areas in Eastern Africa [1]. Traditionally, African Blackwood is widely applied in African communities. For instance, in Kenya, the leaves are boiled with goat soup to relieve joint pains [23], while the bark decoction is used in Zimbabwe for cleaning wounds [6]. Furthermore, *D. melanoxylon* is used indigenously in South Africa and Zambia as an emetic and aphrodisiac, respectively [6]. The ethnobotanical utilization of the roots in management of abdominal pain, helminths, gonorrhea, stomachache and as a mouth wash for toothache is documented in Kenya and Zimbabwe [6,24]. Crude extracts from different plant parts of *D. melanoxylon* have shown significant antibacterial and antifungal activity [6,25]. Besides the traditional and pharmacological applications, African blackwood has been used for decades for the manufacturing of musical instruments (oboe and clarinet) and ornamental objects (carvings, tables, sofas) ranking it among the most expensive timbers in the world [26]. Phytochemical investigations on the stem bark of this species led to the isolation of dihydrobenzofurans (melanoxin), neoflavones (*S*)(+)-3′-hydroxyl-4′,2,4,5-tetramethoxy-dalbergiquinol), a quinone (4-hydroxyl-4-methoxydalbergione) and isoflavanones (kenusanone F 7-methyl and sophoronol-7-methyl ether) [16,27,28,29]. A recent study also demonstrated the cardioprotective effects of several neoflavonoids from the heartwood of *D. melanoxylon* [30].

Because various tissues including the roots of *D. melanoxylon* are traditionally used to treat infection-related conditions, we hypothesized that phytochemical investigation of root bark might yield new secondary metabolites with antimicrobial properties. Although the plant species has attracted considerable attention from the scientific community, no phytochemical investigation or biological evaluation of *D. melanoxylon* root bark has been undertaken to date. The present study aims to fill this gap. Thus, the root bark of the target plant was investigated phytochemically leading to the isolation and identification of fourteen compounds, of which six are described for the first time. Herein, the isolation, structure elucidation, antibacterial, antifungal, anthelminthic and cytotoxic activity of these compounds are discussed.

## 2. Materials and Methods

### 2.1. General Experimental Procedures

Column chromatography was performed on silica gel (0.040–0.063 μm) (Merck, Darmstadt, Germany). Analytical TLC was conducted on silica gel plates 60 F254 (Merck). Spots were visualized using UV light at 254 and 366 nm or by spraying with vanillin-H_2_SO_4_. ^1^H, ^13^C NMR and 2D spectra were recorded on an Agilent DD2 400 NMR spectrometer at 399.915 and 100.569 MHz, a Bruker Avance NEO 500 NMR spectrometer equipped with a TXO cryogenic probe operating at 500 and 125 MHz and on an Agilent VNMRS 600 NMR spectrometer at 600 and 150 MHz, respectively. If not stated otherwise, the ^1^H NMR chemical shifts are referenced to internal TMS (δ_H_ 0.00); ^13^C NMR chemical shifts are referenced to internal methanol-d_4_ (δ_C_ 49.0). The low-resolution ESI-MS spectra were obtained from a Sciex API-3200 instrument (Applied Biosystems, Concord, Ontario, Canada) combined with an HTC-XT autosampler (CTC Analytics, Zwingen, Switzerland). HRESIMS were obtained using Orbitrap Elite Mass spectrometer (Thermofisher Scientific, Bremen, Germany) and QTOF mass spectrometer Sciex TripleTOF 6600 LC-MS System (AB Sciex, Darmstadt, Germany). UV spectra were obtained on a JASCO V-560 UV/VIS spectrophotometer (JASCO Deutschland GmbH, Pfungstadt, Germany). Optical rotations were measured using a JASCO P-2000 digital polarimeter in methanol.

### 2.2. Plant Material 

The root bark of *D. melanoxylon* Guill. & Perr. (Fabaceae) was collected from Muthetheni, Machakos County (S 1°28′60″, E 37°30′02″, El. 1200 masl) in Kenya, in December 2018. A voucher specimen (DMC/2018/001) was deposited at the University Herbarium (NAI), Department of Biology, University of Nairobi, Kenya. The species was identified by the curator of the herbarium, Mr. Patrick Mutiso. 

### 2.3. Extraction and Isolation

The ground root bark of *D. melanoxylon* (1.6 kg) was macerated in a 1:1 mixture of CH_2_Cl_2_ and MeOH to yield a gummy extract (95.7 g). The crude extract was partitioned between CH_2_Cl_2_ and H_2_O. After removal of the organic solvent 70.6 g CH_2_Cl_2_ extract was obtained. A portion of this extract (51.3 g) was subjected to column chromatography on silica gel (600 g, 80 × 4 cm) eluting with *n*-hexane containing increasing amounts of EtOAc. 

A brownish precipitate obtained from the fraction eluted with 2% EtOAc in *n*-hexane was filtered and washed with methanol to afford a mixture of cinnamic acid esters with different chain lengths dominated by 3′,4′-dihydroxyl-trans-cinnamic acid octacosyl ester (**14**, 10.3 g). The precipitate obtained from the fraction eluted with 4% EtOAc in *n*-hexane was washed with methanol, giving sophoraisoflavone A (**10**, 12.7 g) as the major compound. The mother liquor was further purified by column chromatography on silica gel (eluted with 60% CH_2_Cl_2_ in *n*-hexane) to yield compound **2** (400.4 mg), compound **3** (6.3 mg), compound **4** (4.4 mg) and compound **5** (3.2 mg). The fraction eluted with 6% EtOAc in *n*-hexane, after concentration, was subjected to column chromatography on silica gel (eluted with 50% CH_2_Cl_2_ in *n*-hexane) followed by centrifugal thin-layer chromatography using a Chromatotron (CH_2_Cl_2_/MeOH 19:1) to yield methyl dalbergin (**11**, 5.1 mg), dalbergin (**12**, 4.7 mg) and mellanein (**13**, 3.5 mg). Chromatotron separation (CH_2_Cl_2_/MeOH 19:1) of the fraction eluted at 8% EtOAc in *n*-hexane resulted in the isolation of tomentosanol B (**9**, 400 mg). The fraction (7.5 g) eluted using 30% EtOAc in *n*-hexane was further purified by column chromatography applying a gradient of CH_2_Cl_2_ with increasing amount of MeOH to yield kenusanone F (**8**, 39.6 mg), kenusanone H (**7**, 202.3 mg) and compound **6** (45.4 mg). A white precipitate obtained from the fraction eluted with 60% EtOAc in *n*-hexane was filtered and washed with acetone to afford compound **1** (6.9 mg). 

The physicochemical properties and spectroscopic data of new isolates (**1**–**6**) as well as previously not-reported data for compounds **7** and **9** are summarized below:

(3*S*)-3,4′,5,7-Tetrahydroxyl-2′-methoxy-3′-(4-hydroxyl-3-methylbut-2-enyl)-isoflavanone (**1**). White amorphous solid; [α]_D_^26^ 123.4 (c 0.160, MeOH); CD (MeOH) [*θ*]_215_ – 33,894, [*θ*]_237_ + 14,806, [*θ*]_292_ + 18,208, [*θ*]_348_ + 2614; UV (MeOH)λ_max_ (log *ε*) 291 (4.05); ^1^H (referenced to methanol-d_4_ solvent signal) and ^13^C NMR data, see Table 1 and Table 2; HRESIMS *m*/*z* 401.1234 [M − H]^−^ (calcd. for C_21_H_21_O_8_, 401.1236).

(3*R*)-6-Geranyl-4′,5,7-trihydroxyl-2′-methoxy-3′-prenylisoflavanone (**2**). White paste; [α]_D_^24^ 0.77 (c 0.300, MeOH); CD (MeOH) [*θ*]_197_ – 15,079, [*θ*]_210_ +9341, [*θ*]_285_ − 3305, [*θ*]_309_ + 5596; UV (MeOH)λ_max_ (log *ε*) 294 (4.17); ^1^H and ^13^C NMR data, see Table 1 and Table 2; HRESIMS *m*/*z* 505.2582 [M − H]^−^ (calcd. for C_31_H_37_O_6_, 505.2590).

6-((2*E*,5*E*)-7-Hydroxyl-3,7-dimethyl-octa-2,5-dienyl)-4′,5,7-trihydroxyl-2′-methoxy-3′-prenylisoflavanone (**3**). White amorphous solid; ^1^H and ^13^C NMR data, see Table 1 and Table 2; HRESIMS *m*/*z* 521.2537 [M − H]^−^ (calcd. for C_31_H_37_O_6_, 521.2539).

(*E*)-6-(6-Hydroxyl-3,7-dimethylocta-2,7-dienyl)-4′,5,7-trihydroxyl-2′-methoxy-3′-prenyl-isoflavanone (**4**). White amorphous solid; ^1^H and ^13^C NMR data, see Table 1 and Table 2; HRESIMS *m*/*z* 521.2521 [M − H]^−^ (calcd. for C_31_H_37_O_6_, 521.2539).

6-Geranyl-4′,5,7-trihydroxyl-2′-methoxy-3′-(2,3-epoxy-3-methyl-butyl)-isoflavanone (**5**). Colorless residue; ^1^H and ^13^C NMR data, see Table 1 and Table 2; HRESIMS *m*/*z* 521.2530 [M − H]^−^ (calcd. for C_31_H_37_O_6_, 521.2539).

(Z)-2′,4′,5,7-Tetrahydroxyl-8-(3,7-dimethylocta-2,6-dienyl)-isoflavanone (**6**). Pale-yellow oil; ^1^H and ^13^C NMR data, see Table 1 and Table 2; HRESIMS *m*/*z* 423.1848 [M − H]^−^ (calcd. for C_25_H_27_O_6_, 423.1808).

Kenusanone H (**7**). Yellow paste; [α]_D_^25^ − 1.1 (c 0.490, MeOH); UV (MeOH) λ_max_ (log *ε*) 292 (4.32); ^13^C NMR data, see Table 2; HRESIMS *m/z* 423.1837 [M − H]^−^ (calcd. for C_25_H_27_O_6_, 423.1808).

(3*R*)-Tomentosanol B (**9**). White paste [α]_D_^25^ − 126.7 (c 0.300, MeOH)); CD (MeOH) [*θ*]_206_ – 71,898, [*θ*]_236_ –27,726, [*θ*]_297_ – 25,304, [*θ*]_332_ +4592; UV (MeOH)λ_max_ (log *ε*) 296 (4.32); ^13^C NMR data, see Table 2; HRESIMS *m/z* 453.1913 [M − H]^−^ (calcd. for C_26_H_29_O_7_, 453.1908).

### 2.4. Biological Assays

#### 2.4.1. Antibacterial Assays 

The crude extracts of *Dalbergia melanoxylon* (50 and 500 μg/mL) and its isolated compounds (at 1 and 100 μM) were tested for their antibacterial activity against the Gram-negative *Aliivibrio fischeri* (DSM507) and the Gram-positive *Bacillus subtilis* (DSM 10) as described by dos Santos et al. [31]. Chloramphenicol (100 μM) was used as positive control and induced the complete inhibition of bacterial growth.

The results (mean value ± standard deviation, *n* = 6) were given as relative values (% inhibition) in comparison to the negative control (bacterial growth, 1% DMSO, without test compound). Negative values indicate an increase of bacterial growth. Calculations were performed applying the software Excel.

#### 2.4.2. Antifungal Assays 

The assays were performed according to the monitoring methods approved by the fungicide resistance action committee (FRAC) with minor modifications [32]. The phytopathogenic ascomycetes *Botrytis cinerea* Pers. and *Septoria tritici* Desm., and the oomycete *Phytophthora infestans* (Mont.) De Bary were used as test microorganisms. The crude extract and pure compounds were tested in 96-well microtiter plate assays at 125 and 42 μg/mL with DMSO used as negative control (max. concentration 2.5%), while epoxiconazole (100% inhibition at 42 μM) and terbinafine (67% inhibition at 42 μM) served as positive control. Five to seven days after inoculation, pathogen growth was evaluated by measurement of the optical density (OD) at λ 405 nm with a TecanGENios Pro microplate reader (5 measurements per well using multiple reads in a 3 × 3 square). Each experiment was carried out in triplicate.

#### 2.4.3. Anthelmintic Assay 

The anthelmintic bioassay was performed using the model organism *Caenorhabditis elegans* that previously was shown to correlate with anthelmintic activity against parasitic trematodes as described by Thomsen et al. [33]. The Bristol N2 wild-type strain of *C. elegans* was obtained from the Caenorhabditis Genetic Center (CGC), University of Minnesota, Minneapolis, USA. The nematodes were cultured on NGM (Nematode Growth Media) Petri plates using the uracil auxotroph *E. coli* strain OP50 as food source. In this assay, the solvent DMSO (2%) and the standard anthelmintic drug ivermectin (10 μg/mL, 100% dead worms after 30 min incubation) were used as negative and positive control, respectively.

#### 2.4.4. Cytotoxicity Assay 

Briefly, for the cytotoxicity assay, the human prostate cancer cell line PC-3 and the colon adenocarcinoma cancer cell line HT-29 (both from ATCC, Manassas, VA, USA) were used. The cell handling and assay techniques were in accordance with the method described by Khan et al. [34]. The extract was tested at the concentrations of 0.05 and 50 μg/mL. Anti-proliferative and cytotoxic effects of the extract were investigated by performing colorimetric MTT (3-(4,5-dimethylthiazol-2-yl)-2,5-diphenyltetrazolium bromide) and CV (crystal violet)-based cell viability assays (Sigma-Aldrich, Taufkirchen, Germany) after 48 h treatment time, respectively. The absorbance was measured with an automated microplate reader at 540 nm with a reference wavelength of 670 nm. Digitonin (125 μM) was used as positive control, which was set for data normalization to 0% cell viability. The results are presented as a percentage of control values obtained from untreated cultures.

#### 2.4.5. Agar Diffusion Assay 

The experiment was performed as previously published [35]. Briefly, test compounds were dissolved in dimethyl sulfoxide (DMSO) at a concentration of 1 mg/mL. Ciprofloxacin and amphotericin B (both positive control) were provided at 5 μg/mL and 10 μg/mL, respectively. The following test strains were used: *B. subtilis* (JMRC:STI:10880), *S. aureus* (JMRC:ST:10760 and JMRC:ST:33793 (MRSA)), *E. faecalis* (JMRC:ST:33700 (VRE)), *E. coli* (JMRC:ST:33699), *P. aeruginosa* (JMRC:ST:33772 and JMRC:ST:337721), *M. vaccae* (JMRC:STI:10670), *P. notatum* (JMRC:STI:50164), *C. albicans* (JMRC:STI:50163) and *S. salmonicolor* (JMRC:ST:35974). 2.4.6. MIC Assay

Minimal inhibitory concentrations were determined against *Mycobacterium vaccae* (JMRC:STI:10670), MRSA (JMRC:ST:33793) and *Enterococcus faecalis* (JMRC:ST:33700 (VRE)) by serial dilutions of the DMSO test item solutions of compounds **7**, **9** and **10** (1 mg/mL) Growth was inspected visually.

#### 2.4.6. Cytotoxicity Testing (Compound **7**) 

HeLa cells (DSM ACC 57) were grown in RPMI 1640 medium supplemented with 10 mL/L ultraglutamine 1 (CAMBREX 17-605E/U1), 550 μL/L gentamicin sulfate (50 mg/mL, CAMBREX 17-518Z) and 10% heat inactivated fetal bovine serum (GIBCO Life Technologies 10270-106) at 37 °C in a 5% CO_2_ atmosphere in high density polyethylene flasks (NUNC 156340). Cells were pre-incubated for 48 h in the absence of test substances. Subsequently, HeLa cells were incubated with serial dilutions of test compounds in 96 well microplates for 72 h at 37 °C in a humidified atmosphere and 5% CO_2_. After incubation, the cytolytic effect of compounds was analyzed relative to the negative control (DMSO) using a colorimetric assay (methylene blue). The adherent HeLa cells were fixed by glutaraldehyde (MERCK 1.04239.0250) and stained with a 0.05% solution of methylene blue (SERVA 29198) for 15 min. After gentle rinsing, the stain was eluted through addition of 0.2 mL hydrochloric acid (0.33 M) to each well. The absorptions were measured at 660 nm in a SUNRISE microplate reader (TECAN). Four replicates were assayed for each substance. The half-cytotoxic concentration (CC_50_) was defined as the test compound concentration required for 50% reduction of the viable cell count in the monolayer relative to the respective untreated control. All calculations of CC_50_ values were performed with the software Magellan (TECAN).

## 3. Results and Discussion

### 3.1. Isolation and Structure Elucidation

**Figure 1:**
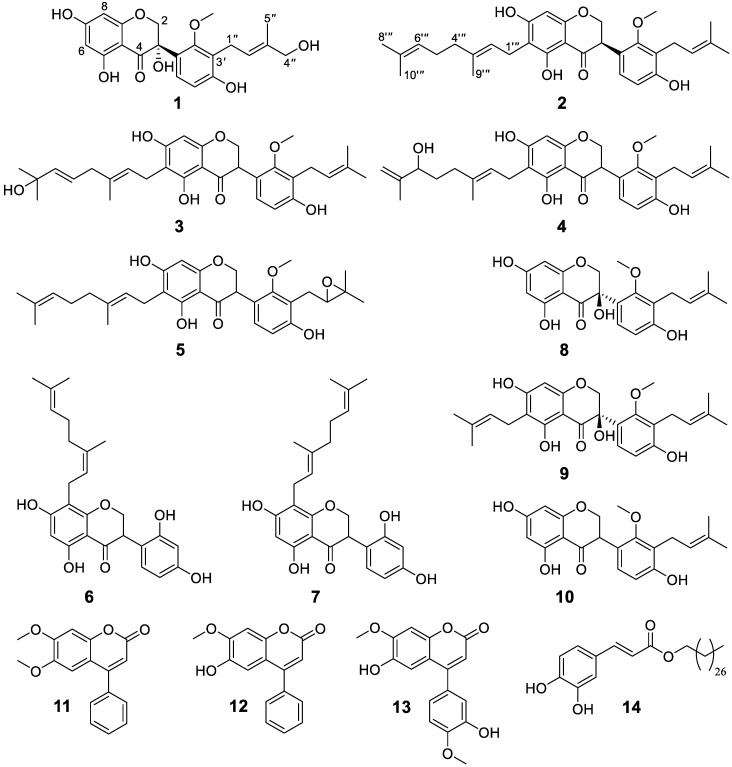
Structures of compounds **1**–**14** isolated from *Dalbergia melanoxylon*. Structures were elucidated based on HR-ESI-MS, 1- and 2-D NMR and ECD spectra and by comparison with literature values.

Chromatographic separation of the extract from the root bark of *D. melanoxylon* afforded six hitherto-undescribed isoflavanones (**1**–**6**) alongside eight known secondary metabolites comprising isoflavonoids (**7**–**10**), neoflavones (**11**–**13**) and alkyl hydroxylcinnamates (**14**) (Figure 1). Based on HRESIMS, NMR and ECD spectra and comparison to published data, the known compounds were identified as kenusanone H (**7**; [α]_D_^25^ − 1.1 (c 0.490, MeOH)), kenusanone F (**8**; [α]_D_^25^ − 112.5 (c 0.260, MeOH)) previously isolated from *Echinosophora koreensis* [36], tomentosanol B (**9**; [α]_D_^25^ − 126.7 (c 0.300, MeOH)) [18] and sophoraisoflavanone A (**10**) from *E. koreensis* [17], methyl dalbergin (**11**) from *Dalbergia sissoo* [37], dalbergin (**12**) from *D. odorifera* [38], melannein (**13**) from *D. melanoxylon* [28] and a mixture of cinnamic acid esters with the main compound being 3′,4′-dihydroxyl-trans-cinnamic acid octacosyl ester (**14**) known from *Gliricidia sepium* [39]. With the exception of compounds **12** and **13**, all known compounds were isolated for the first time from *D. melanoxylon*. For tomentosanol B (**9**) so far only the planar structure based on ^1^H NMR data was described [18]. Herein we report its ^13^C (Table 2) and 2D NMR data (Figures S9_3 and S9_4, Table S9). Based on ECD measurements (Figure S9_6) the configuration at C3 was determined as *R* and compound **9** thus elucidated as (3*R*)-6-prenyl-3,4′,5,7-trihydroxyl-2′-methoxy-3′-prenyl-isoflavanone (trivial name (3*R*)-tomentosanol B).

Compound **1** was purified as a white amorphous solid. It shows a deprotonated molecular ion in the HRESIMS at *m*/*z* 401.1234 [M − H]^−^ (calcd. for C_21_H_21_O_8_, 401.1236), corroborating the molecular formula C_21_H_22_O_8_ (degree of unsaturation: 11 double bond equivalents (DBE)). Its ^1^H [*δ*_H_ 4.70 (d, ^2^J = 11.8 Hz, H-2A), 4.03 (d, ^2^J = 11.8 Hz, H-2B)] and ^13^C [*δ*_C_ 75.8 (C-2), 75.4 (C-3), 197.1 (C-4)] NMR spectral data displayed the signature of a 3-hydroxylisoflavanone core [16,40]. In addition, the NMR also exhibited signals of a methoxy (*δ*_H_ 3.58, *δ*_C_ 62.0) and a 3-hydroxylmethyl-3-methylbut-2-enyl [*δ*_H_ 3.40, 3.33 (dd, J = 14.9, 6.4 Hz, H-1″A/1B″), 5.52 (t, J = 6.4 Hz, H-2″), 3.91 (s, H-4″), 1.76 (s, H-5″)] substituent. Furthermore, compound **1** showed the typical pattern for meta-coupled protons of a 5,7-dioxygenated A-ring [*δ*_H_ 5.93 (d, J = 2.0 Hz, H-6) and 5.88 (d, J = 2.0 Hz, H-8)] alongside two ortho-coupled doublets of an AX spin system derived from B-ring protons [*δ*_H_ 7.27 (d, J = 8.5 Hz, H-6′), 6.61 (d, J = 8.5 Hz, H-5′)]. These observations were further supported with 2D spectra which showed cross-peaks in the ^1^H-^1^H COSY spectrum between H-6 and H-8 in the A-ring, and between H-5′ and H-6′ in the B-ring. Analysis of the ^13^C-NMR spectrum indicated, in accordance with the molecular formula, the presence of 21 carbons with resonances ranging from *δ*_C_ 195.7 (sp^2^ hybridized ketone) to 12.6 (sp^3^ hybridized methyl unit). The ^13^C-NMR chemical shift of the deshielded methoxy group signal (*δ*_C_ 62.0) indicated that it is di-ortho-substituted with two bulky groups, which is consistent with its placement at C-2′ [16,41]. This finding was further confirmed with NOESY correlations observed between 2′-OMe (*δ*_H_ 3.58) and H-2A (*δ*_H_ 4.70). HMBC correlations from H-1″A (*δ*_H_ 3.40) to C-2′ (*δ*_C_ 157.7), C-3′ (*δ*_C_ 122.1), C-4′ (*δ*_C_ 158.7) and C-2″ (*δ*_C_ 125.8) indicated the placement of the isoprenyl unit at C-3′. Analysis of the NMR spectroscopic data showed its structural similarity to kenusanone F 7-methyl ether (C_22_H_24_O_7_) isolated previously from stem bark of D. melanoxylon [16] and to kenusanone F (C_21_H_22_O_7_, **8**) obtained from stem bark of Erythrina brucei [42] and also isolated in this study. The difference is that compound **1** is missing one methyl group compared to kenusanone F 7-methyl ether while it possesses one more hydroxyl group than the two other compounds. The placement of the additional OH in the prenyl chain at C-4″ (*δ*_C_ 68.9) was supported by NOESY correlation between *δ*_H_ 5.52 (H-2″) and 3.91 (H-4″).

**Figure 2:**
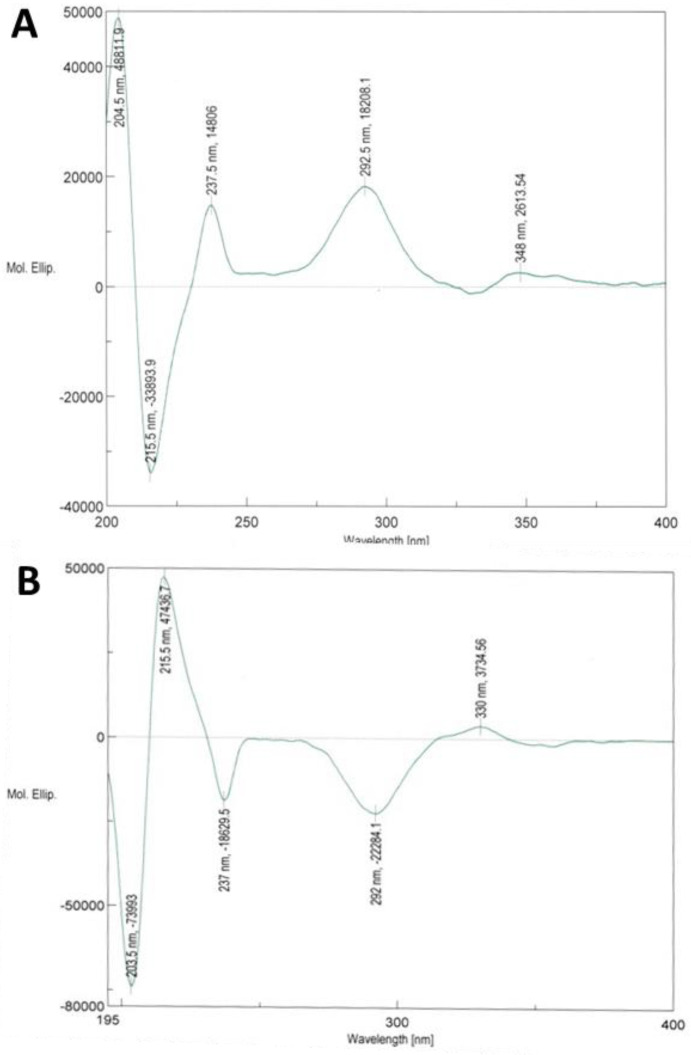
ECD spectra of (**A**) (3*S*)-3,4′,5,7-tetrahydroxyl-2′-methoxy-3′-(4-hydroxyl-3-methylbut-2-enyl)-isoflavanone (**1**) and (**B**) (3*R*)-kenusanone F (**8**).

The absolute configuration of **1** was assigned by ECD spectroscopy. Usually, the octant rule modified for cyclic arylketones is applied to determine the stereochemistry of isoflavanones [43]. This predicts a positive Cotton effect (CE) for the n → π* carbonyl transition between 320–352 nm for (3R)-isoflavanones with the B-ring in the favored equatorial position [16,43]. However, it should be kept in mind that the priority order according to the Cahn–Ingold–Prelog rules changes when hydrogen at C-3 in isoflavanones is replaced with a hydroxyl group in 3-hydroxylisoflavanones. Thus, (3R)-isoflavanones show the same spatial arrangement as (3S)-hydroxylisoflavanones. However, at least for 3-hydroxylisoflavones, the octant rule is not fully reliable and seems to be prone to misinterpretation. The ECD spectrum of **1** shows intense positive Cotton effects at 237, 292 and 348 nm, and a weak negative one around 330 nm (Figure 2). The weak CEs in the long wavelength region, around 330 (negative CE) or around 348 nm (positive CE), may not be reliable for the assignment of the absolute configuration of compound **1**. However, the ECD spectrum of **1** appears similar to the one calculated for (3*S*)-kenusanone F 7-methyl ether with a negative CE at 330 nm [42] and shows a mirror image to (3R)-kenusanone F (**8**, Figure 2; [42], hence it is consistent with (3*S*)-**1** configuration. This previously undescribed compound (**1**) was therefore characterized as (3*S*)-3,4′,5,7-tetrahydroxyl-2′-methoxy-3′-(4-hydroxylprenyl)isoflavanone.

Compound **2** was obtained as a white paste. Its molecular formula, C_31_H_38_O_6_ (13 DBE) was established by means of HRESIMS (m/z 505.2582 [M *−* H]^−^, (calcd. for C_31_H_37_O_6_, 505.2590)) and NMR data. The ^1^H [*δ*_H_ 4.40 (d, ^2^J = 8.9 Hz, H-2A), 4.39 (d, ^2^J = 6.7 Hz, H-2B), 4.26 (dd, J = 8.9, 6.7 Hz, H-3)] and ^13^C NMR [*δ*_C_ 72.5 (C-2), 47.0 (C-3) and 199.4 (C-4)] spectral data confirmed that compound **2** possesses an isoflavanone skeleton [40]. The 1D and 2D NMR data of compound **2** were similar to those of sophoraisoflavanone A (**10**), isolated from *Erythrina droogmansiana* [44] and *Sophora tomentosa* [45], except for the presence of an additional geranyl group [*δ*_H_ 3.22 (d, J = 7.2 Hz, H-1‴), 5.20 (t, J = 7.2 Hz, H-2‴), 1.95 (m, H-4‴), 2.05 (m, H-5‴), 5.05 (br t, J = 6.8 Hz, H-6‴), 1.61 (s, H-8‴), 1.74 (s, H-9‴) and 1.55 (s, H-10‴); *δ*_C_ 21.8 (C-1‴), 124.0 (C-2‴), 135.2 (C-3‴), 40.9 (C-4‴), 27.7 (C-5‴), 125.5 (C-6‴), 132.0 (C-7‴), 25.9 (C-8‴), 16.2 (C-9‴) and 17.7 (C-10‴)] at C-6 in **2**. The tail-to-head linkage of the two isoprenyl moieties to form the geranyl group was further corroborated using ^1^H-^1^H COSY correlations between H-5‴/H-6‴ and H-5‴/H-4‴. HMBC cross-peaks from H-1‴ (*δ*_H_ 3.22) to C-5 (*δ*_C_ 162.8), C-6 (*δ*_C_ 109.8), C-7 (*δ*_C_ 166.0), C-2‴ (*δ*_C_ 124.0) and C-3‴ (*δ*_C_ 135.2) clearly establish the location of the geranyl substituent at C-6. A Cotton effect for n → π* transition was not observed in compound **2**, probably due to low concentration, and hence could not be used to determine absolute configuration. On the other hand, as in compound **1**, compound **2** showed a strong positive Cotton effect for π → π* transition at 309 nm, allowing the assignment of the same absolute configuration at C-3, but the designation is R (due to change in priority because of the absence of OH at C-3 in compound **2**). Thus, compound **2** was elucidated as (3R)-6-geranyl-4′,5,7-trihydroxyl-2′-methoxy-3′-prenylisoflavanone.

Compounds **3**, **4** and **5** were assigned the same molecular formula, C_31_H_38_O_7_ (13 DBE) based on HRESIMS (m/z 521.2537 [M − H]^−^, *m*/*z* 521.2521 [M − H]^−^ and *m*/*z* 521.2582 [M − H]^−^, respectively (calcd. for C_31_H_37_O_6_, 521.2539)) combined with 1D (^1^H and ^13^C) and 2D (^1^H-^1^H COSY, HSQC and HMBC) NMR spectra. The molecular weights of **3**, **4** and **5** were 16 Dalton (Da) higher than that of **2** implying the presence of an additional oxygen atom in these compounds. Careful analyses of 1D and 2D NMR indicated that **3** and **4** had an isoflavanone skeleton similar to that of **2**, but with modifications in the geranyl residues. The terminal prenyl moiety was altered to a 3-hydroxyl-3-methyl-trans-but-1-enyl [*δ*_H_ 5.57 (m, H-5‴), 5.57 (m, H-6‴), 1.24 (s, H-8‴) and 1.24 (s, H-10‴); *δ*_C_ 126.0 (C-5‴), 139.8 (C-6‴), 71.3 (C-7‴), 29.6 (C-8‴) and 29.6 (C-10‴)] in **3** versus a 2-hydroxyl-3-methylbut-3-enyl [*δ*_H_ 1.59 (m, H-5‴), 3.95 (t, J = 6.7 Hz, H-6‴), 4.85 and 4.76 (m, H-8‴) and 1.67 (s, H-10‴); *δ*_C_ 34.1 (C-5‴), 75.9 (C-6‴), 148.4 (C-7‴), 111.2 (C-8‴) and 17.4 (C-10‴)] in **4**. The position of the hydroxyl group in compounds **3** and **4** was established using HMBC correlations from H3-8‴/H3-10‴ (*δ*_H_ 1.24) and H-6‴ (*δ*_H_ 5.57) to C-7‴ (*δ*_C_ 71.3) for **3** and from H-10‴ (*δ*_H_ 1.67) to C-6‴ (*δ*_C_ 75.9) for **4**. Hence, compounds **3** and **4** were characterized as 6-((2E,5E)-7-hydroxyl-3,7-dimethyl-octa-2,5-dienyl)-4′,5,7-trihydroxyl-2′-methoxy-3′-prenylisoflavanone (**3**) and (E)-6-(6-hydroxyl-3,7-dimethylocta-2,7-dienyl)-4′,5,7-trihydroxyl-2′-methoxy-3′-prenylisoflavanone (**4**), respectively.

Compound **5**, a colorless residue, possesses an isoflavanone scaffold as **2**–**4**, the major difference being in the isoprenyl substituent in ring B. The presence of an epoxyprenyl residue, formed through electrophilic addition of oxygen to the isoprenyl unit, was established from resonances at *δ*_H_ 3.02–2.70 (m, H-1″), 3.74 (m, H-2″), 1.32/1.34 (s, H-4″) and 1.26/1.27 (s, H-5″); *δ*_C_ 27.6 (C-1″), 70.0 (C-2″), 77.8 (C-3″), 25.6 (C-4″) and 20.8 (C-5″) as observed in **5**. Since the epoxidation seems not to be stereospecific, partly a double set of data are visible, especially for the methyl groups at position 4″ [(*δ*_H_ 1.32/1.34, s), 5″ ((*δ*_H_ 1.26/1.27, s) and 2′-OMe (*δ*_H_ 3.76/3.75, s)]. HMBC cross-peaks from H-1″ (*δ*_H_ 3.02) to C-2′ (*δ*_C_ 158.8) and C-4′ (*δ*_C_ 155.2) indicated that the epoxyprenyl moiety was located at C-3′. Hence, the planar structure of **5** was elucidated as 6-geranyl-4′,5,7-trihydroxyl-2′-methoxy-3′-(2,3-epoxy-3-methyl-butyl)-isoflavanone. Reliable optical rotation, UV and ECD spectra could not be generated for compounds **3**–**5**.

Compound **6** was obtained as pale-yellow oil. Its molecular formula was deduced as C_25_H_28_O_6_ (12 unsaturation sites) based on HRESIMS (m/z 423.1813 [M *−* H]^−^ (calcd. for C_25_H_27_O_6_, 423.1808)) in conjunction with NMR data. The ^1^H [*δ*_H_ 4.53 (t, J = 10.8 Hz, H-2A), 4.41 (dd, J = 10.8, 5.5 Hz, H-2B) and 4.17 (dd, J = 10.8, 5.5 Hz, H-3)] and ^13^C NMR [*δ*_C_ 71.4 (C-2), 47.9 (C-3) and 199.8 (C-4)] data were consistent with an isoflavanone core similar to compounds **2**–**5**. In general, the NMR data of compound **6** were superimposable to 8-geranyl-2′,4′,5,7-tetrahydroxylisoflavanone (kenusanone H, **7**) reported from the roots of Echinosophora koreensis [36] and isolated in this study. Here we report for the first time ^13^C (Table 2) and 2D NMR (Table S7) data for compound **7** which was isolated as a racemate. Nevertheless, signals observed at *δ*_C_ 32.9 (C-4‴) and 27.7 (C-5‴) in the ^13^C NMR spectrum of compound **6** indicated that, unlike kenusanone H, the C10 unit is a neryl but not a geranyl group. These findings were further supported with NOESY correlation between H-1‴/H-4‴. The HMBC signals from H-6 (*δ*_H_ 5.94) to C-5 (*δ*_C_ 163.5), C-7 (*δ*_C_ 165.8), C-10 (*δ*_C_ 103.9) and C-8 (*δ*_C_ 108.9); from H-1‴ (*δ*_H_ 3.21) to C-7 (*δ*_C_ 165.8), C-8 (*δ*_C_ 108.9), C-9 (*δ*_C_ 161.8), C-2‴ (*δ*_C_ 124.7), C-3‴ (*δ*_C_ 135.6) verified the connectivity of the neryl side chain via C-8. The substitution at C-8 is further supported by the chemical shift of the H-bonded OH at position 5 (*δ*_H_ 12.21), which is shifted downfield to 12.41–12.43 ppm in compounds **2**–**4** bearing a prenyl chain at C-6 [18,46]. Hence, compound **6** was characterized as (Z)-2′,4′,5,7-tetrahydroxyl-8-(3,7-dimethylocta-2,6-dienyl)-isoflavanone. Nerylated flavonoids are very rare in nature, and compound **6** could have been formed from the geranylated analogue **7** through isomerization.

### 3.2. Biological Activity

Since *Dalbergia* species are known to exhibit a variety of biological activities, the partitioned crude extracts and the isolated compounds of *D. melanoxylon* were tested for their antibacterial, antifungal, anthelmintic and cytotoxic properties applying an established model organism non-pathogenic to humans (Table 3). The crude CH_2_Cl_2_ extract of the root bark induced nearly complete inhibition (97% ± 0%) of the Gram-positive bacterium *Bacillus subtilis* at the concentration of 50 μg/mL and complete inhibition (100% ± 0%) of the Gram-negative bacterium Aliivibrio fischeri at 500 μg/mL showing its potential especially against Gram-positive bacteria. The antifungal and anti-oomycetes activity was evaluated against the phytopathogens *Septoria tritici, Botrytis cinerea* and *Phytophthora infestans*, respectively. The extract showed promising activity against all phytopathogens at a concentration of 125 μg/mL. No anthelminthic activity against *Caenorhabditis elegans* could be detected at 500 μg/mL. Likewise, at low concentration (0.05 μg/mL) no antiproliferative or cytotoxic effects were observed against the human cancer cell lines PC3 and HT29 whereas a higher concentration (50 μg/mL) induced significant inhibition of cell growth and viability (Table S15). These results imply that the crude extract possesses moderate cytotoxic properties but might also show selective biological effects with focus on antibacterial and antifungal activities.

Based on the results of the crude extracts, the isolated major compounds (**1**, **2**, **7**, **9**, **10**) were subjected to a preliminary biological screening in antibacterial and antifungal assays (Table 3). For the antibacterial assays, the compounds were tested at concentrations of 1 and 100 μM, and for the antifungal assays of 42 and 125 μg/mL. In both B. subtilis and A. fischeri assays, (3R)-tomentosanol B (**9**) and sophoraisoflavanone A (**10**) inhibited nearly 100% of bacterial growth at a concentration of 100 μM after 16 h incubation time. Both compounds had also a good antifungal activity against S. tritici at 125 μg/mL (corresponding to 0.28 and 0.34 mM, respectively) (Table 3). Furthermore, kenusanone H (***7***) at 42 μg/mL (0.1 mM) showed a promising growth inhibition of B. cinerea and S. tritici. Thus, these compounds were also tested against a panel of human pathogenic bacteria (Table 4) and fungi (Table S15). Kenusanone H (**7**), (3R)-tomentosanol B (**9**) and sophoraisoflavanone A (**10**) exhibited promising antibacterial activity against Gram-positive bacteria including MRSA as shown by the induction of significant inhibition zones in agar diffusion assays. Even more importantly, these compounds also inhibited the growth of Mycobacteria vaccae, a nonpathogenic member of the tuberculosis inducing the Mycobacteriaceae family. Indeed, previous docking studies indicated the potential binding of 3-hydroxylisoflavanones from *D. melanoxylon* to different mycobacterial target enzymes [16]. In the present study kensuanone H (**7**) displayed MIC values of 1.56, 1.56 and 0.78 μg/*mL* (3.7, 3.7 and 1.8 μM) against S. aureus (MRSA), *Enterococcus faecalis* and *Mycobacterium vaccae*, respectively, while tomentosanol B (**9**) inhibited the growth of these bacteria with MIC values of 3.12, 6.25 and 1.56 μg/mL corresponding to 6.9, 13.8 and 3.4 μM (Table 4), respectively. In addition, compound **7** also exhibited moderate antifungal effects against *Candida albicans, Penicillium notatum* and *Aspergillus fumigatus*, compound **9** against P*. notatum* and **10** against *S. salmicolor, C. albicans* and *P. notatum* (Table S15). Except for compounds **8**–**10**, the antimicrobial potential of the tested compounds is reported here for the first time. Nevertheless, in prior studies, kenusanone F (**8***),* purified from *E. brucei* displayed moderate activity (MIC values ranging from 125 to 250 μg/mL) against four pathogenic test organisms, namely *S. aureus*, *B. cereus*, *B. megaterium* and *E. coli* [42], while tomentosanol B (**9**) showed antiplasmodial activity (IC_50_ = 25.3 μM) and virtually no in vitro cytotoxicity against the Chinese hamster ovarian (CHO) cell line (selectivity index = 5) [47]. Sophoraisoflavanone A (**10**) isolated from *Echinosophora koreensis* was already previously described as compound with strong antifungal (*C. albicans, S. cerevisiae*) and antibacterial activity (*E. coli*, *S. typhimurium*, *S. epidermis*, *S. aureus*) showing MIC values around 60 and 20 μg/mL, respectively [19]. In addition, this compound has proven toxic (IC_50_ = 22.1 μg/mL) to a human liver (HepG2) cell line [19]. Although we could not demonstrate anthelmintic activity for the crude extract of *D. melanoxylon*, mild anthelmintic effects of prenylated isoflavonoids have been reported [48]. Neoflavonoids (represented e.g.*,* by methyl dalbergin (**11**) and dalbergin (**12**)) were not included in the biological testing in our study but were previously shown to possess osteogenic properties [37] whereas structurally related dalbergiones from *D. melanoxylon* exhibited anti-inflammatory effects [49].

Prenylated flavonoids and isoflavonoids play important roles in the defense strategy of plants by protecting them against diseases through a broad inhibition profile against bacteria and fungi [50]. At the same time, these compounds represent promising starting points for the development of new, natural therapeutics against MRSA and other Gram-positive bacteria [51]. Increased hydrophobicity and bioavailability (mediated by one or two prenyl groups) and electrostatic interactions are the main determinants for the anti-MRSA activity of prenylated isoflavonoids [51]. The effects might be mediated by damaging the membrane or cell wall function [19] whereby interaction with bacterial membranes reduces the fluidity of outer and inner membrane layers [52]. Specifically, prenylation at C-8, as present in kenusanone H (**7**), seems to be connected to strong biological activity, and also hydroxylation at C-3 in **10** plays a role for several biological effects [52]. In contrast, introduction of a hydroxyl group in the prenyl chain as in compound **1** seems to be connected to a reduction of activity. However, prenyl substitution increases antibacterial but also cytotoxic properties [52]. For the most promising candidate, kenusanone H (**7**), the cytotoxicity against HeLa cells was determined with a CC_50_ of 1.8 ± 1.4 μg/mL (4.2 μM). Prenylated flavonoids and isoflavonoids show moderate cytotoxic properties [19,50] which would have to be considered for potential applications or development.

Altogether, fourteen compounds including six new isoflavanones were isolated from the root bark of *D. melanoxylon*, a medicinal plant largely used for the treatment of infectious diseases. The crude CH_2_Cl_2_ extract of the root bark of *D. melanoxylon* induced in a concentration dependent manner different degrees of inhibition against the tested microorganisms. Among the tested compounds, **7** and **9** showed strong activities against several pathogenic microbes, while compound **10** was selective towards *M*. *vaccae* 10670 M4. It is worth noting that compounds **7** and **9** showed superior activity against *S. aureus* (MRSA) 134/93 R9 compared to the reference drug ciprofloxacin. Despite these activities, neither the crude extract, nor the tested compounds showed considerable anthelminthic and cytotoxic activities. Hence, the observed biological effects support the traditional use of *D. melanoxylon* against several conditions, which appear to be connected to bacterial or fungal infections [6,25]. The prenylated isoflavanone constituents proved to be of relevant bioactivity and are likely responsible for the activity of the roots of this plant, suggesting future investigations in terms of structure-activity-relationship, mode of action and in vivo experiments.

## Figures and Tables

**Table 1:** ^1^H NMR [*δ*_H_ (ppm), multiplicity (J in Hz)] data of compounds **1**–**6** in methanol-d_4_.

Position	1 ^a^	2 ^a^	3 ^b^	4 ^b^	5 ^b^	6 ^c^
2A	4.70, d (11.8)	4.40, d (8.9)	4.39, d (8.9)	4.40, d (6.5)	4.40, m	4.53, t (10.8)
2B	4.03, d (11.8)	4.39, d (6.7)	4.38, d (6.8)	4.39, d (9.1)	4.38, m	4.41, dd (10.8, 5.5)
3		4.26, dd (8.9, 6.7)	4.24, dd (8.9, 6.8)	4.27, dd (9.1, 6.5)	4.27, m	4.17, dd (10.8, 5.5)
6	5.93, d (2.0)					5.94, s
8	5.88, d (2.0)	5.94, s	5.94, s	5.93, s	5.94, s	
3′						6.33, d (2.4)
5′	6.61, d (8.5)	6.55, d (8.4)	6.55, d (8.4)	6.55, d (8.4)	6.53, d (8.4)	6.26, dd (8.3, 2.4)
6′	7.27, d (8.5)	6.78, d (8.4)	6.78, d (8.4)	6.77, d (8.4)	6.88, d (8.4)	6.83, d (8.3)
1″A	3.40, dd (14.9, 6.4)	3.37, dd (14.4, 6.7)	3.36, dd (14.8, 7.1)	3.34, m	3.02, m	
1″B	3.33, dd (14.9, 6.4)	3.30 ^d^	3.32 ^d^		2.70, m	
2″	5.52, t (6.4)	5.24, t (6.7)	5.24, br t (7.2)	5.24 br t (7.2)	3.74, m	
4″	3.91, s	1.68, s	1.67, s	1.67, s	1.32/1.34, s	
5″	1.76, s	1.76, s	1.77, s	1.76, s	1.26/1.27, s	
1‴		3.22, d (7.2)	3.24, d (7.3)	3.23, br d (6.5)	3.22, d (7.2)	3.21, br d (7.1)
2‴		5.20, t (7.2)	5.25, br t (7.3)	5.24, br t (6.5)	5.19, br t (6.5)	5.20, br t (7.1)
4‴		1.95, m	2.65, br d (4.5)	1.97, m	1.94, d (7.6)	2.18, m
5‴		2.05, m	5.57, m	1.59, m	2.05, m	2.08, m
6‴		5.05, br t (6.8)	5.57, m	3.95, t (6.7)	5.05, m	5.16, m
7‴						
8‴		1.61, s	1.24, s	A 4.85 m B 4.76 m	1.61, s	1.67, s
9‴		1.74, s	1.74, s	1.77, s	1.74, s	1.65, s
10‴		1.55, s	1.24, s	1.67, s	1.56, s	1.61, s
2′-OCH_3_	3.58, s	3.71, s	3.71, s	3.70, s	3.76/3.75, s	
5-OH	12.09, s	12.41, s	12.41, s	12.41, s		12.21, s

^a, b, c^ recorded at 500, 600 and 400 MHz, respectively, ^d^ overlapping with solvent signal; s: singlet; d: doublet; t: triplet; m: multiplet; br s: broadened singlet; dd: doublet of doublets.

**Table 2:** ^13^C NMR data (*δ*_C_ [ppm]) of isoflavanones **1**–**7** and **9** measured in methanol-d_4_.

Position	1 ^a^	2 ^a^	3 ^b^	4 ^b^	5 ^b^	6 ^c^	7 ^a^	9 ^a^
2	75.8	72.5	72.0 ^d^	72.2 ^d^	72.0 ^d^	71.4	71.5	75.6
3	75.4	47.0	46.8 ^d^	46.8 ^d^	46.9 ^d^	47.9	47.9	75.7
4	197.1	199.4	199.6 ^e^	199.2 ^e^	199.1 ^e^	199.8	199.8	197.3
5	166.4	162.8	162.9 ^e^	165.6 ^e^	162.8 ^e^	163.5	163.5	163.3
6	97.3	109.8	109.8 ^e^	109.4 ^e^	109.8 ^e^	96.4	96.4	109.9
7	168.3	166.0	166.1 ^e^	165.5 ^e^	166.0 ^e^	165.8	165.8	165.9
8	96.1	95.3	94.8 ^d^	95.0 ^d^	95.3 ^d^	108.9	108.9	95.4
9	164.5	162.8	162.9 ^e^	162.5 ^e^	162.8 ^e^	161.8	161.8	162.1
10	102.1	103.8	103.9 ^e^	103.5 ^e^	103.7 ^e^	103.9	103.8	102.0
1′	123.7	120.7	120.8 ^e^	120.4 ^e^	121.1 ^e^	113.8	114.1	122.7
2′	157.7	159.2	159.3 ^e^	158.8 ^e^	158.8 ^e^	157.7	157.6	157.7
3′	122.1	123.2	123.3 ^e^	122.8 ^e^	115.3 ^e^	103.8	103.8	124.5
4′	158.7	157.5	157.7 ^e^	157.2 ^e^	155.2 ^e^	159.1	159.1	158.6
5′	111.2	112.3	111.9 ^d^	112.1 ^d^	113.9^d^	107.8	107.8	111.3
6′	126.8	128.3	127.8 ^d^	128.0 ^d^	129.0 ^d^	131.9	131.9	126.6
1″	24.4	24.4	24.2 ^d^	24.1 ^d^	27.6 ^d^			4.8
2″	125.8	124.6	124.2 ^d^	124.1 ^d^	70.0 ^d^			124.0
3″	135.8	131.7	131.4 ^e^	131.4 ^e^	77.8 ^d^			132.2
4″	68.9	25.9	25.6 ^d^	25.6 ^d^	25.6 ^d^			25.9
5″	14.0	18.0	17.8 ^d^	17.7 ^d^	20.8 ^d^			17.9
1‴		21.8	21.7 ^d^	21.6 ^d^	21.5 ^d^	22.1	22.2	22.0
2‴		124.0	124.2^d^	124.3 ^d^	123.6 ^d^	124.7	124.2	123.9
3‴		135.2	134.5 ^e^	134.7 ^e^	135.2 ^e^	135.6	135.2	131.6
4‴		40.9	43.4 ^d^	36.5 ^d^	40.7 ^d^	32.9	40.8	25.8
5‴		27.7	126.0 ^d^	34.1 ^d^	27.7 ^d^	27.7	27.6	18.0
6‴		125.5	139.8 ^d^	75.9 ^d^	125.2 ^d^	125.7	125.4	
7‴		132.0	71.3 ^e^	148.4 ^e^	132.0 ^d^	131.9	132.1	
8‴		25.9	29.6 ^d^	111.2 ^d^	25.6 ^d^	25.9	25.9	
9‴		16.2	16.0 ^d^	16.0 ^d^	16.0 ^d^	23.7	16.2	
10‴		17.7	29.6 ^d^	17.4 ^d^	17.4 ^d^	17.7	17.7	
2′-OCH_3_	62.0	62.5	62.2 ^d^	62.2 ^d^	61.1 ^d^			61.9

^a, b, c^ recorded at 125, 150 and 100 MHz, respectively; ^d, e^ signals derived from HSQC and HMBC, respectively.

**Table 3:** Antibacterial (*Bacillus subtilis*, *Aliivibrio fischeri*) and antifungal (*Phytophthora infestans*, *Botrytis cinerea*, *Septoria tritici*) activities of the CH_2_Cl_2_ extract and isolated compounds from *D. melanoxylon* shown as growth inhibition [%] ^a^. Data are presented as mean values ± standard deviation (*n* = 6 for antibacterial assays, *n* = 3 for antifungal assays.

	Antibacterial Assays				Antifungal Assays					
	*B. subtilis*		*A. fischeri*		*P. infestans*		*B. cinerea*		*S. tritici*	
**Extract**	500 μg/mL	50 μg/mL	500 μg/mL	50 μg/mL	125 μg/mL	42 μg/mL	125 μg/mL	42 μg/mL	125 μg/mL	42 μg/mL
CH_2_Cl_2_	77 ± 13	97 ± 0	100 ± 0	25 ± 2	96 ± 3	22 ± 10	89 ± 1	69 ± 8	73 ± 4	18 ± 19
**Compounds**	100 μM	1 μM	100 μM	1 μM	125 μg/mL	42 μg/mL	125 μg/mL	42 μg/mL	125 μg/mL	42 μg/mL
**1**	−7 ± 23	23 ± 34	−5 ± 26	3 ± 25	−68 ± 36	1 ± 19	−56 ± 40	−24 ± 11	6 ± 9	−1 ± 13
**2**	60 ± 2	−79 ± 17	24 ± 14	−25 ± 27	−32 ± 2	−14 ± 5	−38 ± 12	−20 ± 3	−24 ± 15	−1 ± 4
**7**	63 ± 2	−33 ± 73	−52 ± 23	−2 ± 28	81 ± 1	2 ± 3	95 ± 4	99 ± 0	88 ± 3	74 ± 10
**9**	96 ± 0	40 ± 2	67 ± 8	20 ± 18	71 ± 14	23 ± 12	0 ± 30	26 ± 5	76 ± 11	−16 ± 10
**10**	99 ± 0	n.d.	99 ± 0	8 ± 16	58 ± 12	86 ± 1	58 ± 13	56 ± 5	115 ± 6	13 ± 11
Positive control	100 μM chloramphenicol		100 μM chloramphenicol		42 μM terbinafine		42 μM epoxiconazole		42 μM epoxiconazole	
	100 ± 0		100 ± 0		87 ± 5	67 ± 8	99 ± 2	100 ± 0	97 ± 0	97 ± 0

^a^ Negative values indicate an increase of bacterial growth in comparison to the negative control (0% inhibition).

**Table 4:** Antibacterial activity of compounds from *D. melanoxylon* against human pathogens determined by agar diffusion assay (inhibition zone [mm]) and microdilution (MIC [μg/mL]).

	*B. subtilis*	*S. aureus*	*E. coli*	*P. aeruginosa*	*P. aeruginosa*	*S. aureus* (MRSA)		*E. faecalis* (VRE)		*M. vaccae*	
Compounds	6633 B1	511 B3	458 B4	SG137 B7	K799/61 B9	134/93 R9		1528 R10		10670 M4	
(1 mg/mL)	[mm]	[mm]	[mm]	[mm]	[mm]	[mm]	[μg/mL]	[mm]	[μg/mL]	[mm]	[μg/mL]
**1**	10	11	13P	0	0	0/A	n.t.	0	n.t.	15p	n.t.
**2**	11/13p	10	0	0/A	0	11	n.t.	11	n.t.	12	n.t.
**7**	23	23	14P	0	0	24	1.56	22F	1.56	27	0.78
**9**	24	25	15P	0	0/A	26	3.12	18/23p/F	6.25	27/33p	1.56
**10**	20/23P	20/23p-P	14P	0	0	21	25	17/25p/F	25	20/32p	3.12
CIP ^a^	29 EK	18	23/31p	25	28/35p	0	12.5	16F	0.78	20p	0.2
DMSO ^b^	11P	13P	12P	12P	12P	11P	>100	12p-P	100	11P	100

^a^ CIP: Ciprofloxacin, positive control tested at concentration of 5 μg/mL; ^b^ negative control; p = partial inhibition (few colonies visible within inhibition zone), P = partial inhibition (many colonies visible within inhibition zone), F = facilitation, A = indication of inhibition, n.t. = not tested.

## Data Availability

Additional information related to this manuscript can be found in the supporting information. Further data are available on request. Data is not publicly available due to privacy.

## Supplementary Materials

The following supporting information can be downloaded at: https://www.mdpi.com/article/10.3390/metabo13060678/s1, Figure S1: NMR, HRMS, UV and CD spectra of compound **1**; Figure S2: NMR, HRMS, UV and CD spectra of compound **2**; Figure S3: NMR and HRMS spectra of compound **3**; Figure S4: NMR and HRMS spectra of compound **4**; Figure S5: NMR and HRMS spectra of compound **5**; Figure S6: NMR and HRMS spectra of compound **6**; Figure S7: NMR, UV and CD spectra of compound **7**; Figure S8: NMR, UV and CD spectra of compound **8**; Figure S9: NMR, HRMS, UV and CD spectra of compound **9**; Figure S10: NMR, HRMS, UV and CD spectra of compound **10**; Figure S11: NMR and HRMS spectra of compound **11**; Figure S12: NMR and HRMS spectra of compound **12**; Figure S13: NMR and HRMS spectra of compound **13**; Figure S14: NMR and HRMS spectra of compound **14**; Table S1: NMR data of compound **1**; Table S2: NMR data of compound **2**; Table S3: NMR data of compound **3**; Table S4: NMR data of compound **4**; Table S5: NMR data of compound **5**; Table S6: NMR data of compound **6**; Table S7: NMR data of compound **7**; Table S8: NMR data of compound **8**; Table S9: NMR data of compound **9**; Table S10: NMR data of compound **10**; Table S11: NMR data of compound **11**; Table S12: NMR data of compound **12**; Table S13: NMR data of compound **13**; Table S14: NMR data of compound **14**; Table S15: Cytotoxic activities of crude extract of *D. melanoxylon* against human cancer cell lines; Table S16: Antifungal activity of compounds from *D. melanoxylon* against human pathogens.
