# Supplementary material for: Prenylated Isoflavanones with Antimicrobial Potential from the Root Bark of Dalbergia melanoxylon
Source: Metabolites. 2023 May 23;13(6):678. doi: 10.3390/metabo13060678 (PMC10304121; doi:10.3390/metabo13060678)
Supplement: Supplementary file 1 [file metabolites-13-00678-s001.zip › metabolites-2372124-supplementary.pdf]

Supporting information

Prelated isoflavanones with antimicrobial potential from the root bark of *Dalbergia melanoxylon*

Duncan Mutiso Chalo[1,2,3], Katrin Franke[2,4,5*], Vaderament-A. Nchiozem-Ngnitedem[6], Esezah Kakudidi [1], Hannington Origa-Oryem[1], Jane Namukobe[7], Florian Kloß[8], Abiy Yenesew[6*], Ludger A. Wessjohann[2,5*]

[1]*Department of Plant Sciences, Microbiology and Biotechnology, Makerere University P.O. Box 7062 Kampala, Uganda;*
dunmutiso6@gmail.com (D.M.C.); esezahk@gmail.com (E.K.); horyemoriga@gmail.com (H.O.-O.)
[2]*Leibniz Institute of Plant Biochemistry, Weinberg 3, 06120 Halle (Saale), Germany*
[3]*Department of Biology, University of Nairobi, P.O. Box 30197-0100, Nairobi, Kenya*
[4]*Institute of Biology/Geobotany and Botanical Garden, Martin Luther University Halle-Wittenberg, 06108 Halle, Germany*
[5]*German Centre for Integrative Biodiversity Research (iDiv) Halle-Jena-Leipzig, 04103 Leipzig, Germany*
[6]*Department of Chemistry, University of Nairobi, P.O. Box 30197-0100, Nairobi, Kenya; n.vaderamentalexe@gmail.com*
[7]*Department of Chemistry, Makerere University, P.O. Box 7062 Kampala, Uganda; jnamukobe@gmail.com*
[8]*Transfer Group Anti-Infectives, Leibniz Institute for Natural Product Research and Infection Biology, Hans Knöll Institute, Beutenbergstr. 11a, 07745, Jena, Germany; florian.kloss@leibniz-hki.de*
**Correspondence: kfranke@ipb-halle.de (K.F.); ayenesew@uonbi.ac.ke (A.Y.); wessjohann@ipb-halle.de (L.A.W.)*

Content Page

Figure S1_1: ^{1}H NMR spectrum (500 MHz, CD_3OD) of compound **1** 3
Figure S1_2: ^{13}C NMR spectrum (125 MHz, CD_3OD) of compound **1** 3
Figure S1_3: HSQCAD spectrum (125 MHz, CD_3OD) of compound **1** 4
Figure S1_4: HMBC spectrum (125 MHz, CD_3OD) of compound **1** 4
Figure S1_5: NOESY spectrum (125 MHz, CD_3OD) of compound **1** 5
Figure S1_6: Negative HRMS of compound **1** 5
Figure S1_7: UV and CD spectra (MeOH) of compound **1** 6
Figure S2_1: ^{1}H NMR spectrum (500 MHz, CD_3OD) of compound **2** 7
Figure S2_2: ^{13}C NMR spectrum (125 MHz, CD_3OD) of compound **2** 7
Figure S2_3: HSQCAD spectrum (125 MHz, CD_3OD) of compound **2** 8
Figure S2_4: HMBC spectrum (125 MHz, CD_3OD) of compound **2** 8
Figure S2_5: COSY spectrum of compound **2** 9
Figure S2_6: ROESY spectrum of compound **2** 9
Figure S2_7: Negative ion HRMS spectrum of compound **2** 10
Figure S2_8: UV and CD spectra (MeOH) of compound **2** 10
Figure S3_1: ^{1}H NMR spectrum (600 MHz, CD_3OD) of compound **3** 12
Figure S3_2: HSQCAD spectrum (125 MHz, CD_3OD) of compound **3** 12
Figure S3_3: HMBC spectrum (125 MHz, CD_3OD) of compound **3** 13
Figure S3_4: COSY spectrum (125 MHz, CD_3OD) of compound **3** 13
Figure S3_5: Negative HRMS of compound **3** 14
Figure S4_1: ^{1}H NMR spectrum (600 MHz, CD_3OD) of compound **4** 15
Figure S4_2: HSQCAD_ (NUS 50%) spectrum (600 MHz, CD_3OD) of compound **4** 15
Figure S4_3: HMBC_ (NUS 50%) spectrum (600 MHz, CD3OD) of compound **4** 16
Figure S4_4: Negative ion ESI-HRMS spectrum of compound **4** 16
Figure S5_1:^{1}H NMR spectrum (600 MHz, CD_3OD) of compound **5** 18
Figure S5_2: HQSCAD_ (NUS 50%) spectrum (600 MHz, CD_3OD) of compound **5** 18
Figure S5_3: HMBC_ (NUS 50%) spectrum (600 MHz, CD_3OD) of compound **5** 19
Figure S5_4: Negative ion ESI-HRMS spectrum of compound **5** 19
Figure S6_1: ^{1}H NMR spectrum (400 MHz, CD_3OD) of compound **6** 21
Figure S6_2: ^{13}C NMR spectrum (125 MHz, CD_3OD) of compound **6** 21
Figure S6_3: HSQCAD spectrum (125 MHz, CD_3OD) of compound **6** 22
Figure S6_4: HMBC spectrum (125 MHz, CD_3OD) of compound **6** 22
Figure S6_5: COSY spectrum (125 MHz, CD_3OD) of compound **6** 23
Figure S6_6: NOESY spectrum (125 MHz, CD_3OD) of compound **6** 23
Figure S6_7: Negative ion ESI-HRMS spectrum of compound **6** 24
Figure S7_1: ^{1}H NMR spectrum (400 MHz, CD_3OD) of kenusanone H (**7**) 25

Figure S7_2: ^{13}C NMR spectrum (125 MHz, CD_3OD) of kenusanone H (**7**)....25
Figure S7_3: HSQCAD spectrum (125 MHz, CD_3OD) of kenusanone H (**7**)....26
Figure S7_4: HMBC spectrum (125 MHz, CD_3OD) of kenusanone H (**7**)....26
Figure S7_5: NOESY spectrum (125 MHz, CD_3OD) of kenusanone H (**7**)....27
Figure S7_6: UV and CD data of kenusanone H (**7**)....27
Figure S8_1: ^{1}H NMR spectrum (400 MHz, CD_3OD) of kenusanone F (**8**)....29
Figure S8_2: ^{13}C NMR spectrum (125 MHz, CD_3OD) of kenusanone F (**8**)....29
Figure S8_3: HSQCAD spectrum (125 MHz, CD_3OD) of kenusanone F (**8**)....30
Figure S8_4: HMBC spectrum (125 MHz, CD_3OD) of kenusanone F (**8**)....30
Figure S8_5: UV and CD data of kenusanone F (**8**)....31
Figure S9_1: ^{1}H NMR spectrum (500 MHz, CD_3OD) of tomentosanol B (**9**)....32
Figure S9_2: ^{13}C NMR spectrum (125 MHz, CD_3OD) of tomentosanol B (**9**)....32
Figure S9_3: HSQCAD spectrum (125 MHz, CD_3OD) of tomentosanol B (**9**)....33
Figure S9_4: HMBC spectrum (125 MHz, CD_3OD) of tomentosanol B (**9**)....33
Figure S9_5: Negative ion ESI-HRMS spectrum of tomentosanol B (**9**)....34
Figure S9_6: UV and CD data of tomentosanol B (**9**)....34
Figure S10_1: ^{1}H NMR spectrum (125 MHz, acetone-d6) of sophoraisoflavanone A (**10**)....36
Figure S10_2: ^{13}C NMR spectrum (125 MHz, acetone-d6) of sophoraisoflavanone A (**10**)....36
Figure S10_3: Positive HRMS of sophoraisoflavanone A (**10**)....37
Figure S10_4: UV and CD data of sophoraisoflavanone A (**10**)....37
Figure S11_1: ^{1}H NMR spectrum (400 MHz, CD_3OD) of methyl dalbergin (**11**)....39
Figure S11_2: ^{13}C NMR spectrum (125 MHz, CD_3OD) of methyl dalbergin (**11**)....39
Figure S11_3: HSQCAD spectrum (125 MHz, CD_3OD) of methyl dalbergin (**11**)....40
Figure S11_4: HMBC spectrum (125 MHz, CD_3OD) of methyl dalbergin (**11**)....40
Figure S11_5: ROESY spectrum (125 MHz, CD_3OD) of methyl dalbergin (**11**)....41
Figure S11_6: Positive ion ESI-HRMS spectrum of methyl dalbergin (**11**)....41
Figure S12_1: ^{1}H NMR spectrum (400 MHz, CD_3OD) of dalbergin (**11**)....43
Figure S12_2: HSQC spectrum (400 MHz, CD_3OD) of dalbergin (**12**)....43
Figure S12_3: HMBC spectrum (400 MHz, CD_3OD) of dalbergin (**12**)....44
Figure S12_4: ROESY spectrum (400 MHz, CD_3OD) of dalbergin (**12**)....44
Figure S12_5: Positive ion ESI-HRMS spectrum of dalbergin (**12**)....45
Figure S13_1: ^{1}H NMR spectrum (400 MHz, CD_3OD) of melannein (**13**)....46
Figure S13_2: HSQCAD spectrum (125 MHz, CD_3OD) of melannein (**13**)....46
Figure S13_3: HMBC spectrum (125 MHz, CD_3OD) of melannein (**13**)....47
Figure S13_4: ROESY spectrum (125 MHz, CD_3OD) of melannein (**13**)....48
Figure S13_5: Negative ion ESI-HRMS spectrum of melannein (**13**)....49
Figure S14_1: ^{1}H NMR spectrum (400 MHz, $CDCl_3$ and few drops CD_3OD) of compound **14**....50
Figure S14_2: HSQCAD spectrum (400 MHz, $CDCl_3$ and few drops CD_3OD) of compound **14**....50
Figure S14_3: Negative HRMS for compound **14**....51

Table S1: NMR data of compound **1**....6
Table S2: NMR data of compound **2**....11
Table S3: NMR data of compound **3**....14
Table S4: NMR data of compound **4**....17
Table S5: NMR data of compound **5**....20
Table S6: NMR data of compound **6**....24
Table S7: NMR data of kenusanone H (**7**)....28
Table S8: NMR data of kenusanone F (**8**)....31
Table S9: NMR of tomentosanol B (**9**)....35
Table S10: NMR data of sophoraisoflavanone A (**10**)....38
Table S11: NMR data of methyl dalbergin (**11**)....42
Table S12: NMR data of dalbergin (**12**)....45
Table S13: NMR data of melannein (**13**)....49
Table S14: NMR data of CDU 004 (Compound **14**)....51
Table S15: Cytotoxic activities of crude extract of *D. melanoxylon* against human cancer cell lines....52
Table S16: Antifungal activity of compounds from *D. melanoxylon* against human pathogens....52

(3*S*)-3,4′,5,7-Tetrahydroxyl-2′-methoxy-3′-(4-hydroxyl-3-methylbut-2-enyl)-isoflavanone (1)

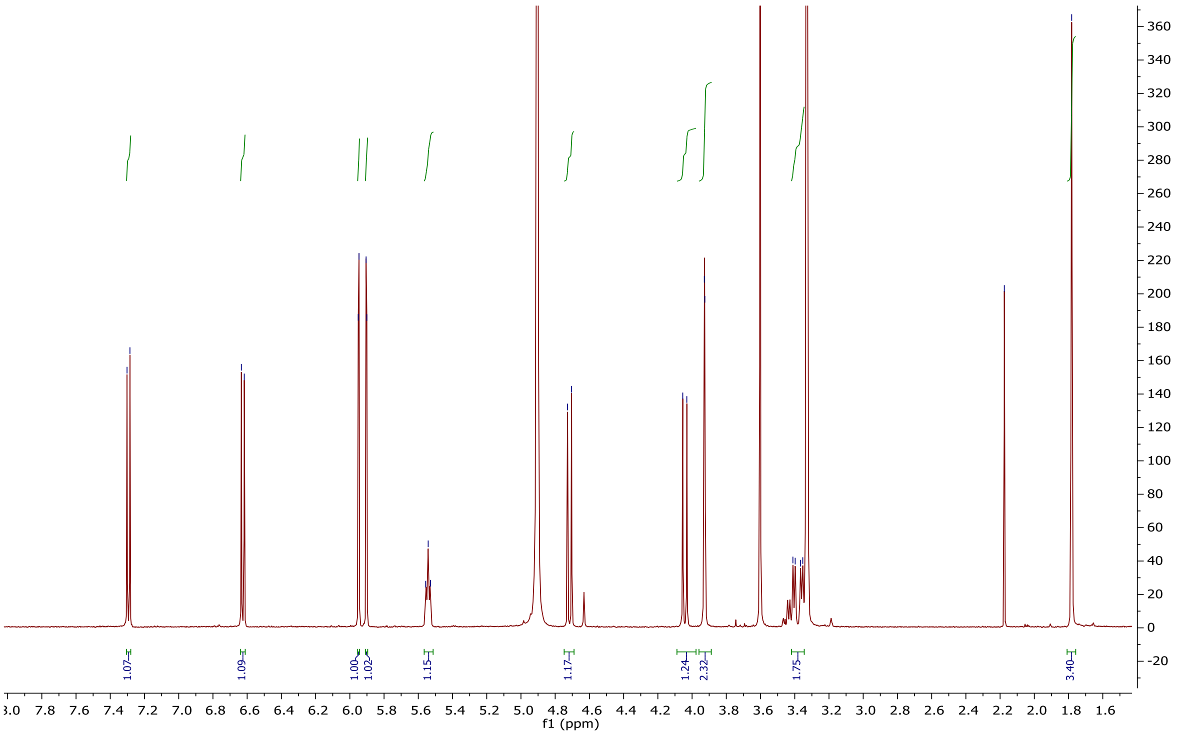

Figure S1_1: ^{1}H NMR spectrum (500 MHz, CD_3OD) of compound **1**

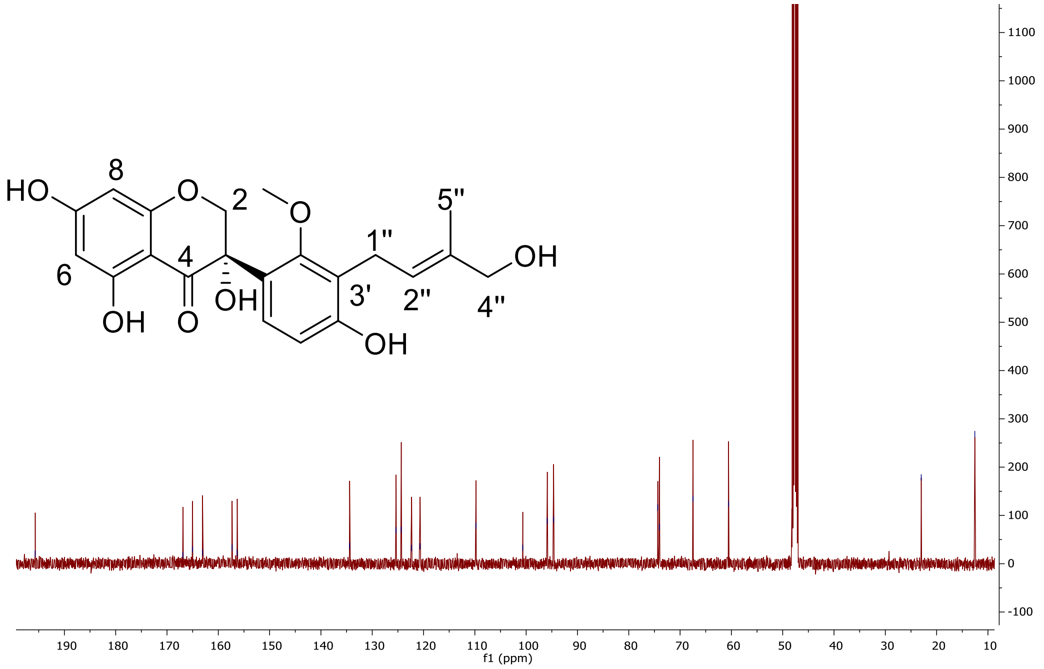

Figure S1_2: ^{13}C NMR spectrum (125 MHz, CD_3OD) of compound **1**

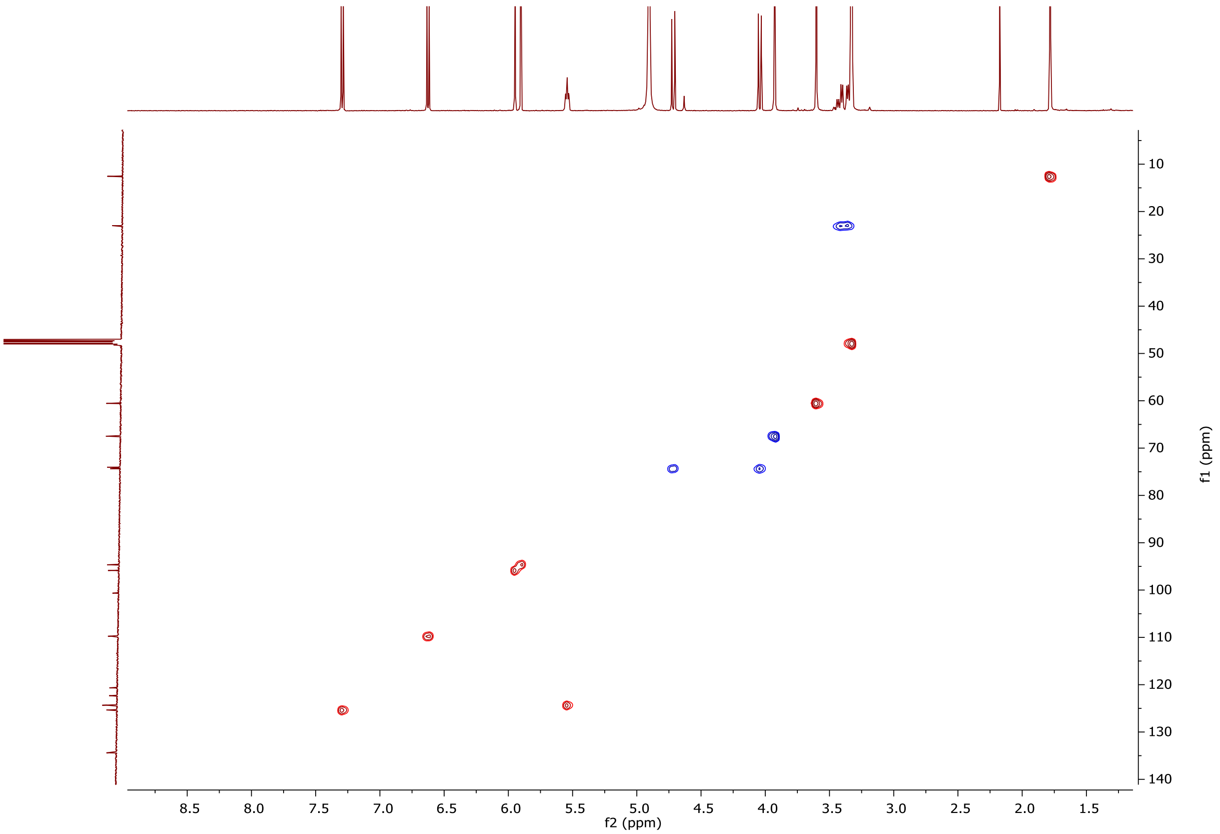

Figure S1_3: HSQCAD spectrum (125 MHz, CD_3OD) of compound **1**

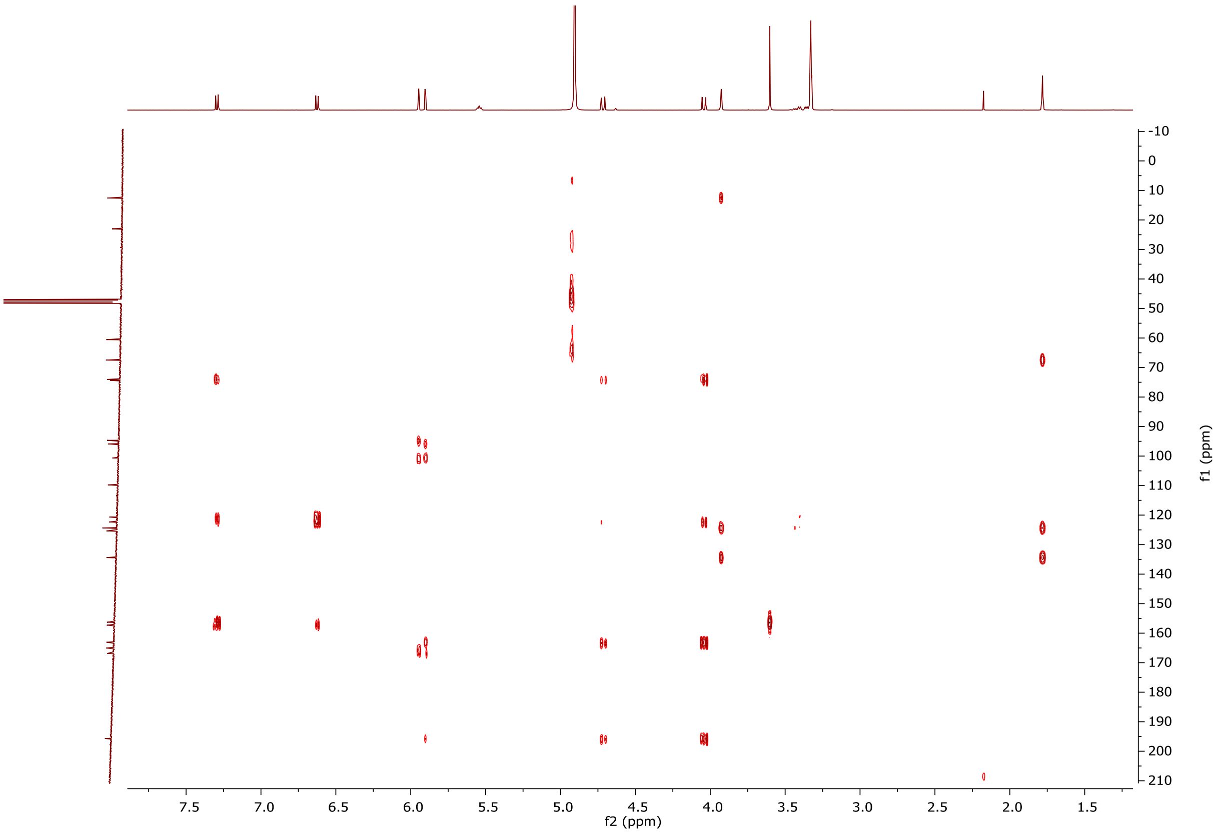

Figure S1_4: HMBC spectrum (125 MHz, CD_3OD) of compound **1**

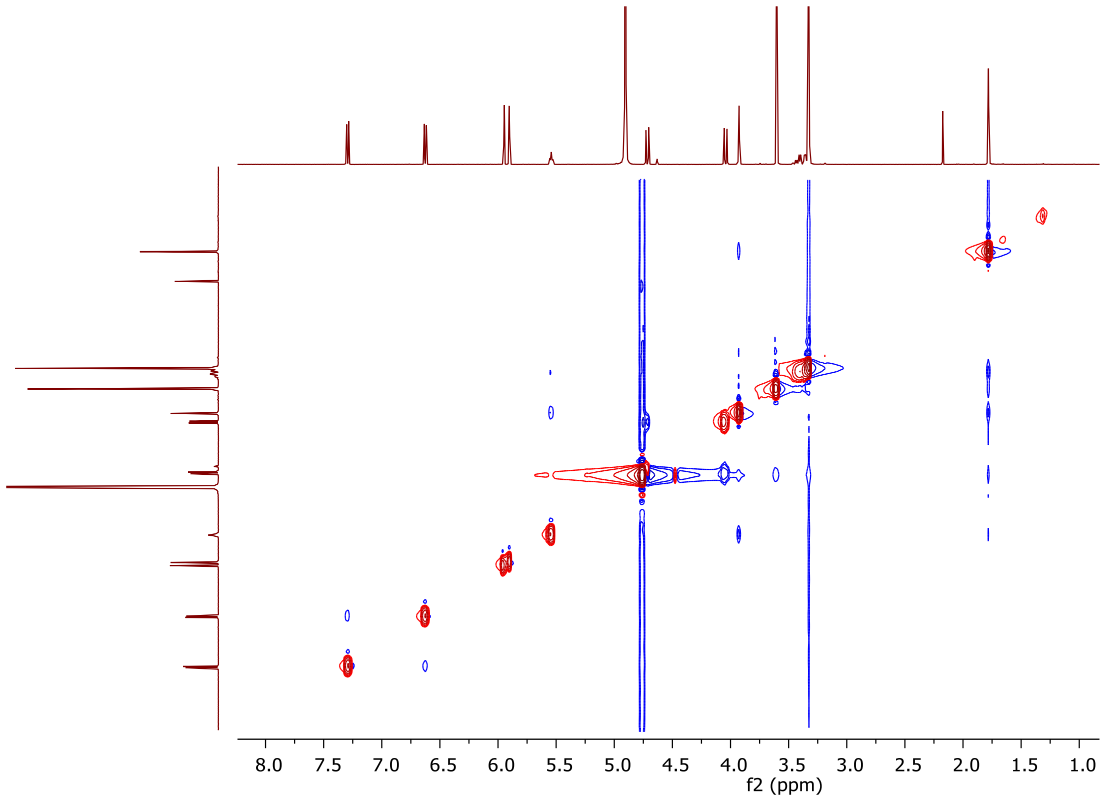

Figure S1_5: NOESY spectrum (125 MHz, CD_3OD) of compound **1**

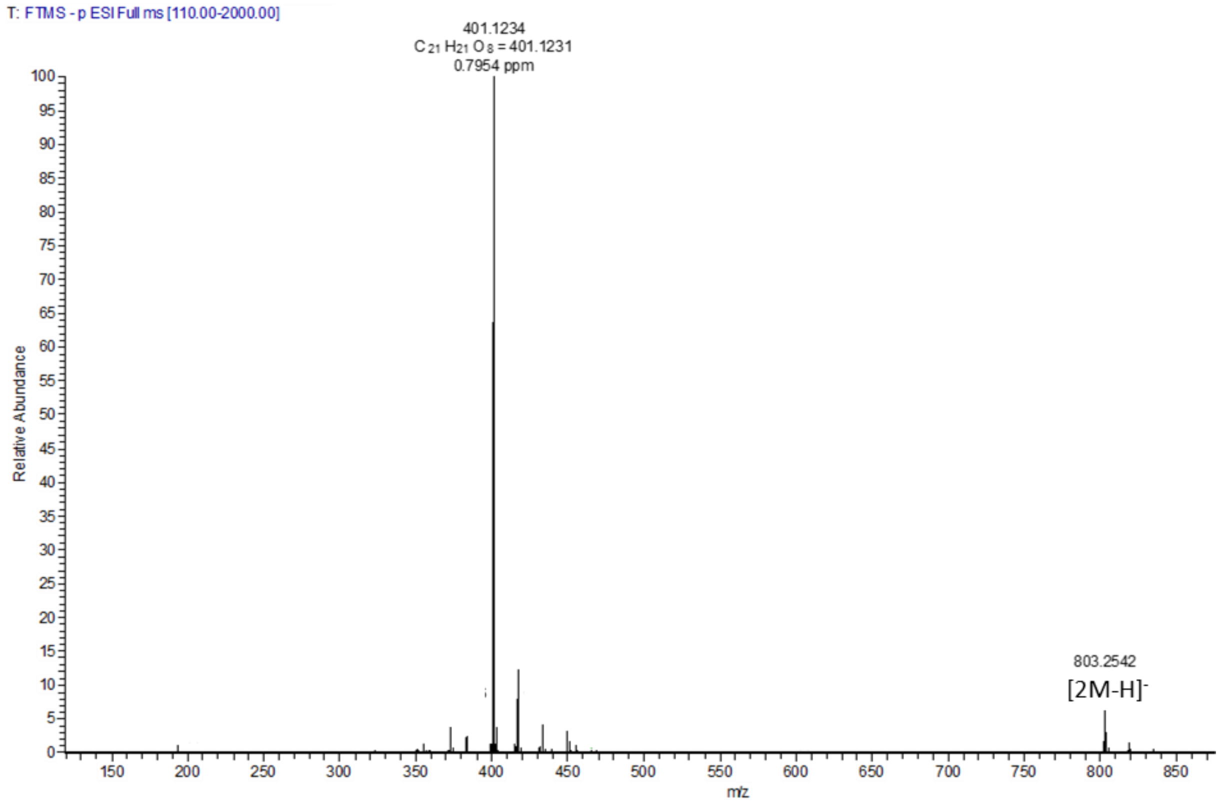

Figure S1_6: Negative ion ESI-HRMS spectrum of compound **1**

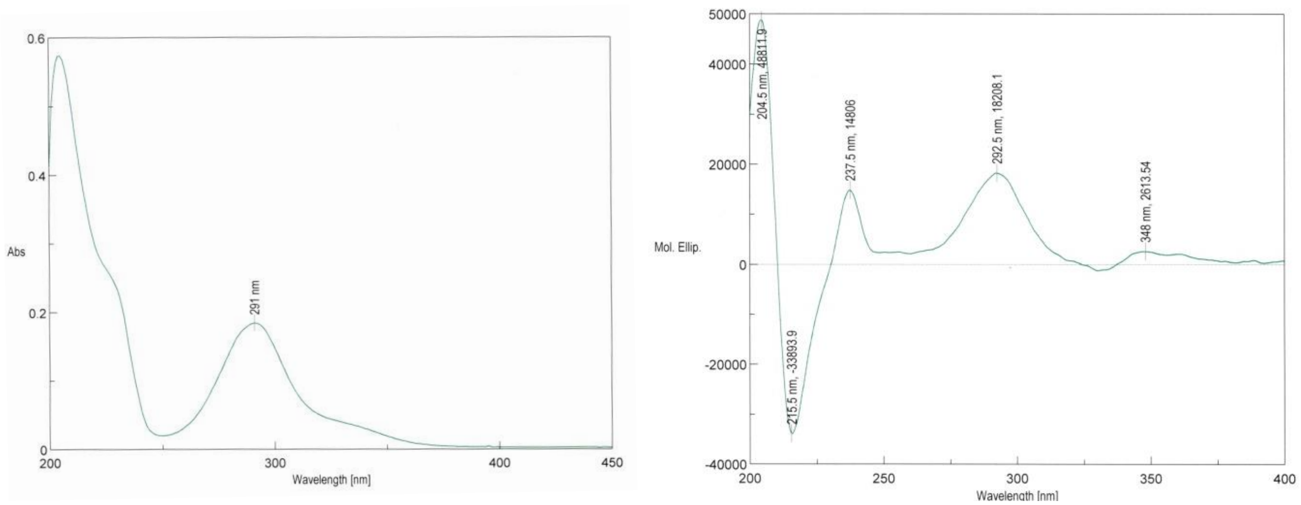

Figure S1_7: UV and CD spectra (MeOH) of compound **1**

Table S1: NMR data of compound **1**

No	δ_H,* *mult* (*J* in Hz)	δ_C		HMBC	COSY	NOESY
2	A: 4.70, *d* (11.8)	75.8	CH_2	C2B, C3, C9, C1′, C4	H2B	H2B, 2′-OCH_3
	B: 4.03, *d* (11.8)			C2A, C3, C1′, C9, C4	H2A	H2A
3		75.4	C			
4		197.1	C=O			
5		166.4	C			
6	5.93, *d* (2.0)	97.3	CH	C8, C10,	H8	
7		168.3	C			
8	5.88, *d* (2.0)	96.1	CH	C6, C10, C7, C4	H6	
9		164.5	C			
10		102.1	C			
1′		123.7	C			
2′		157.7	C			
3′		122.1	C			
4′		158.7	C			
5′	6.61, *d* (8.5)	111.2	CH	C1′, C3′, C4′	H 6′	H6′
6′	7.27, *d* (8.5)	126.8	CH	C3, C1′, C2′, C4′, C3′	H 5′	H5′
1″	A: 3.40, *dd* (14.9/6.4)	24.4	CH_2	C3′, C2″	H 2″	
	B: 3.33, *dd* (14.9/6.4)					
2″	5.52, *t-like* (6.4)	125.8	CH	C4″, C5″	H 1″	H4″
3″		135.8	C			
4″	3.91, *s*	68.9	CH_2	C5″, C2″, C3″		H5″, H2″
5″	1.76, *s*	14.0	CH_3	C3″, C4″, C2″		H4″
2′-OCH_3	3.58, *s*	62.0	CH_3	C2′		H2A
5-OH	12.09, *s*					

*referenced to methanol-*d*4 solvent signal

(3*R*)-6-Geranyl-4′,5,7-trihydroxyl-2′-methoxy-3′-prenylisoflavanone (2)

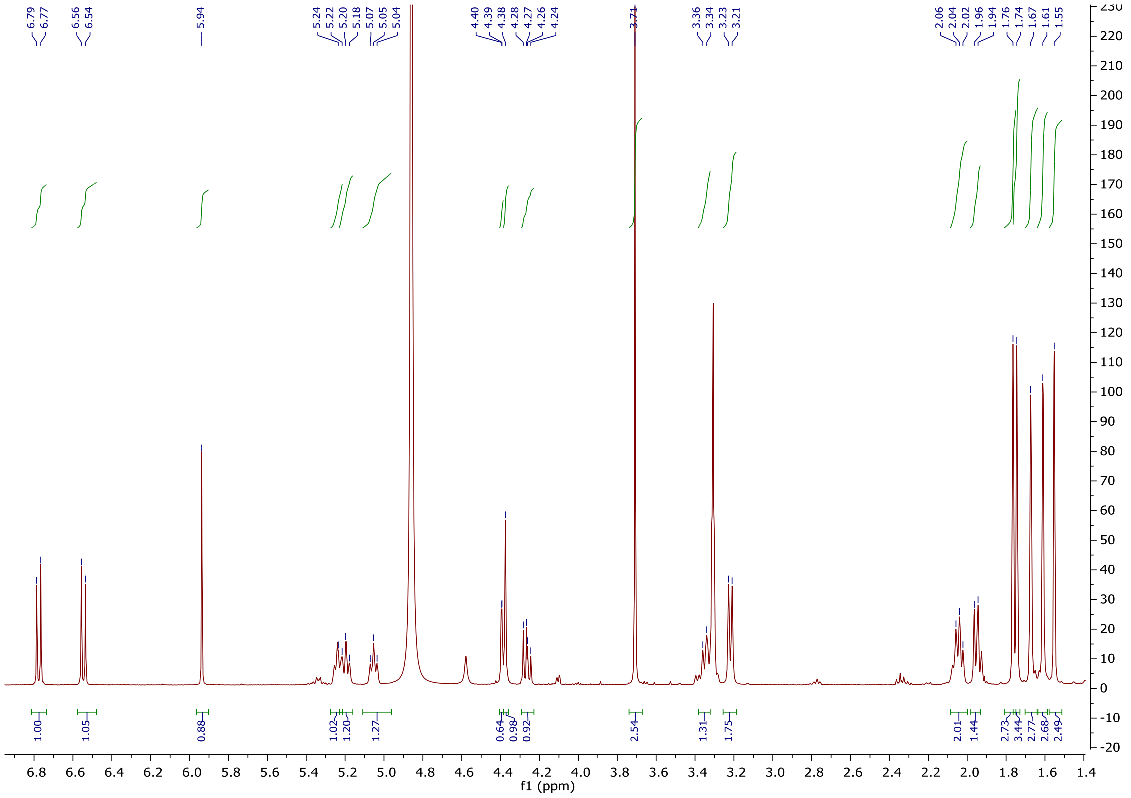

Figure S2_1: ^{1}H NMR spectrum (500 MHz, CD_3OD) of compound **2**

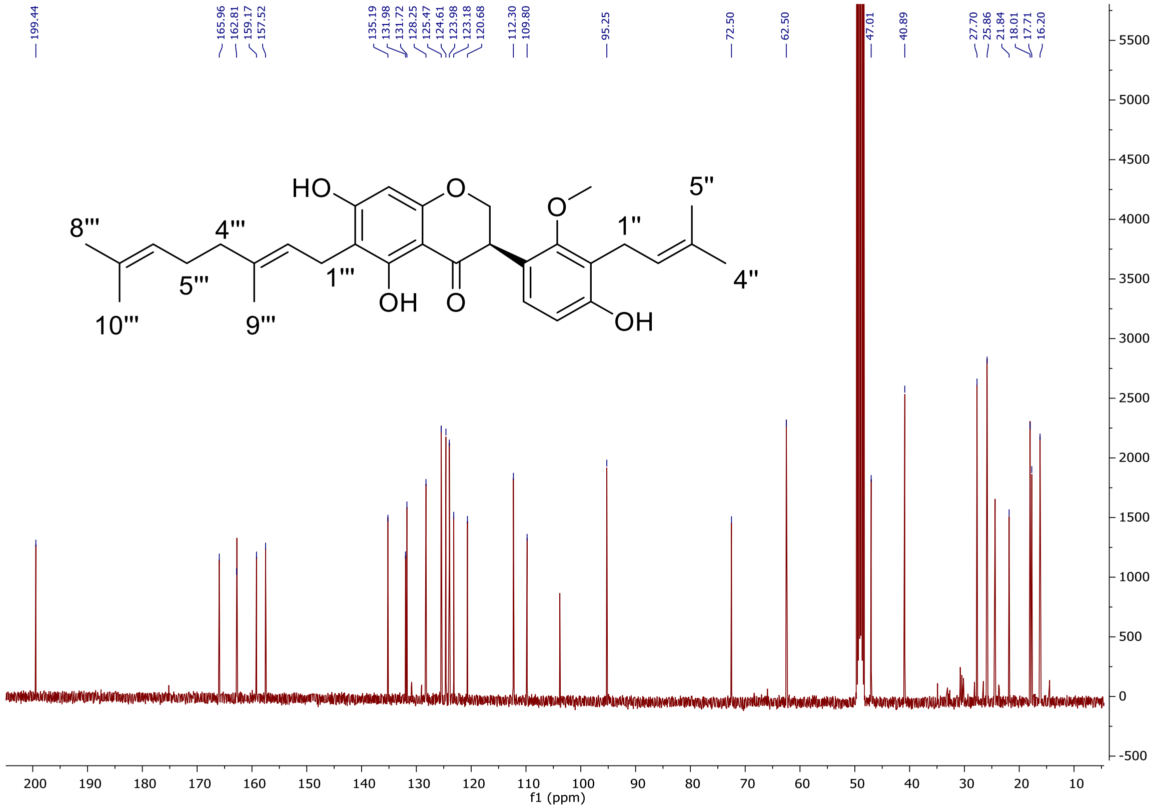

Figure S2-2: ^{13}C NMR spectrum (125 MHz, CD_3OD) of compound **2**

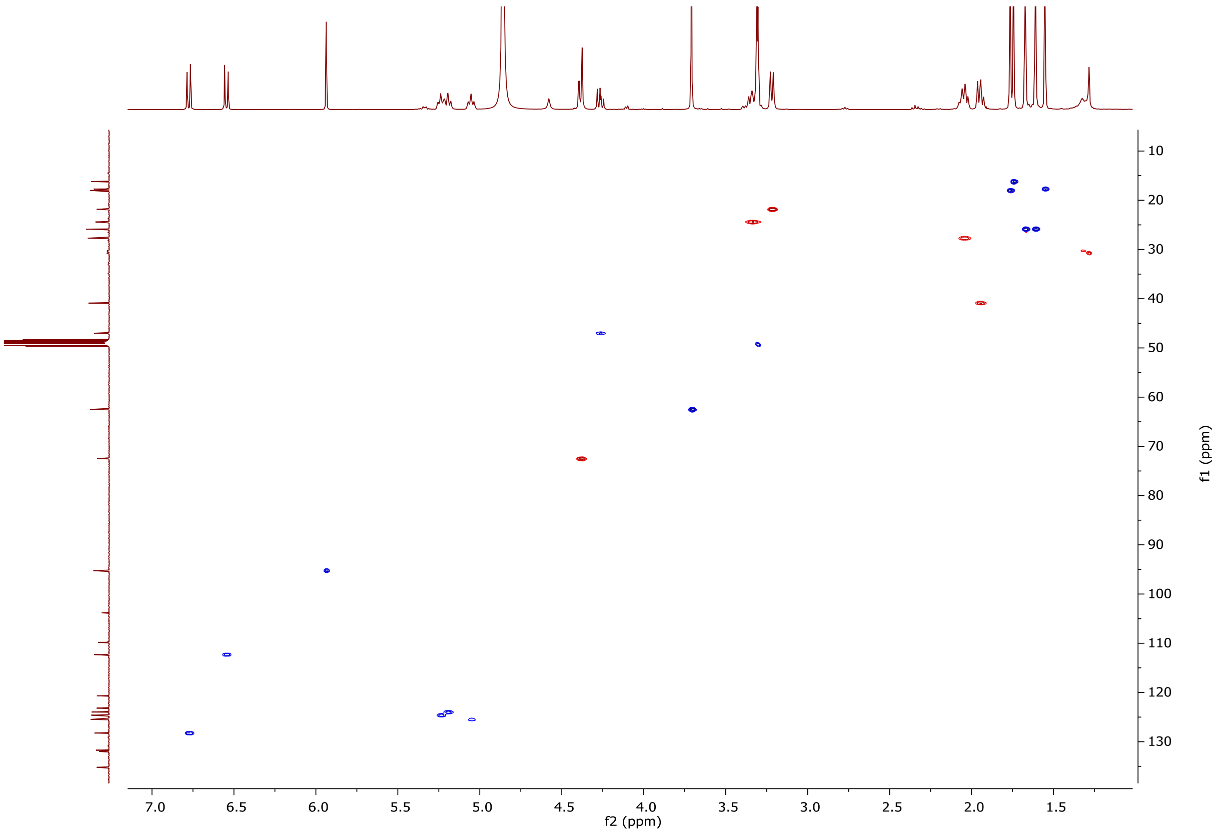

Figure S2_3: HSQCAD spectrum (125 MHz, CD_3OD) of compound **2**

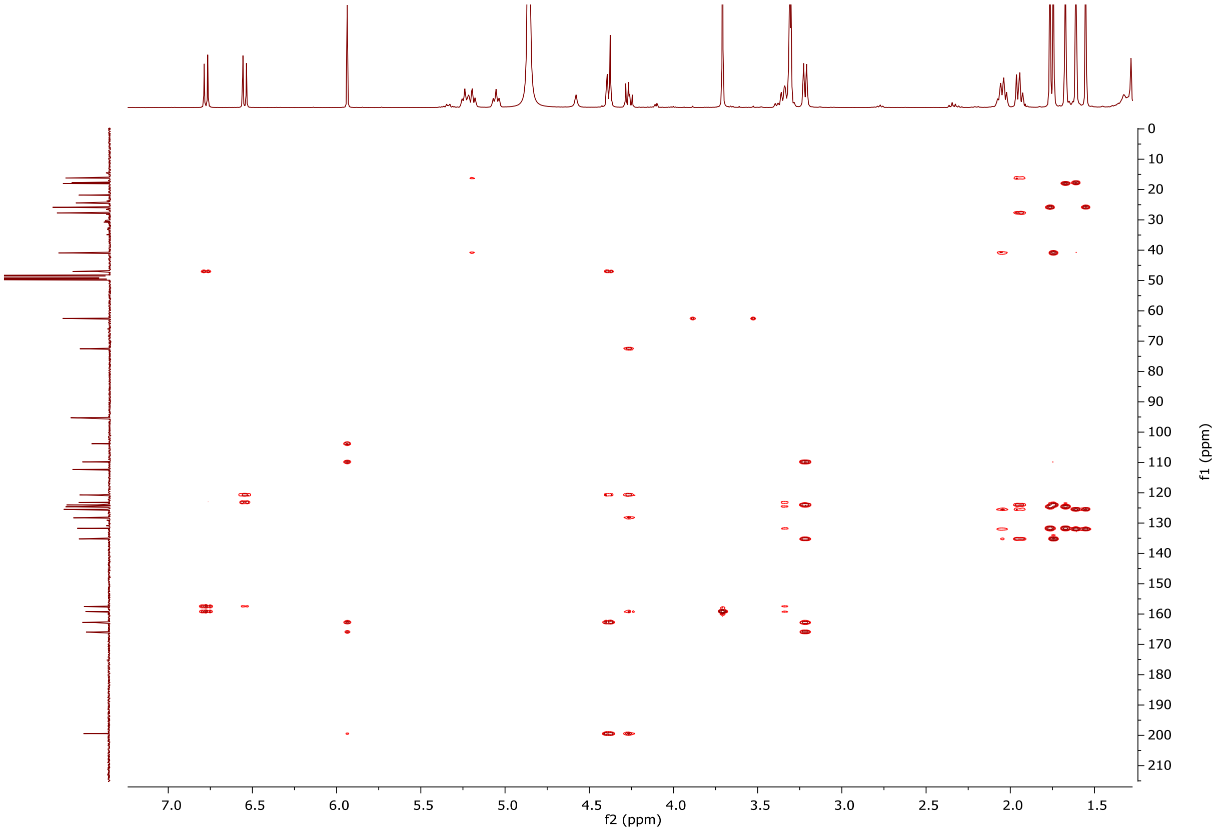

Figure S2_4: HMBC spectrum (125 MHz, CD_3OD) of compound **2**

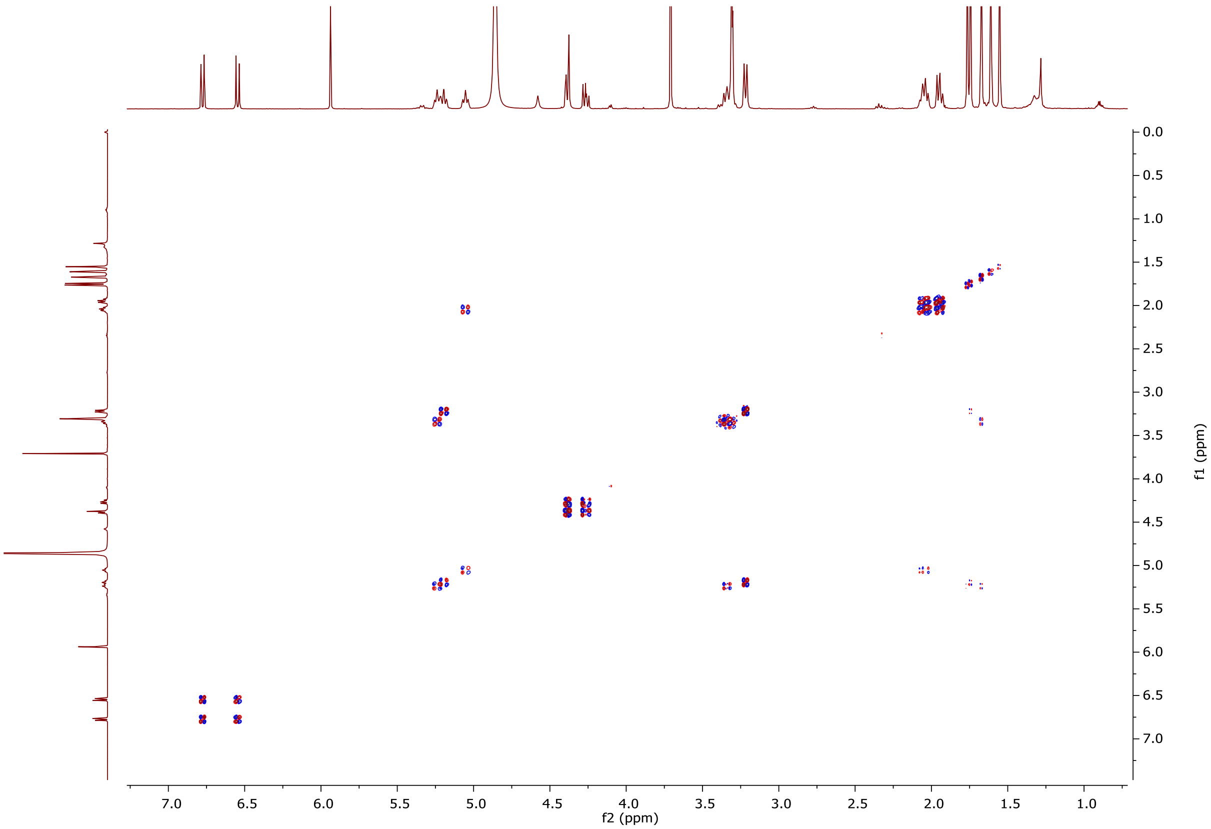

Figure S2_5: COSY spectrum of compound **2**

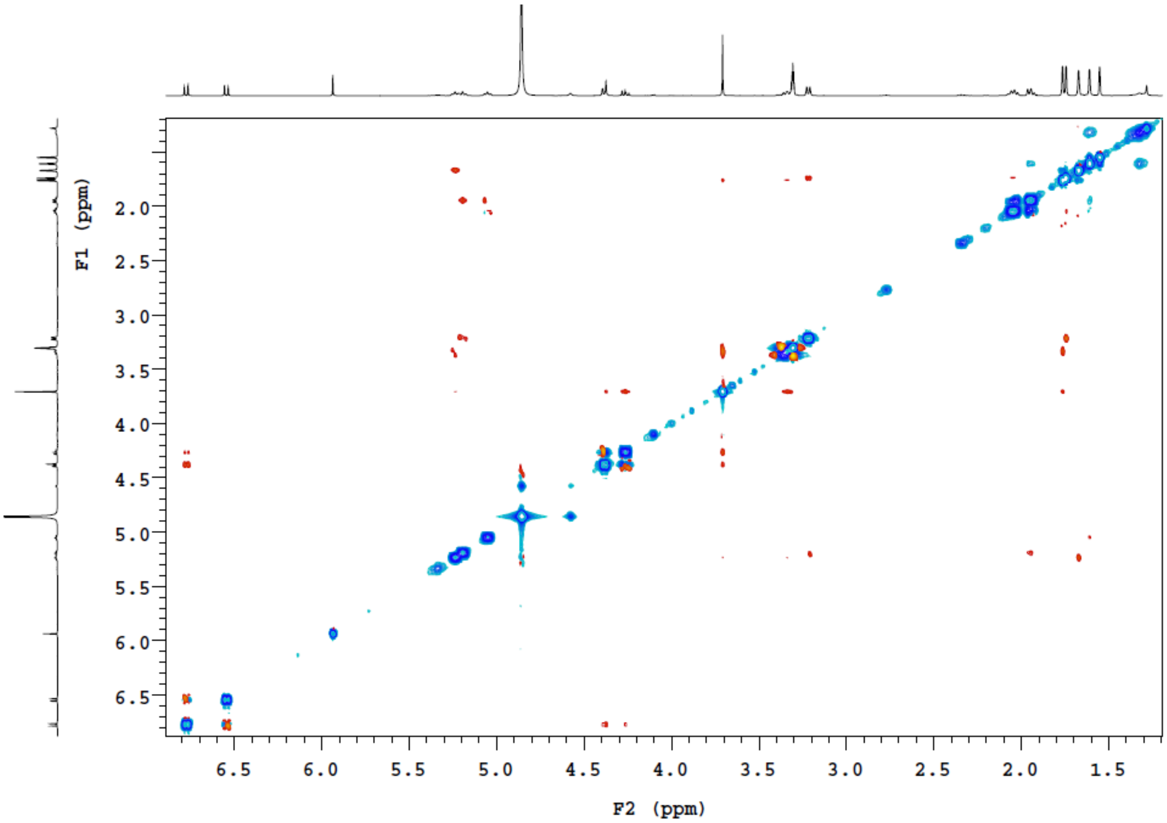

Figure S2_6: ROESY spectrum of compound **2**

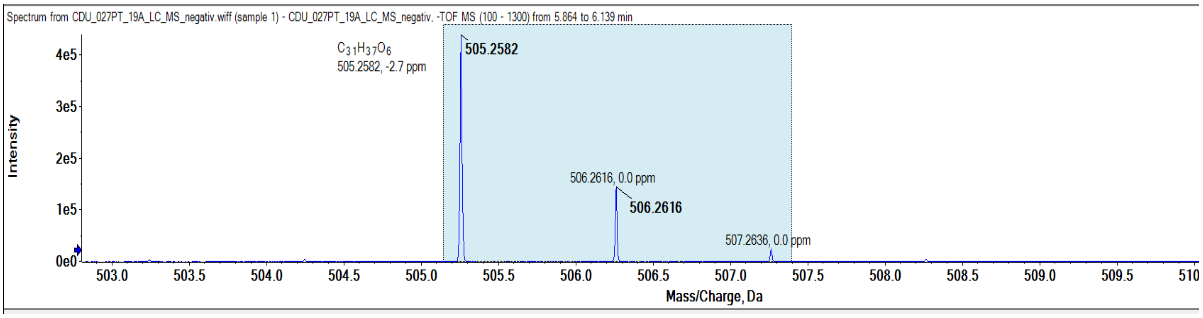

Figure S2_7: Negative ion ESI-HRMS spectrum of compound **2**

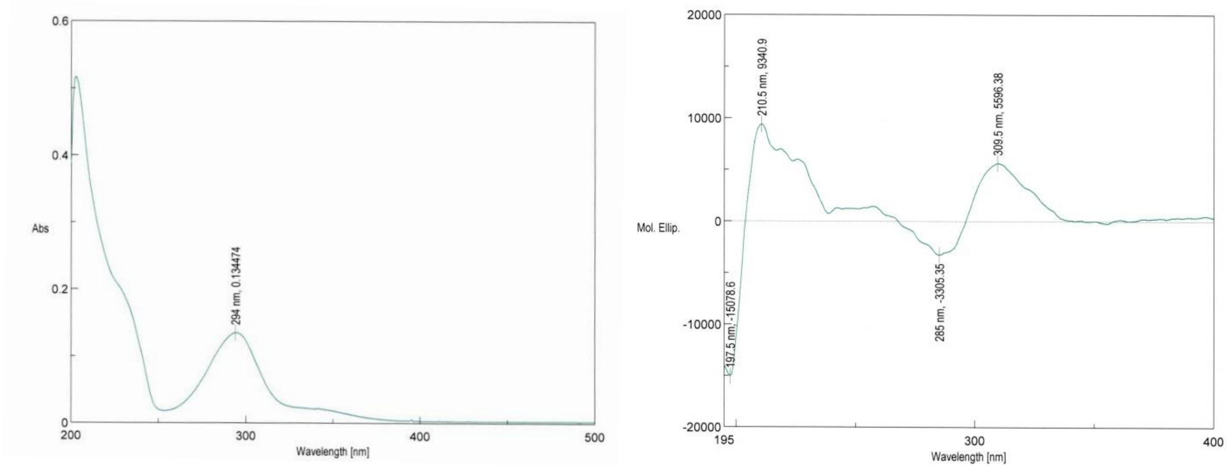

Figure S2_8: UV and CD spectra (MeOH) of compound **2**

Table S2: NMR data of compound **2**

No	δ_H, *mult* (*J* in Hz)	δ_C		HMBC (H → C)	COSY	NOESY
2	A: 4.40, *d* (8.9)	72.5	CH_2	C4, C3, C1′, C9	H2B, H3	H2B, H3
	B: 4.39, *d* (6.7)			C4, C3, C1′, C9	H2A, H3	H2A, H3
3	4.26, *dd* (8.9/6.7)	47.0	CH	C4, C2′ C1′, C6′	H2A/2B	H2A/2B
4		199.4	C=O			
5		162.8	C			
6		109.8	C			
7		166.0	C			
8	5.94, *s*	95.3	CH	C9, C10, C6, C4, C7		
9		162.8	C			
10		103.8	C			
1′		120.7	C			
2′		159.2	C			
3′		123.2	C			
4′		157.5	C			
5′	6.55, *d* (8.4)	112.3	CH	C1′, C6′, C3′, C4′	H6′	H6′
6′	6.78, *d* (8.4)	128.3	CH	C3, C5′, C1′, C4, C2′	H5′	H5′
1″	A: 3.37, *dd* (14.4/6.7)<br>B: 3.30 (under solvent signal)	24.4	CH_2	C4′, C2′, C2″, C3′, C3″	H2″	H5″
2″	5.24, *t-like* (6.7)	124.6	CH	C4″, C5″	H1″	H4″
3″		131.7	C			
4″	1.68, *s*	25.9	CH_3			H2 ″
5″	1.76, *s*	18.0	CH_3	C2″, C3″		H1″
1‴	3.22, *d* (7.2)	21.8	CH_2	C7, C5, C3‴, C2‴, C6	H2‴	H9‴
2‴	5.20, *t-like* (7.2)	124.0	CH	C9‴, C4‴, C1‴	H1‴	H4‴
3‴		135.2	C			
4‴	1.95, *m*	40.9	CH_2	C9‴, C5‴, C3‴, C6‴, C2‴	H5‴	H2‴, H5‴, H6‴
5‴	2.05, *m*	27.7	CH_2	C4‴, C6‴, C3‴	H6‴, H4‴	H4‴, H6‴
6‴	5.05, *br t* (6.8)	125.5	CH	C8‴, C10‴, C5‴	H5‴	H5‴, H4‴, H8‴
7‴		132.0	C			
8‴	1.61, *s*	25.9	CH_3	C10‴, C6‴, C7‴		H6‴
9‴	1.74, *s*	16.2	CH_3	C4‴, C3‴, C2‴		H1‴
10‴	1.55, *s*	17.7	CH_3	C8‴, C6‴, C5‴		
2″-OCH_3	3.71, *s*	62.5	CH_3	C2		H1″, H2″, H2B, H3
5-OH	12.41, *s*					

6-((2*E*,5*E*)-7-Hydroxyl-3,7-dimethyl-octa-2,5-dienyl)-4′,5,7-trihydroxy-2′-methoxy-3′-prenylisoflavanone (3)

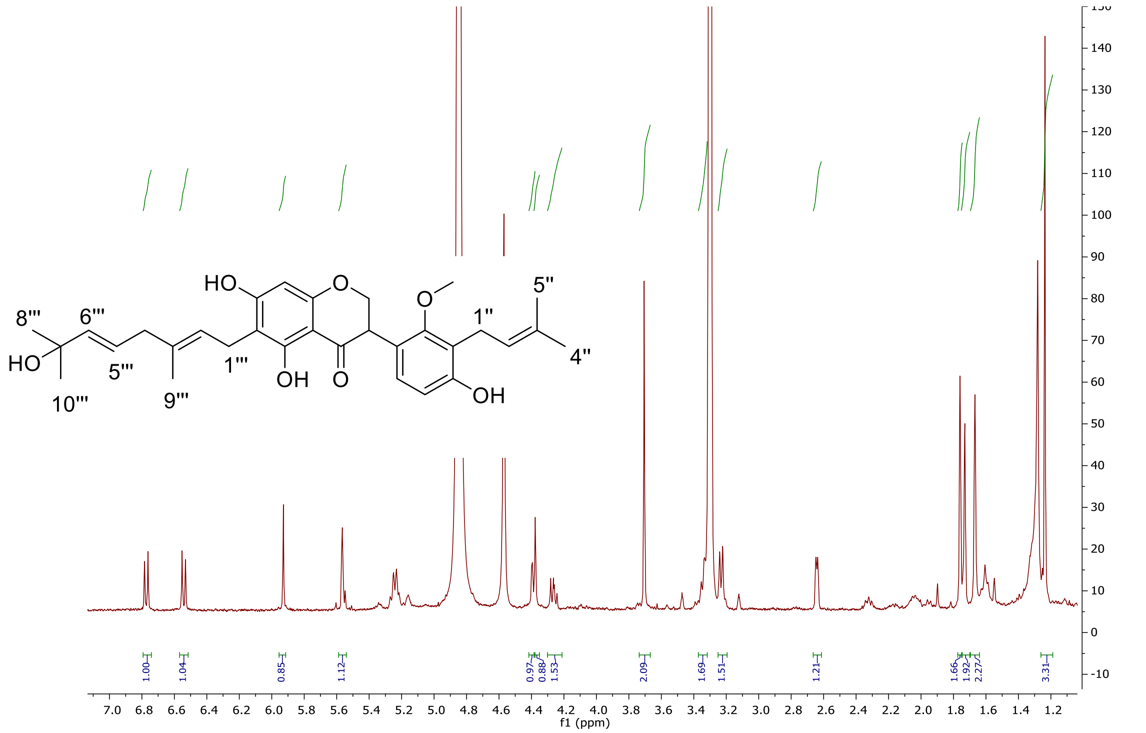

Figure S3_1: ^{1}H NMR spectrum (600 MHz, CD_3OD) of compound **3**

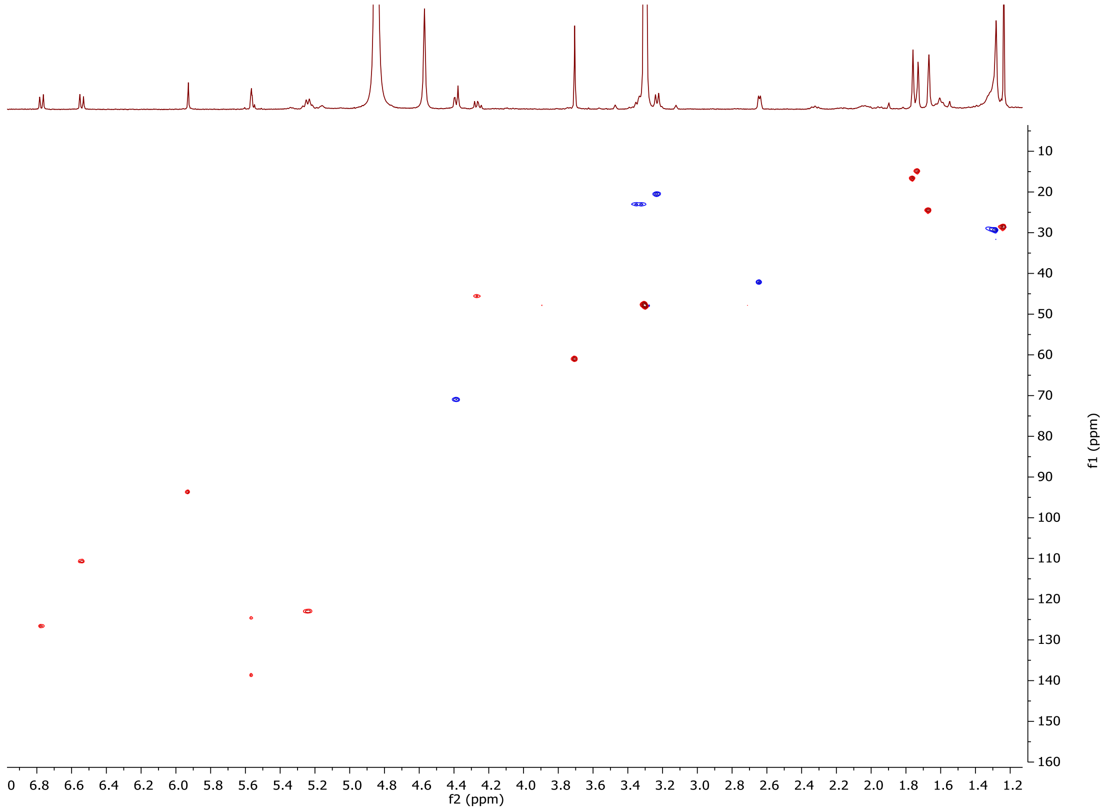

Figure S3_2: HSQCAD spectrum (125 MHz, CD_3OD) of compound **3**

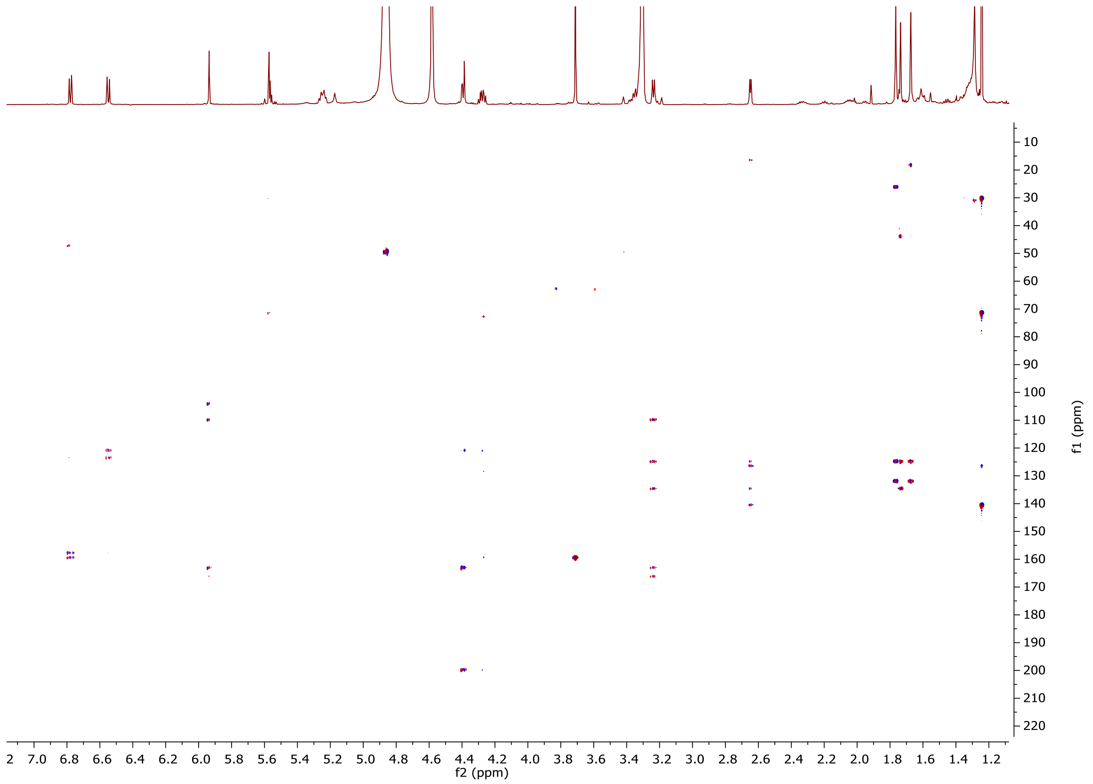

Figure S3_3: HMBC spectrum (125 MHz, CD_3OD) of compound **3**

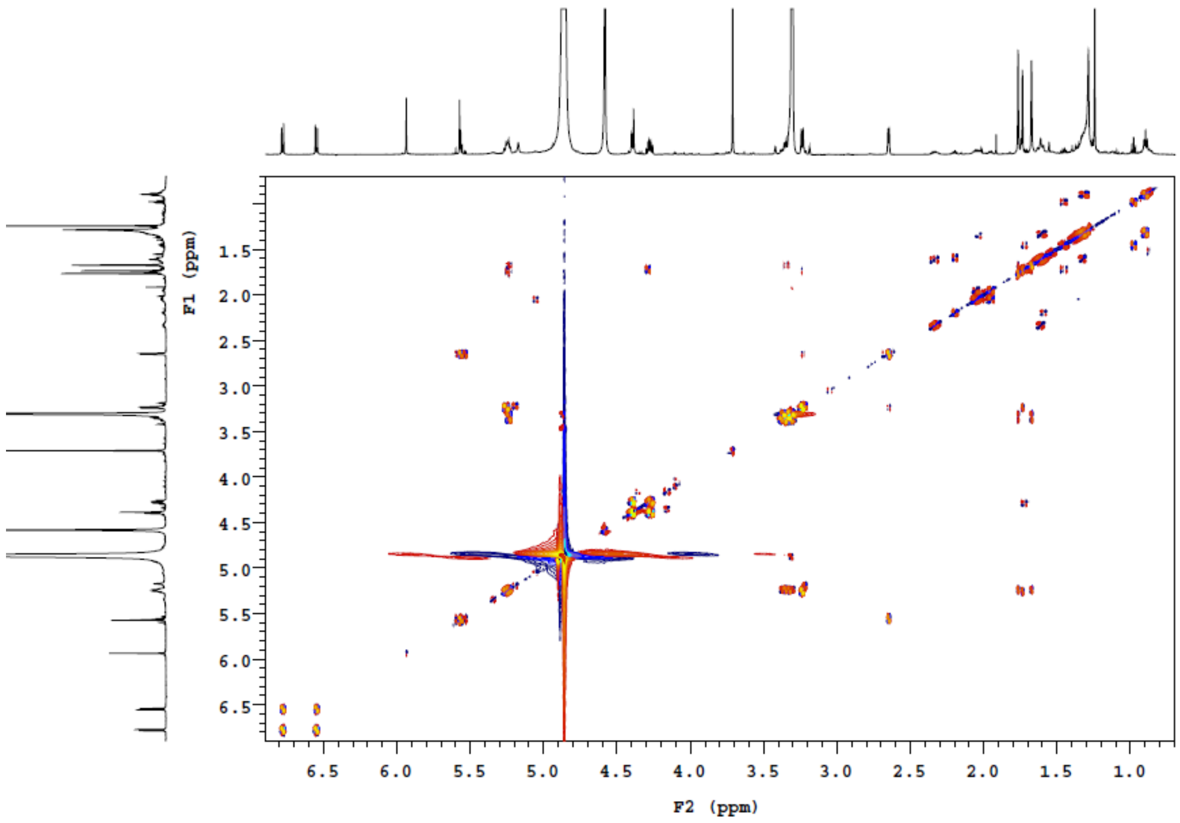

Figure S3_4: COSY spectrum (125 MHz, CD_3OD) of compound **3**

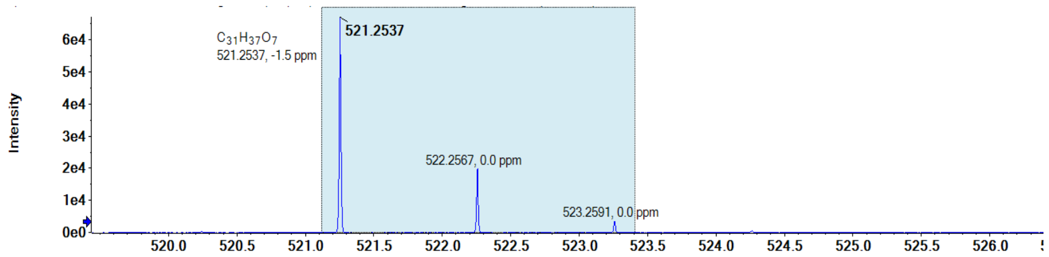

Figure S3_5: Negative ion ESI-HRMS spectrum of compound **3**

Table S3: NMR data of compound **3**

No	δ_H, *mult* (*J* in Hz)	δ_C		HMBC	COSY
2	A: 4.39, *d* (8.9)	72.0[a]	CH_2	C9, C3, C4, C1′	H2B, H3
	B: 4.38, *d* (6.8)			C9, C3, C4, C1′	H2A, H3
3	4.24, *dd* (8.9/6.8)	46.8[a]	CH	C2′, C4, C1′, C2	H2A, H2B
4		199.6[b]	C=O		
5		162.9[b]	C		
6		109.8[b]	C		
7		166.1[b]	C		
8	5.94, *s*	94.8 [a]	CH	C4, C6, C7	
9		162.9[b]	C		
10		103.9[b]	C		
1′		120.8[b]	C		
2′		159.3[b]	C		
3′		123.3[b]	C		
4′		157.7[b]	C		
5′	6.55, *d* (8.4)	111.9[a]	CH	C1′, C3′, C4′, C2′	H6′
6′	6.78, *d* (8.4)	127.8[a]	CH	C3, C3′, C4′, C2′	H5′
1″	A: 3.36, *dd* (14.8/7.1)	24.2[a]	CH_2	C3′, C2′, C4′, C2″, C3″	H2″
	B: 3.32, under solvent				
2″	5.24, *br* t	124.2[a]	CH		H1″
3″		131.4[b]	C		
4″	1.67, *s*	25.6[a]	CH_3	C5″, C2″, C3″	
5″	1.77, *s*	17.8[a]	CH_3	C4″, C2″, C3″	
1‴	3.24, *d* (7.3)	21.7[a]	CH_2	C6, C2‴, C3‴, C7, C5	H2‴
2‴	5.25, *br t*	124.2[a]	CH		H1‴
3‴		134.5[b]	C		
4‴	2.65, *br d* (4.5)	43.4[a]	CH_2	C9‴, C2‴, C6‴, C7‴	H5‴
5‴	5.57, *m*	126.0[a]	CH	C4‴,	H4‴
6‴	5.57, *m*	139.8[a]	CH	C4‴, C7‴, C8‴/10‴	
7‴		71.3[b]	C		
8‴	1.24, *s*	29.6[a]	CH_3	C10‴, C6‴, C7‴	
9‴	1.74, *s*	16.0	CH_3	C4‴	
10‴	1.24 *s*	29.6[a]	CH_3	C8‴, C6‴, C7‴	
2″-OCH_3	3.71, *s*	62.2[a]	CH_3	C2′	
5-OH	12.41, *s*				

Signals derived from [a]HSQC and [b]HMBC.

(*E*)-6-(6-Hydroxyl-3,7-dimethylocta-2,7-dienyl)-4′,5,7-trihydroxyl-2′-methoxy-3′-prenylisoflavanone (4)

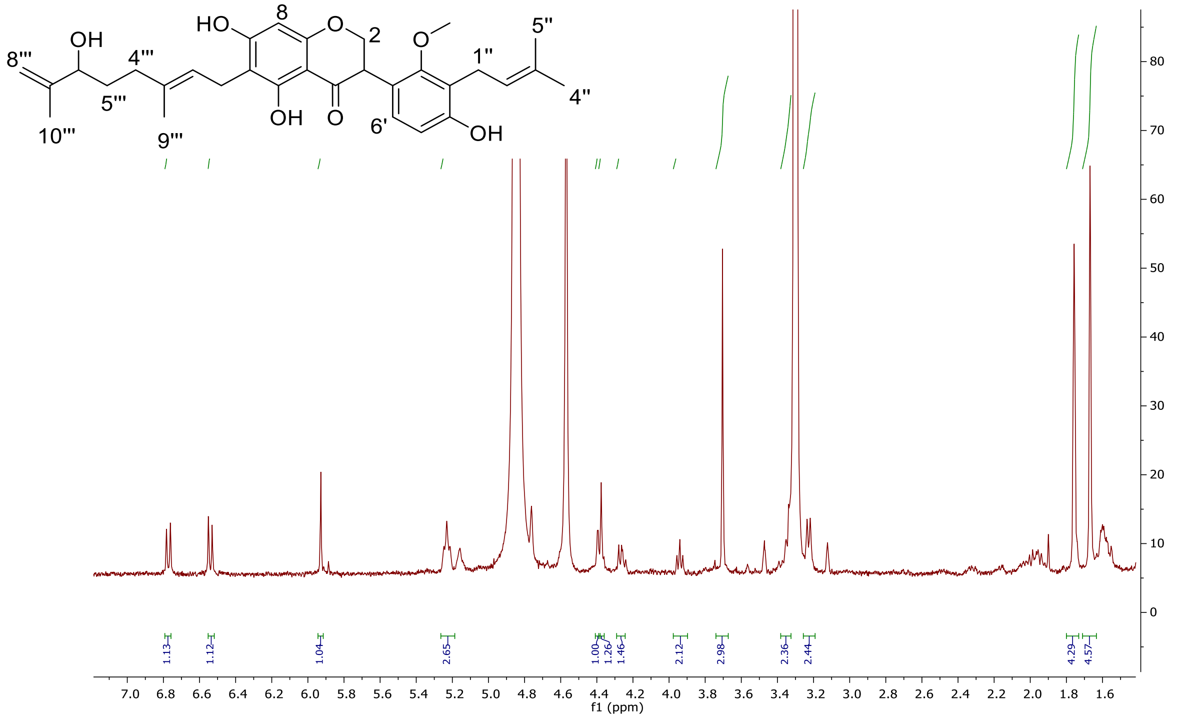

Figure S4_1: ^{1}H NMR spectrum (600 MHz, CD_3OD) of compound **4**

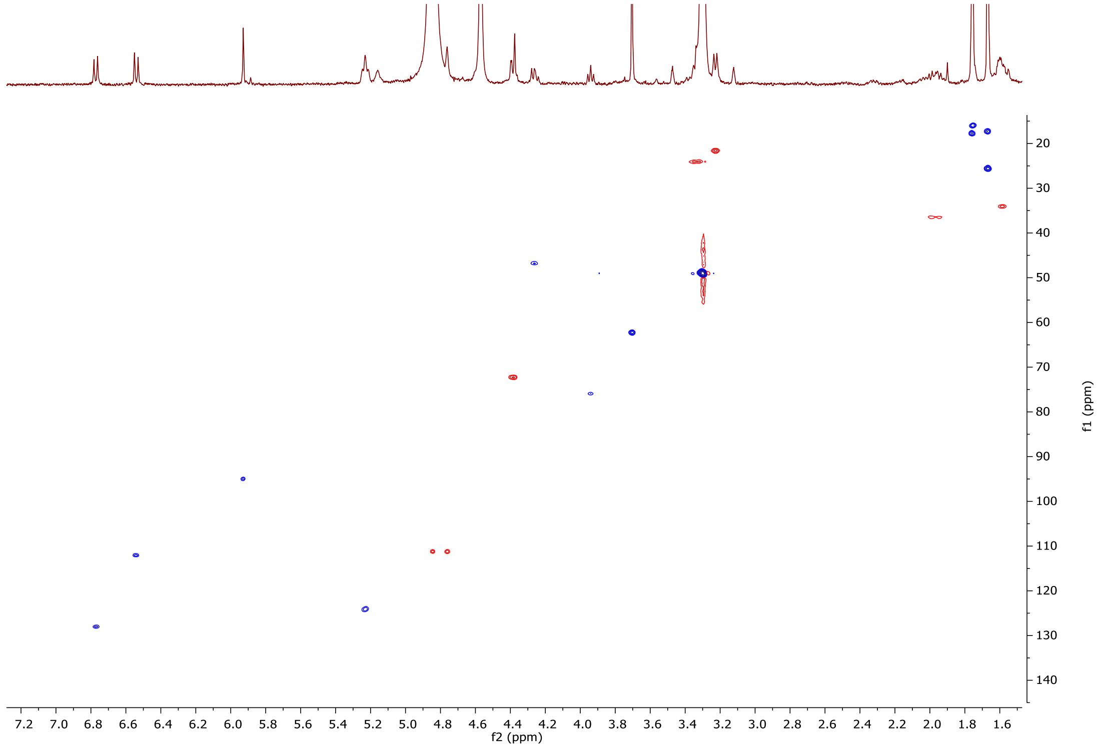

Figure S4_2: HSQCAD (NUS 50%) spectrum (600 MHz, CD_3OD) of compound **4**

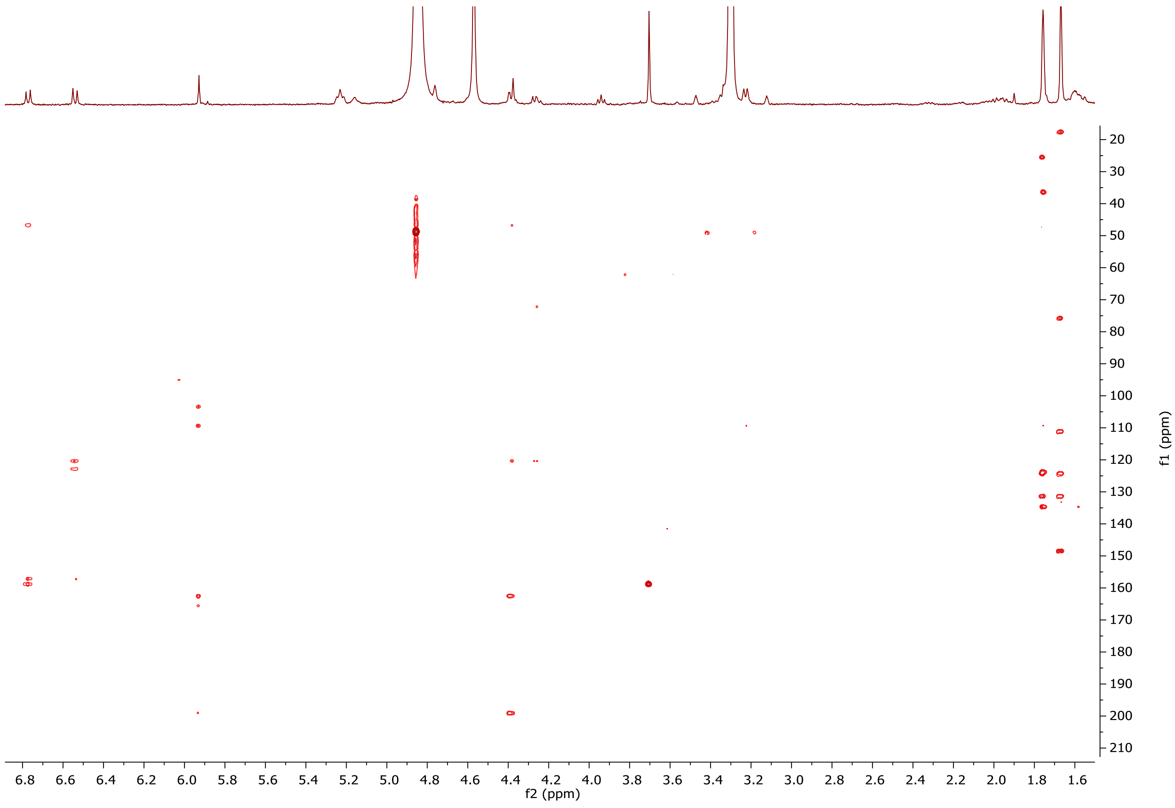

Figure S4_3: HMBC (NUS 50%) spectrum (600 MHz, CD_3OD) of compound **4**

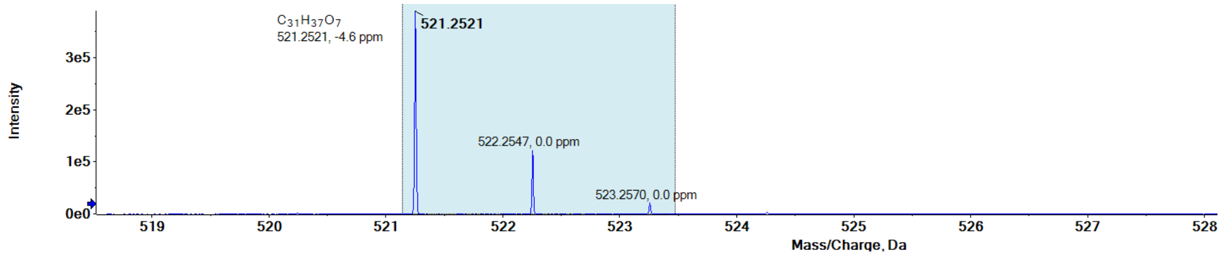

Figure S4_4: Negative ion ESI-HRMS spectrum of compound **4**

Table S4: NMR data of compound **4**

No	δ_H, *mult* (*J* in Hz)	δ_C		HMBC
2	A: 4.40, *d* (6.5)	72.2[a]	CH_2	C4, C9, C3
	B: 4.39, *d* (9.1)			C4, C9, C1′
3	4.27, *dd* (6.5/9.1)	46.8[a]	CH	C2
4		199.2[b]	C=O	
5		165.6[b]	C	
6		109.4[b]	C	
7		165.5[b]	C	
8	5.93, *s*	95.0[a]	CH	C10, C6, C7
9		162.5[b]	C	
10		103.5[b]	C	
1′		120.4[b]	C	
2′		158.8[b]	C	
3′		122.8[b]	C	
4′		157.2[b]	C	
5′	6.55, *d* (8.4)	112.1[a]	CH	C3′, C1′
6′	6.77, *d* (8.4)	128.0[a]	CH	C3, C2′, C3′
1″	3.34, *m*	24.1[a]	CH_2	
2″	5.24, *br t*	124.1[a]	CH	
3″		131.4[b]	C	
4″	1.67, *s*	25.6[a]	CH_3	C2″, C3″, C5″
5″	1.76, *s*	17.7[a]	CH_3	C3″, C2″, C4″
1‴	3.23, *br d* (6.5)	21.6[a]	CH_2	
2‴	5.24, *br t* (6.5)	124.3[a]	CH	
3‴		134.7[b]	C	
4‴	1.97, *m*	36.5[a]	CH_2	
5‴	1.59, *m*	34.1[a]	CH_2	C4‴, C3‴
6‴	3.95, *t* (6.7)	75.9[a]	CH	
7‴		148.4[b]	C	
8‴	A: 4.85, *m*	111.2[a]	$=CH_2$	
	B: 4.76, *m*			
9‴	1.77, *s*	16.0[a]	CH_3	C4‴, C3‴
10‴	1.67, *s*	17.4[a]	CH_3	C6‴, C7‴, C8‴
2′-OCH_3	3.70, *s*	62.2[a]	CH_3	C2′
5-OH	12.41, *s*			

Signals derived from [a]HSQC and [b]HMBC

6-Geranyl-4′,5,7-trihydroxyl-2′-methoxy-3′-(2,3-epoxy-3-methyl-butyl)-isoflavanone (5),
contains compound **2** as impurity

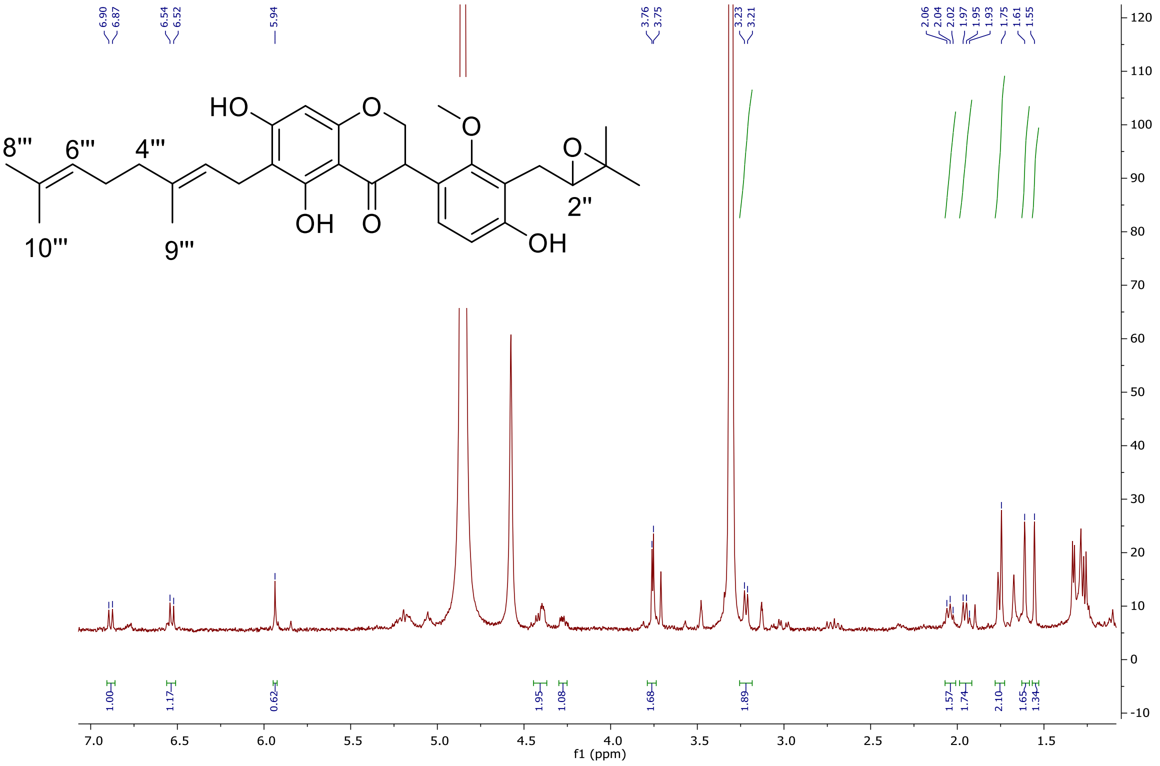

Figure S5_1:^{1}H NMR spectrum (600 MHz, CD3OD) of compound **5**

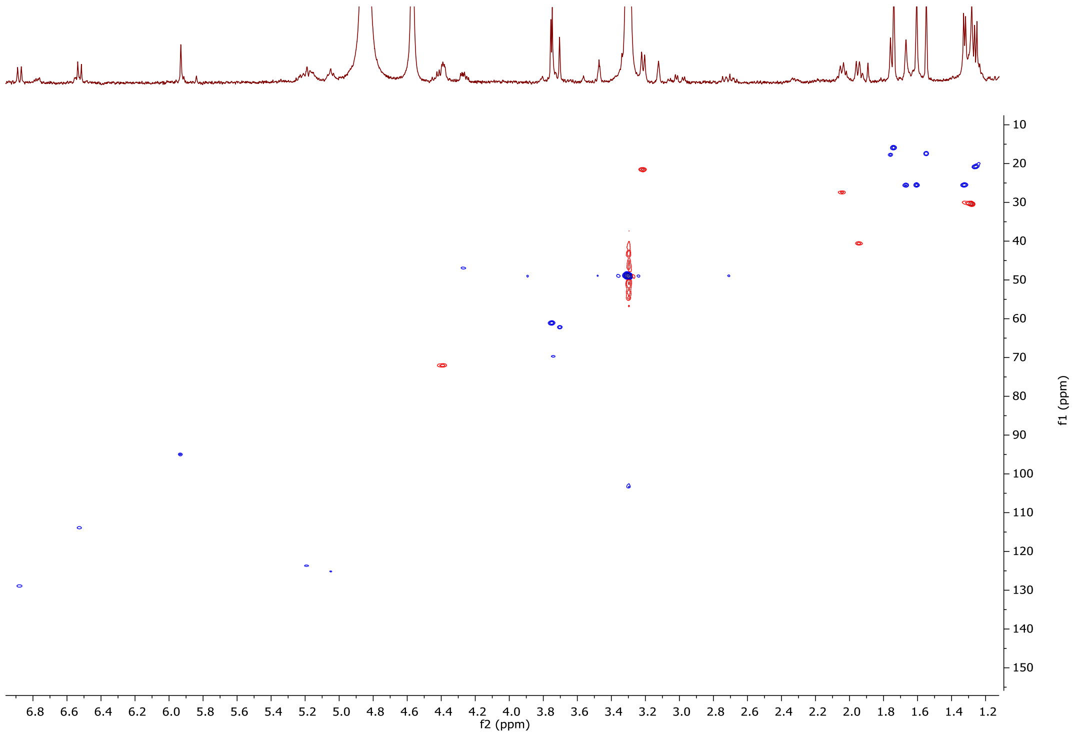

Figure S5_2: HSQCAD_ (NUS 50%) spectrum (600 MHz, CD3OD) of compound **5**

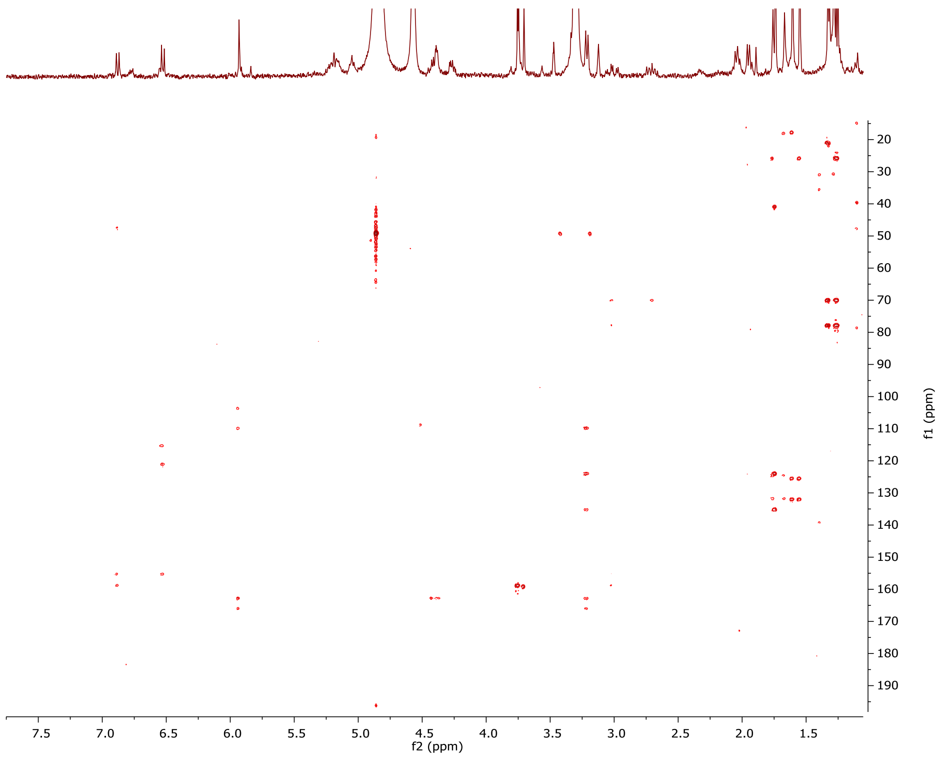

Figure S5_3: HMBC (NUS 50%) spectrum (600 MHz, CD_3OD) of compound **5**

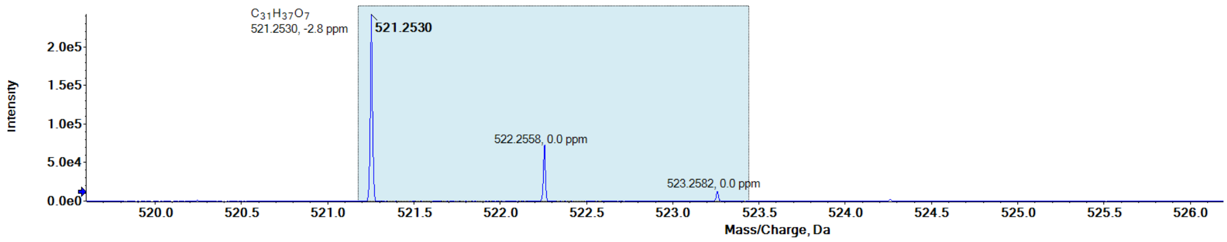

Figure S5_4: Negative ion ESI-HRMS spectrum of compound **5**

Table S5: NMR data of compound **5**

No	δ_H, *mult* (*J* in Hz)	δ_C		HMBC
2	A: 4.40, *m*	72.0 [a]	CH_2	C4, C9,
	B: 4.38, *m*			C4, C9,
3	4.27, *m*	46.9 [a]	CH	C2
4		199.1 [b]	C	
5		162.8 [b]	C	
6		109.8 [b]	C	
7		166.0 [b]	C	
8	5.94, *s*	95.3 [a]	CH	C9, C10, C6, C7, C4
9		162.8 [b]	C	
10		103.7 [b]	C	
1′		121.1 [b]	C	
2′		158.8 [b]	C	
3′		115.3 [b]	C	
4′		155.2 [b]	C	
5′	6.53, *d* (8.4)	113.9 [a]	CH	C2′, C4′, C3′
6′	6.88, *d* (8.4)	129.0 [a]	CH	C4′, C1′, C2′
1″	A: 3.02, *m*	27.6 [a]	CH_2	C2″, C3″, C2′
	B: 2.70, *m*			
2″	3.74, *m*	70.0 [a]	CH	
3″		77.8 [a]	C	
4″	1.32/1.34, s	25.6 [a]	CH_3	C2″,
5″	1.26/1.27, s	20.8 [a]	CH_3	C3″, C2″
1‴	3.22, *d* (7.2)	21.5 [a]	CH_2	C7, C5, C6, C3‴, C2‴,
2‴	5.19, *br t*	123.6 [a]	CH	
3‴		135.2 [b]	C	
4‴	1.94, *d* (7.6)	40.7 [a]	CH_2	
5‴	2.05, *m*	27.7 [a]	CH_2	
6‴	5.05, *m*	125.2 [a]	CH	
7‴		132.0	C	
8‴	1.61, *s*	25.6 [a]	CH_3	C10‴, C6‴, C7‴
9‴	1.74, *s*	16.0 [a]	CH_3	C4‴, C3‴, C2‴
10‴	1.56, *s*	17.4 [a]	CH_3	C8‴, C6‴, C7‴
2′-OCH_3	3.76/3.75, *s*	61.1[a]	CH_3	C2′

Signals derived from [a]HSQC and [b]HMBC

(*Z*)-2′,4′,5,7-Tetrahydroxyl-8-(3,7-dimethylocta-2,6-dienyl)-isoflavanone (6),
contains kenusanone H (**7**) as impurity.

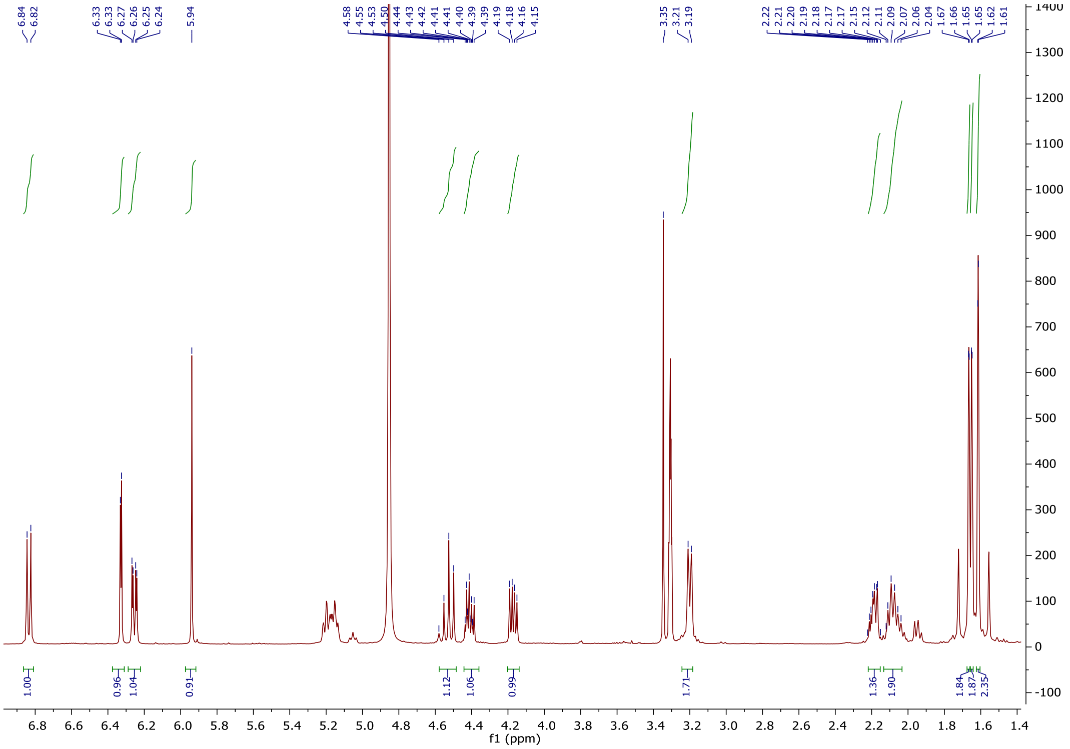

Figure S6_1: ^{1}H NMR spectrum (400 MHz, CD_3OD) of compound **6**

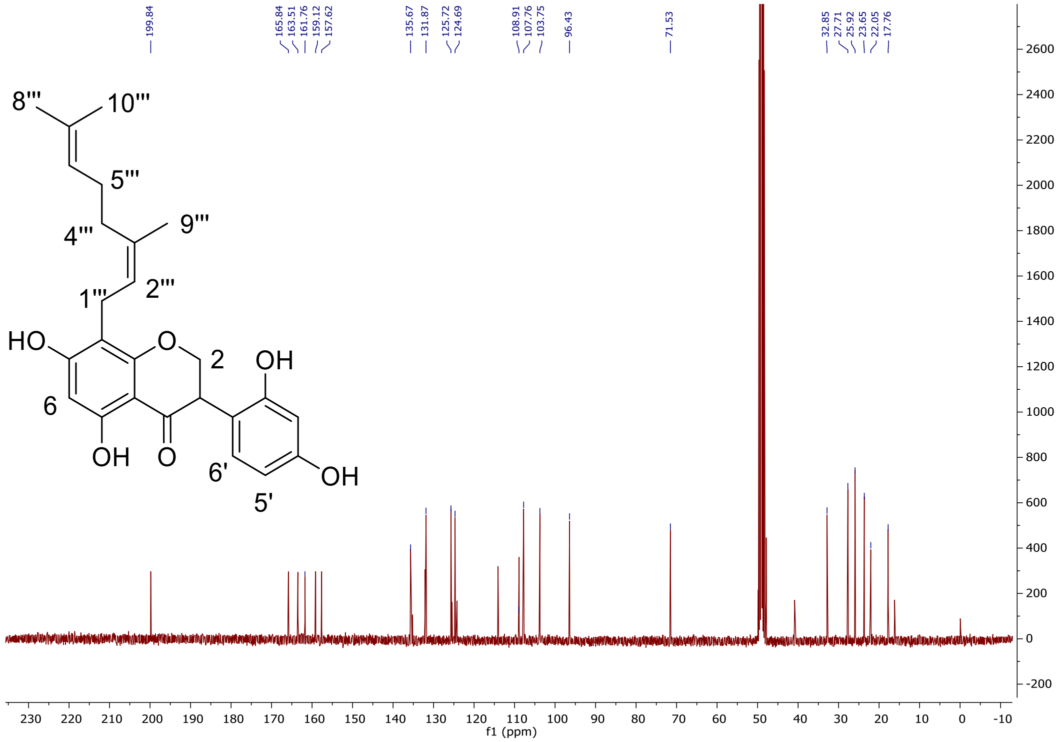

Figure S6_2:^{13}C NMR spectrum (125 MHz, CD_3OD) of compound **6**

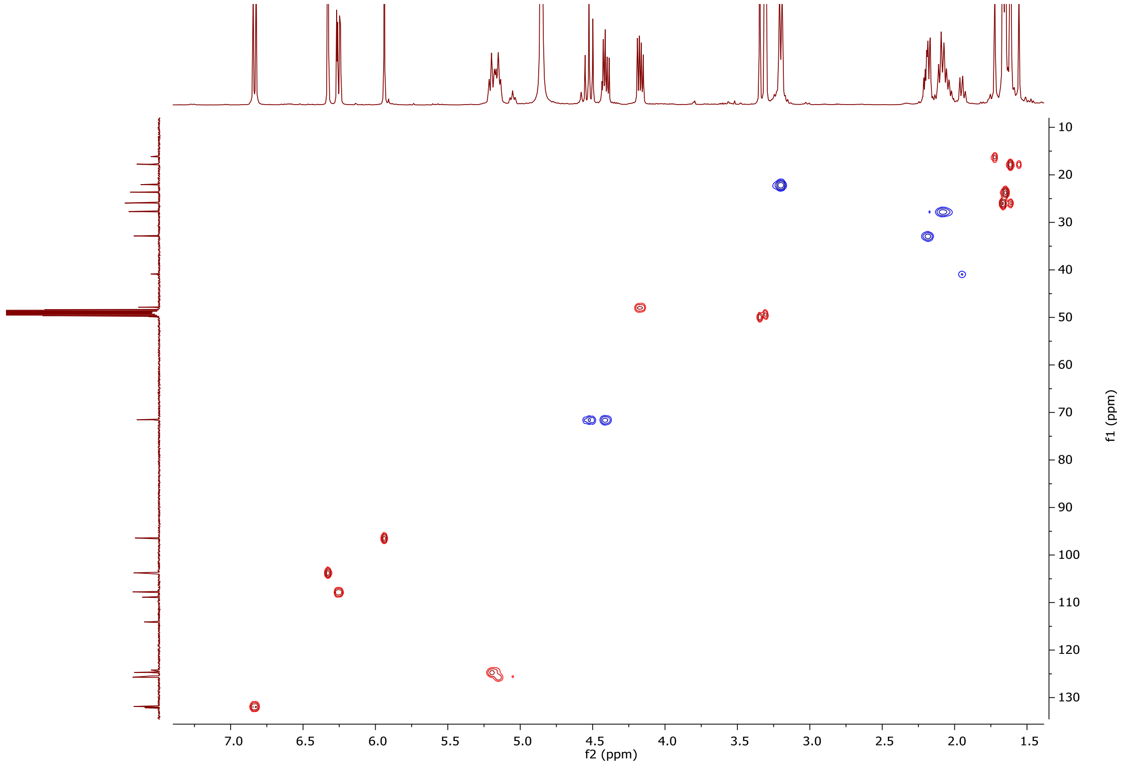

Figure S6_3: HSQCAD spectrum (125 MHz, CD_3OD) of compound **6**

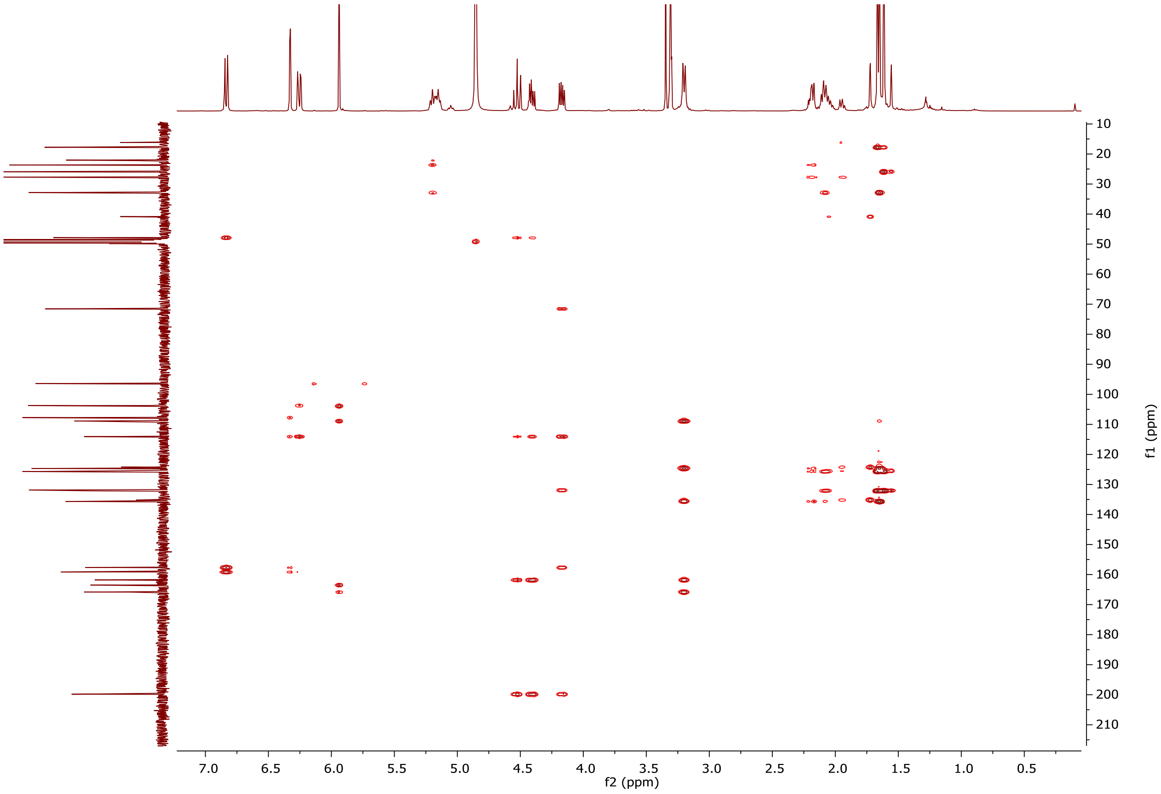

Figure S6_4: HMBC spectrum (125 MHz, CD_3OD) of compound **6**

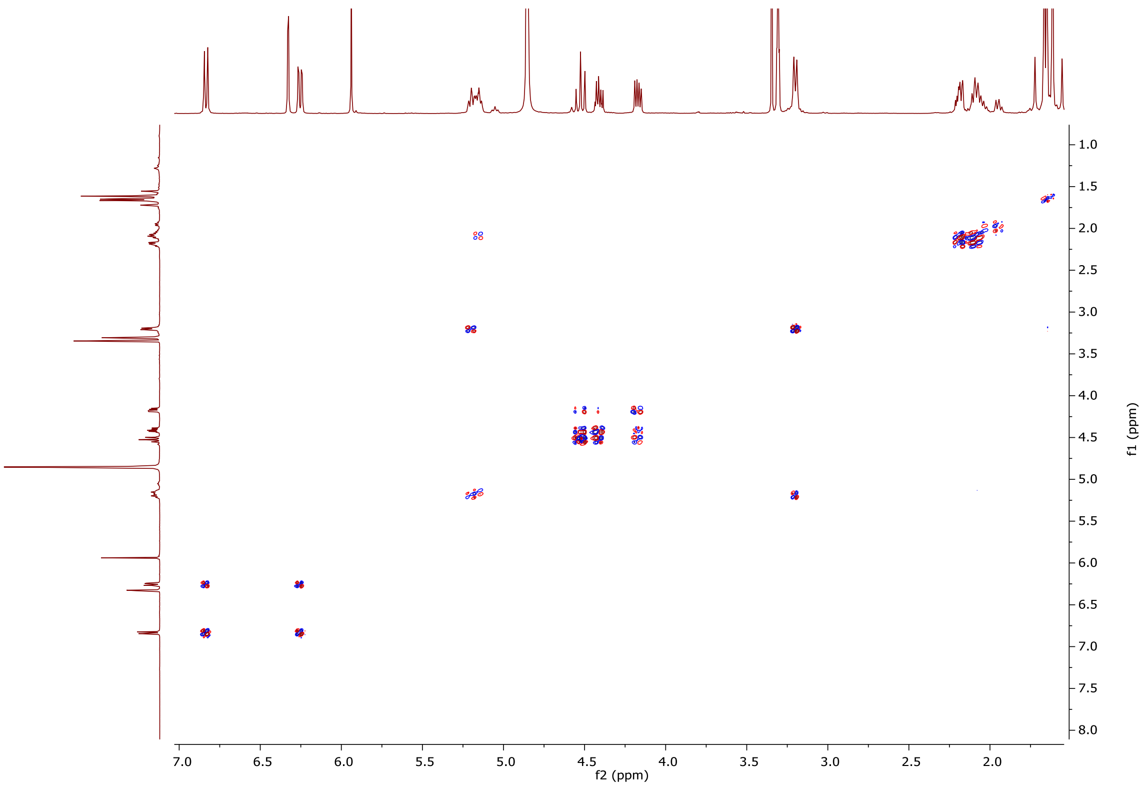

Figure S6_5: COSY spectrum (125 MHz, CD_3OD) of compound **6**

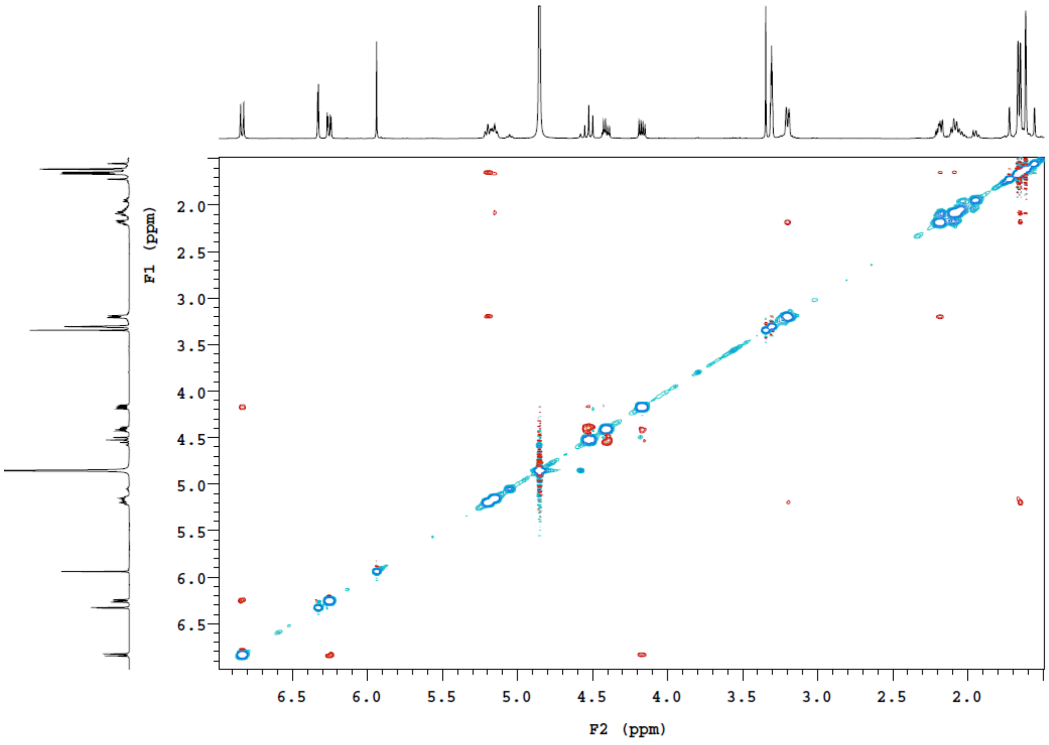

Figure S6_6: NOESY spectrum (125 MHz, CD_3OD) of compound **6**

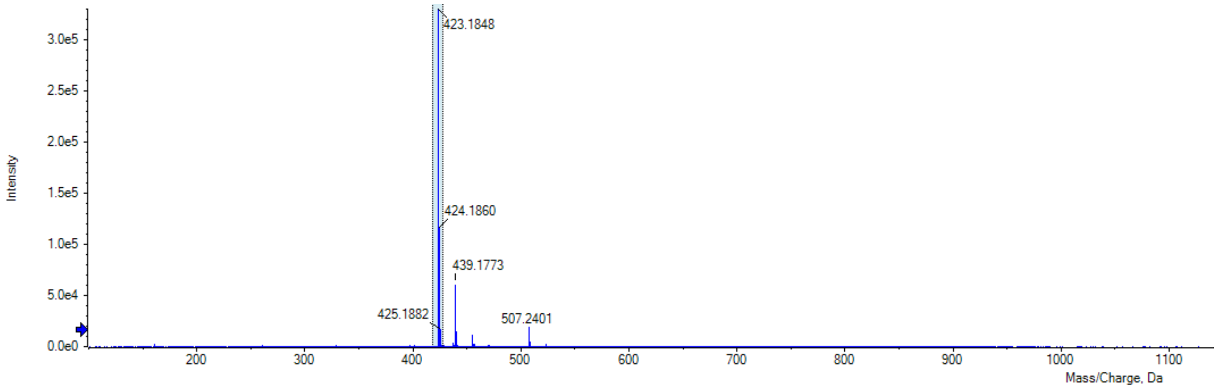

Figure S6_7: Negative ion ESI-HRMS spectrum of compound **6**

Table S6: NMR data of compound **6**

No	δ_H, *mult* (*J* in Hz)	δ_C		COSY	NOESY	HMBC
2	A: 4.53, *t* (10.8)	71.4	CH_2	H2B, H3	H2B	C4, C9, C3, C1′
	B: 4.41, *dd* (10.8, 5.5)			H2A, H3	H2A, H3	C4, C9, C3, C1′
3	4.17, *dd* (10.8, 5.5)	47.9	CH	H2A/H2B	H2A, H6′	C4, C2′, C1′, C6′
4		199.8	C=O			
5		163.5	C			
6	5.94, *s*	96.4	CH			C5, C7, C10, C8
7		165.8	C			
8		108.9	C			
9		161.8	C			
10		103.9	C			
1′		113.8	C			
2′		157.7	C			
3′	6.33, *d* (2.4)	103.8	CH			C2′, C4′
4′		159.1	C			
5′	6.26, *dd* (8.3, 2.4)	107.8	CH	H6′	H6 ′	C4′
6′	6.83, *d* (8.3)	131.9	CH	H5′	H5′, H3, H2A	C2′, C4′, C3
1‴	3.21, *br d* (7.1)	22.1	CH_2	H2‴	H 2‴, H4‴	C7, C8, C9, C2‴, C3‴
2‴	5.20, *br t* (7.1)	124.7	CH	H1‴	H 5‴	C9‴, C4‴
3‴		135.6	C			
4‴	2.18, *m*	32.9	CH_2	H5‴	H1‴, H2 ‴	C9‴, C5‴, C3‴, C2‴, C6‴
5‴	2.08, *m*	27.7	CH_2	H4‴	H6‴	C4‴, C7‴, C6‴, C3‴
6‴	5.16, *m*	125.7	CH	H5‴	H5‴, H8‴	C8‴, C10‴,
7‴		131.9	C			
8‴	1.67, *s*	25.9	CH_3		H6‴	C10‴, C6‴, C7‴
9‴	1.65, *s*	23.7	CH_3		H2‴, H5‴	C4‴, C2‴, C3‴
10‴	1.61, *s*	17.7	CH_3			C8‴, C6‴, C7‴
5-OH	12.21					

Kenusanone H (7), contains compound **6** as impurity

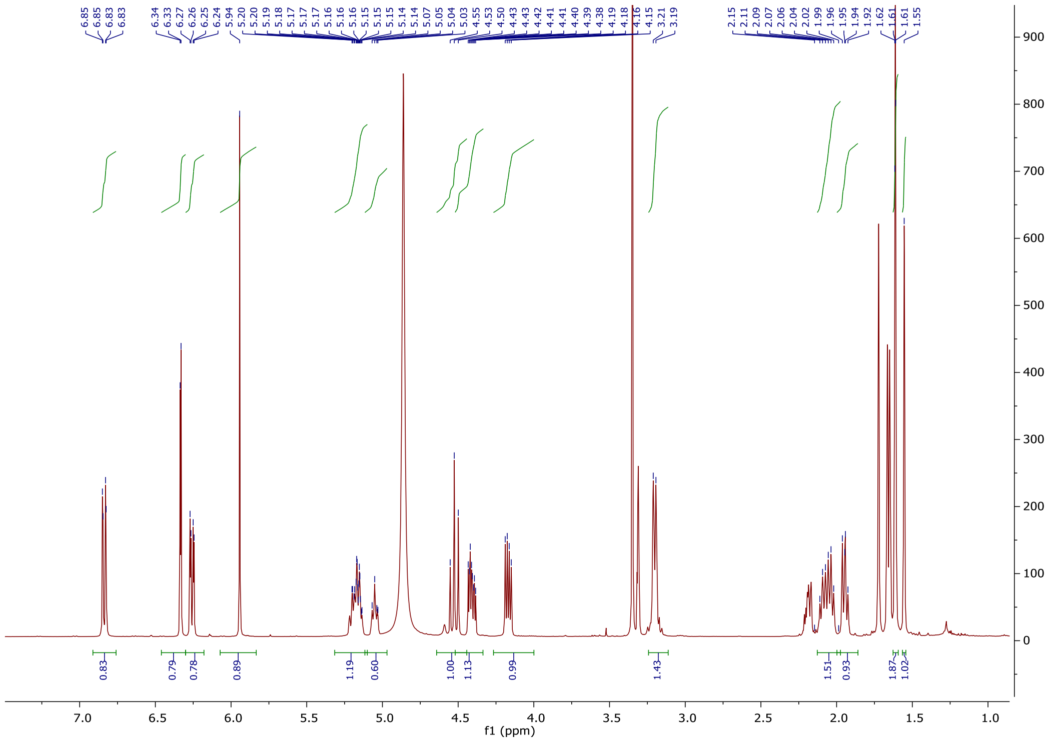

Figure S7_1: ^{1}H NMR spectrum (400 MHz, CD_3OD) of kenusanone H (**7**)

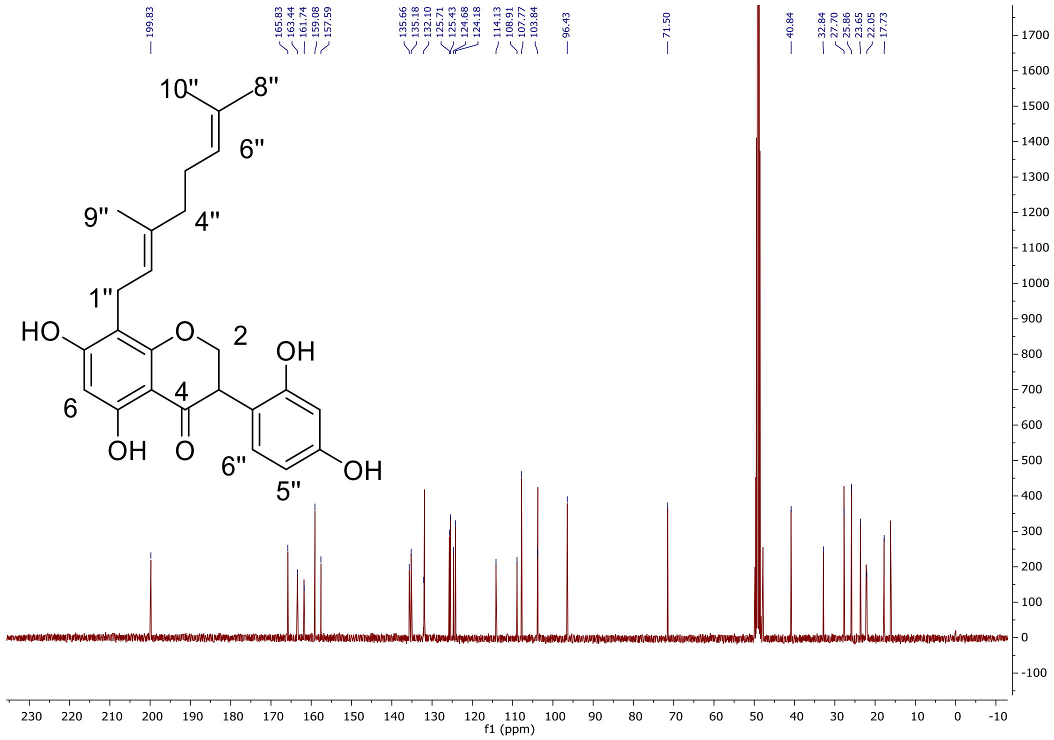

Figure S7_2: ^{13}C NMR spectrum (125 MHz, CD_3OD) of kenusanone H (**7**)

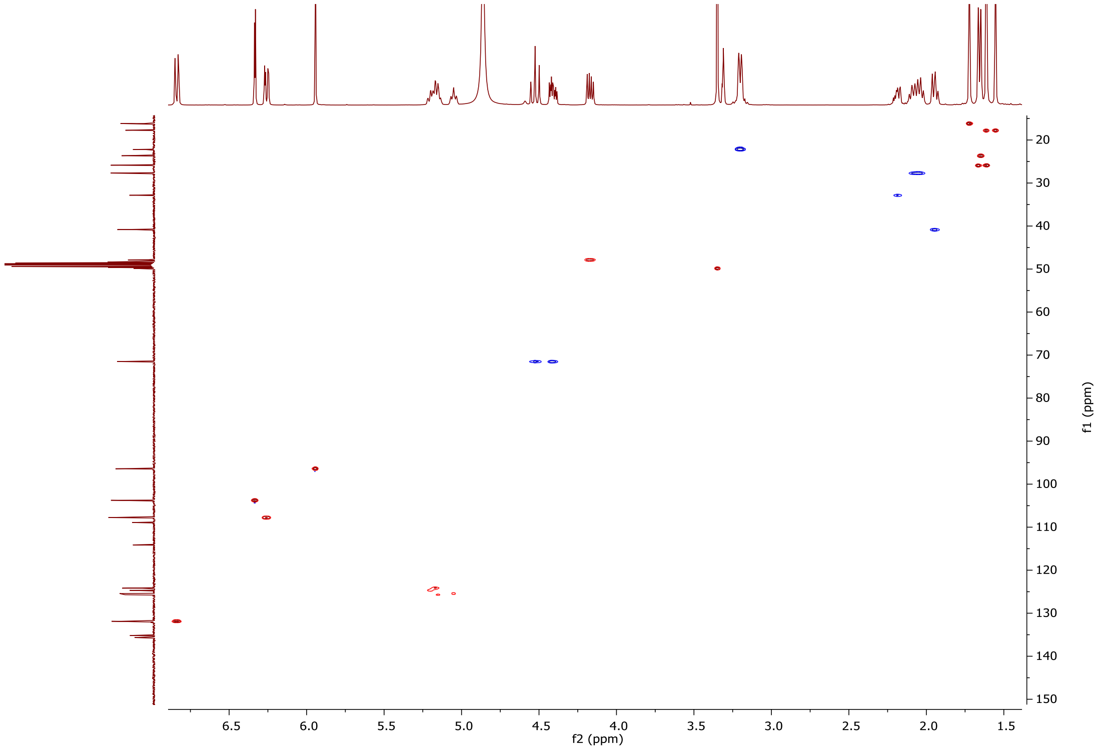

Figure S7_3: HSQCAD spectrum (125 MHz, CD3OD) of kenusanone H (**7**)

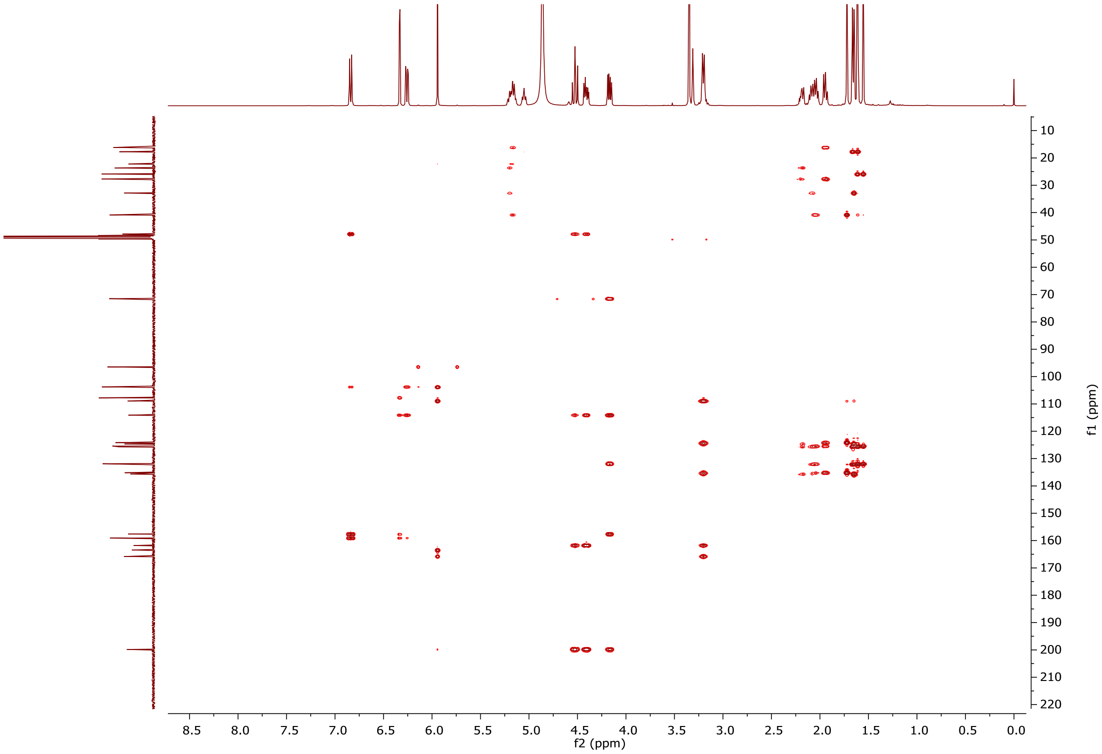

Figure S7_4: HMBC spectrum (125 MHz, CD_3OD) of kenusanone H (**7**)

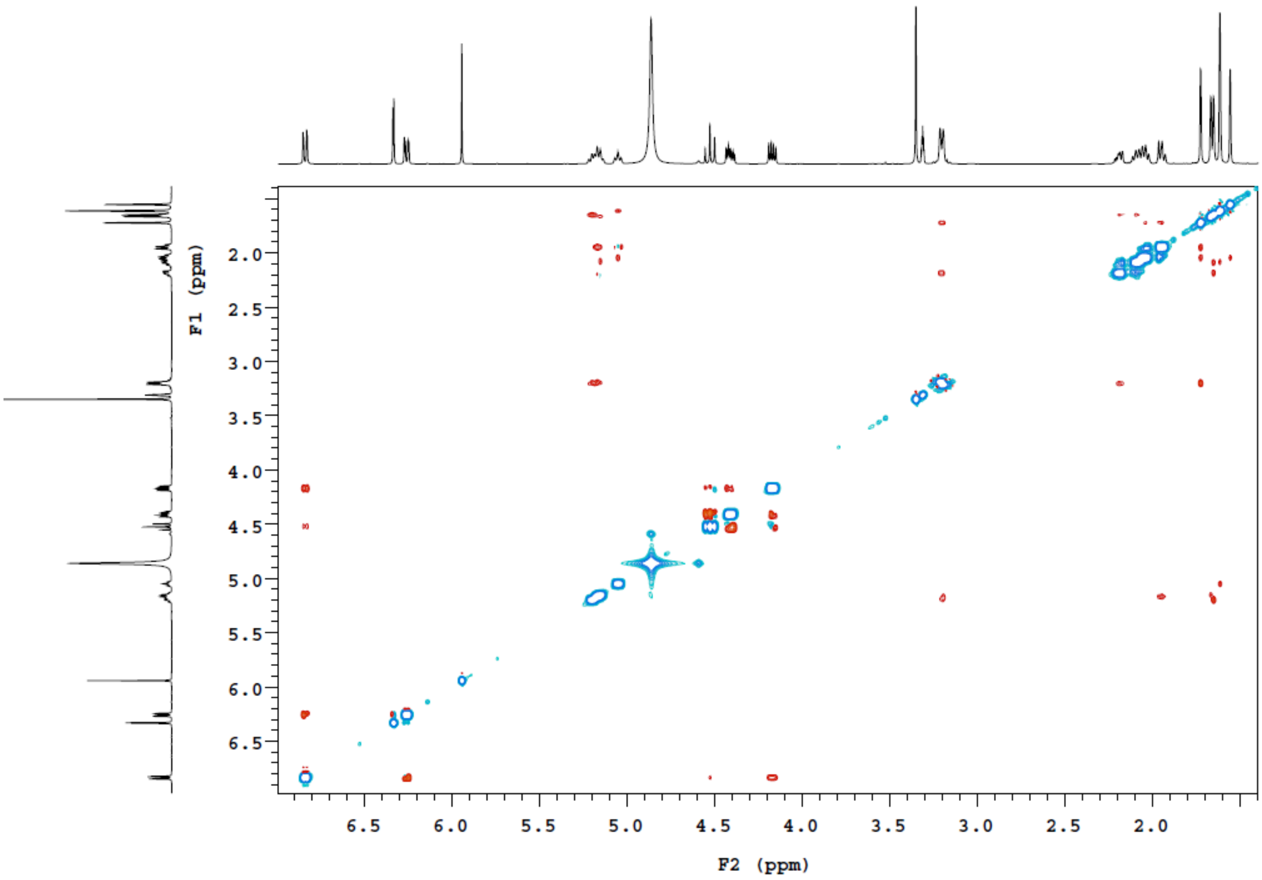

Figure S7_5: NOESY spectrum (125 MHz, CD_3OD) of kenusanone H (**7**)

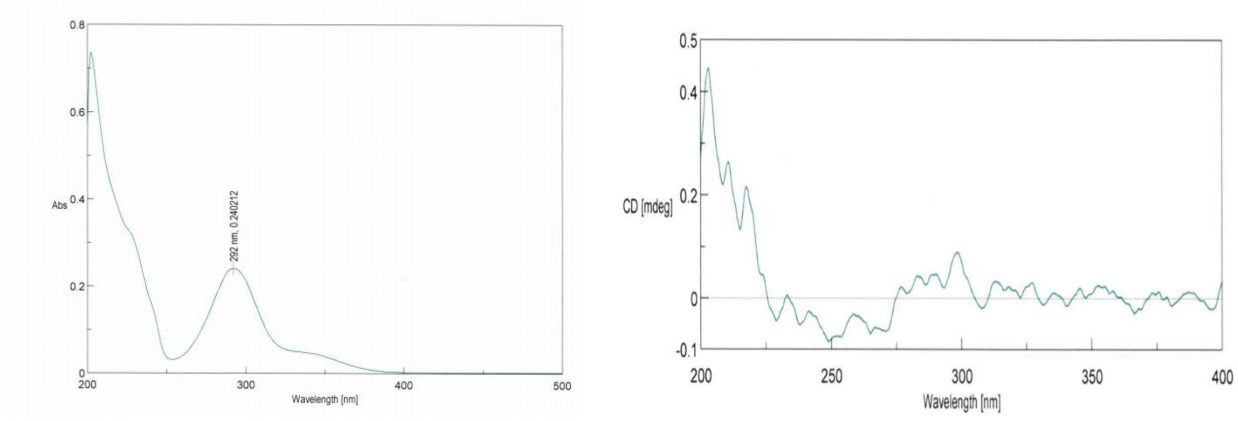

Figure S7_6: UV and CD data of kenusanone H (**7**) racemate

Table S7: NMR data of kenusanone H (**7**)

No	δ_H, *mult* (*J* in Hz)	δ_C	COSY	NOSY	HMBC
2	A: 4.53, *t* (10.7)	71.5	H2B, H3	H2B, H6′	C4, C1′
	B: 4.41, *dd* (10.9, 5.4)		H2A, H3	H2A, H3	C4, C1′
3	4.17, *dd* (10.5, 5.4)	47.9	H2A, H2	H2A, H6′	C4, C2′, C2
4		199.8			
5		163.5			
6	5.94, *s*	96.4			C4, C5, C7, C10
7		165.8			
8		108.9			
9		161.8			
10		103.8			
1′		114.1			
2′		157.6			
3′	6.33, *d* (2.4)	103.8			C2′, C4′, C1′, C5′
4′		159.1			
5′	6.26, *dd* (8.3, 2.4)	107.8	H6′	H6′	C1′, C3′
6′	6.84, *d* (8.3)	131.9	H5′	H5′, H3, H2A	C3, C2′, C4′
1‴	3.20, *d* (7.1)	22.2	H2‴	H9‴, H2‴	C9, C8, C7
2‴	5.17, *m*	124.2	H1‴	H1‴	C4‴, C9‴
3‴		135.2			
4‴	1.94, *m*	40.8	H5‴		C9‴, C5‴, C2‴, C3‴, C6‴
5‴	2.05, *m*	27.6	H6‴, H4‴		C7‴, C 4‴, C8‴
6‴	5.05, *m*	125.4	H5‴		C 8‴, C10‴
7‴		132.1			
8‴	1.61, *s*	25.9			C7‴, C6‴′
9‴	1.72, *s*	16.2		H1‴	C4‴, C2‴, C3‴
10‴	1.56, *s*	17.7			C7‴, C6‴, C8‴
5-OH	12.22, *s*				

(3*R*)-Kenusanone F (8), $[\alpha]_D^{25}$ -112.5 (c 0.260, MeOH)

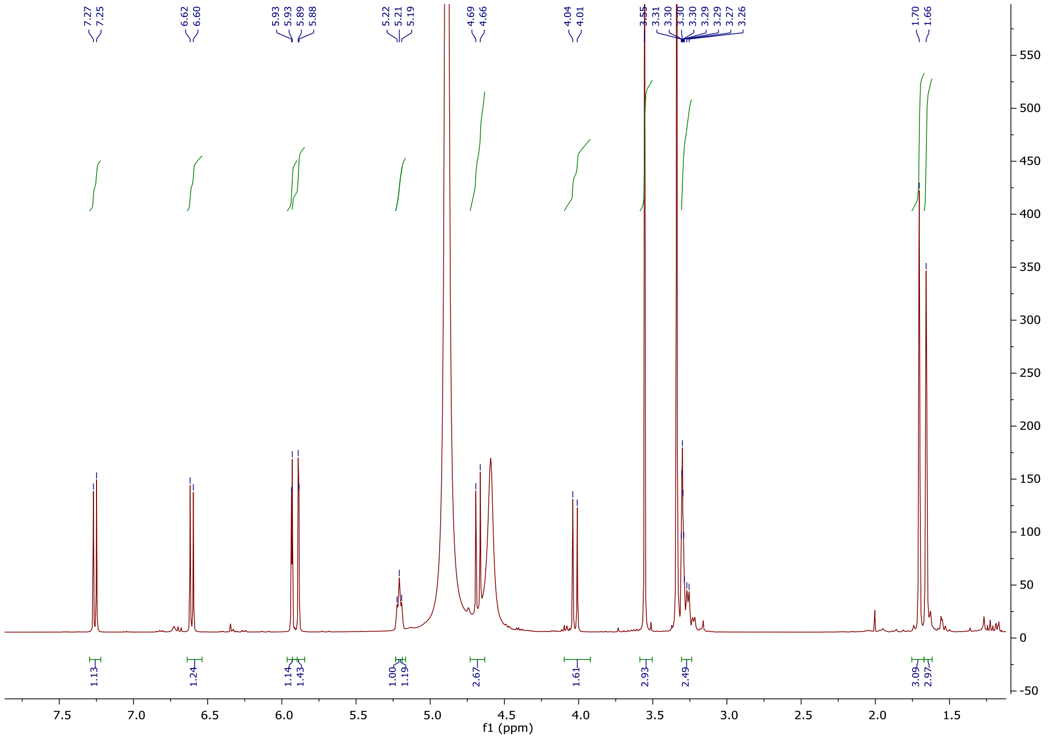

Figure S8_1: ^{1}H NMR spectrum (400 MHz, CD_3OD) of kenusanone F (**8**)

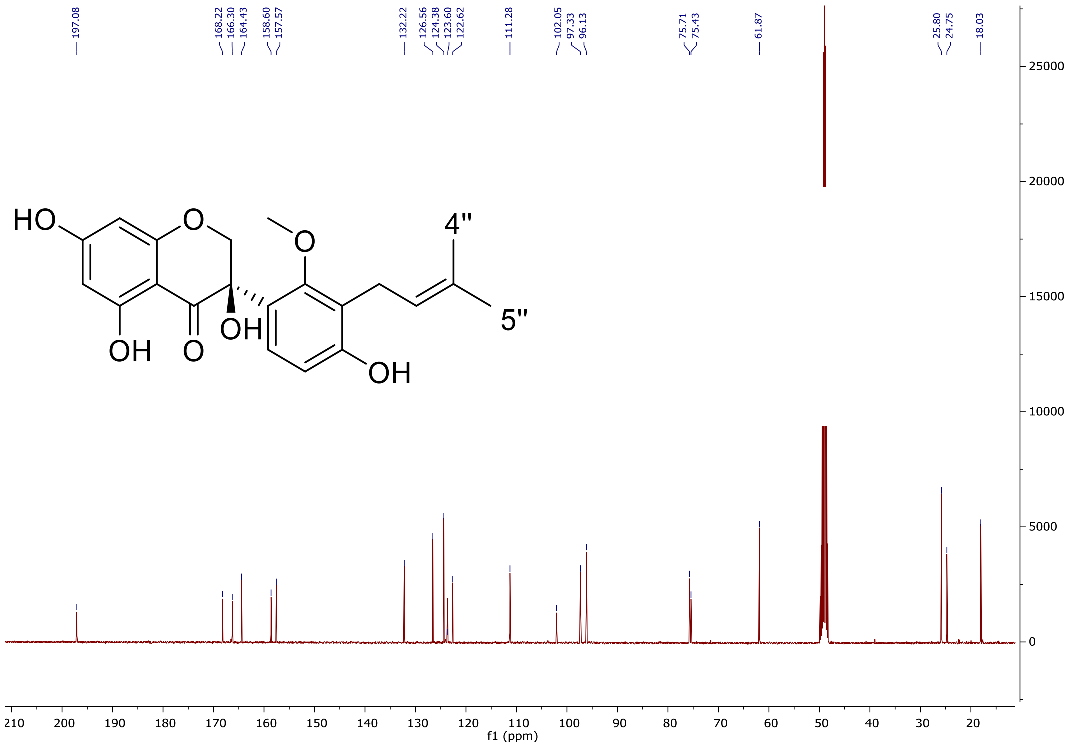

Figure S8_2: ^{13}C NMR spectrum (125 MHz, CD_3OD) of kenusanone F (**8**)

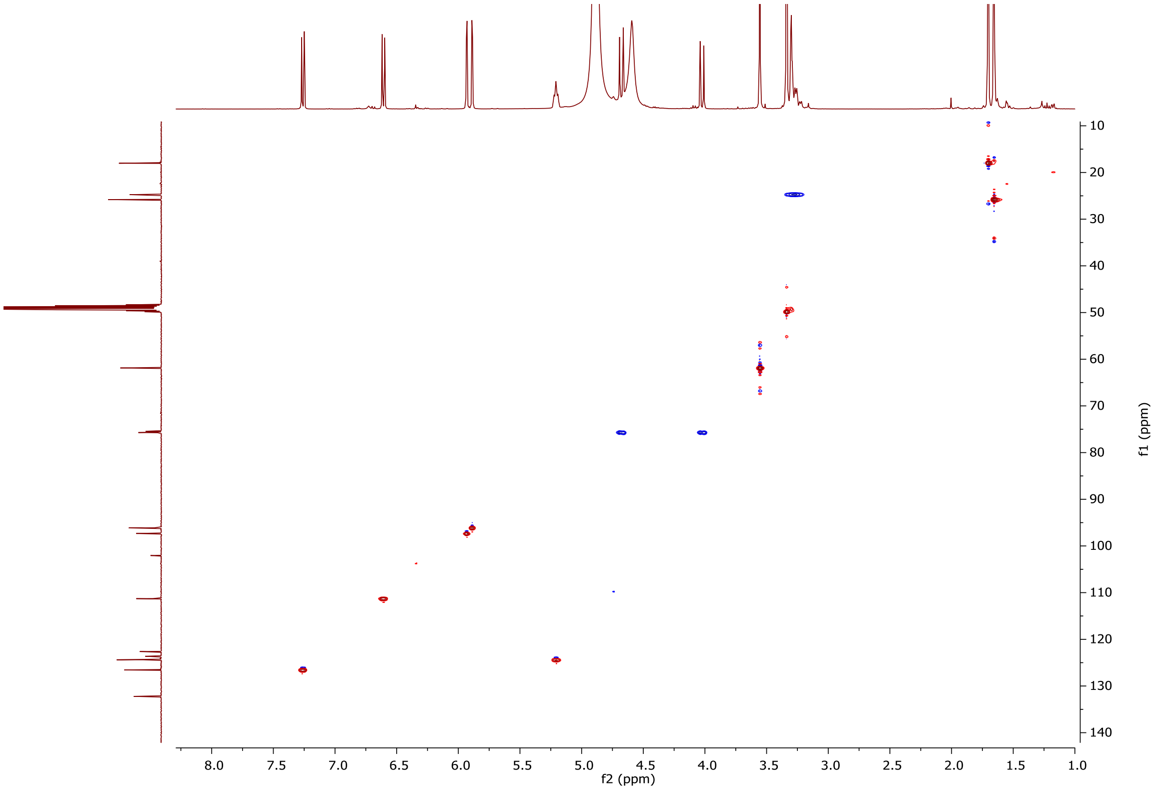

Figure S8_3: HSQCAD spectrum (125 MHz, CD_3OD) of kenusanone F (**8**)

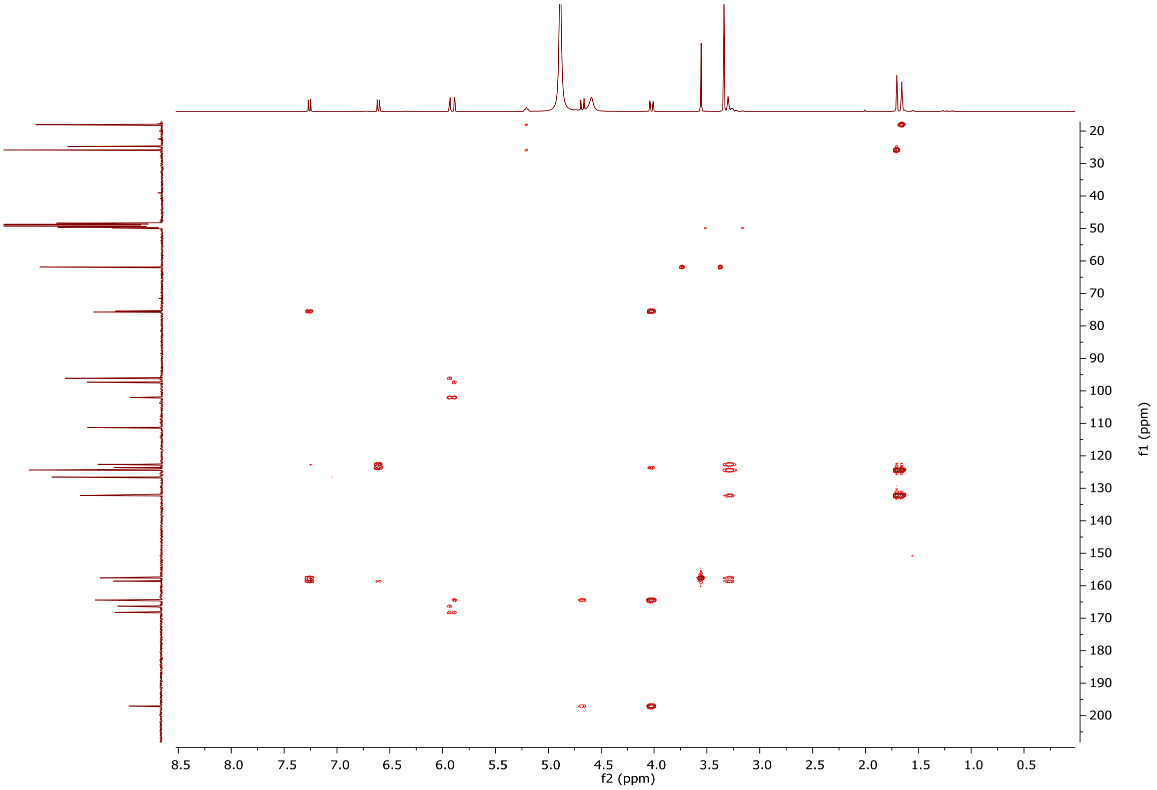

Figure S8_4: HMBC spectrum (125 MHz, CD_3OD) of kenusanone F (**8**)

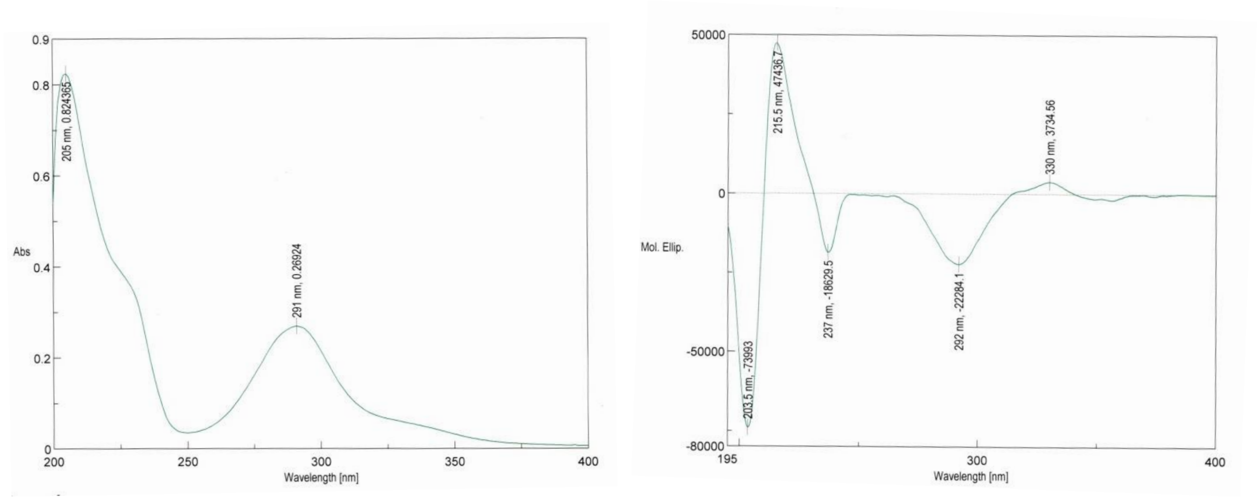

Figure S8_5: UV and CD data of (3*R*)-kenusanone F (**8**)

Table S8: NMR data of kenusanone F (**8**)

**No**	**δ_H, *mult* (*J* in Hz)**	**δ_C**	**COSY**	**HMBC**
2	A: 4.68, *d* (11.9)	75.7	H2B	C9, C4
	B: 4.02, *d*, (11.9)		H2A	C3, C1′, C9, C4
3		75.4		
4		197.1		
5		166.3		
6	5.93, *d* (2.2)	97.3	H8	C8, C10, C5, C7
7		168.2		
8	5.89, *d* (2.2)	96.1	H6	C6, C10, C9, C7
9		164.4		
10		102.0		
1′		123.6		
2′		157.6		
3′		122.6		
4′		158.6		
5′	6.61, *d* (8.5)	111.3	H6′	C3′, C1′, C4′
6′	7.26, *d* (8.5)	126.6	H5′	C3, C2′, C4′
1″	A: 3.32, *dd* (from COSY)	24.8	H2″	C3′, C2″, C3″, C2′, C4′
	B: 3.24, *dd* (15.4/6.1)			
2″	5.21, *br t* (6.6)	124.4	H1″	
3″		132.2		
4″	1.66, *s*	25.8		C5″, C2″, C3″
5″	1.70, *s*	18.1		C4″, C2″, C3″
2′-OCH_3	3.55, *s*	61.9		C2′
5-OH	12.07, *s*			

(3*R*)-Tomentosanol B (9), $[\alpha]_D^{25}$ -126.7 (c 0.300, MeOH)

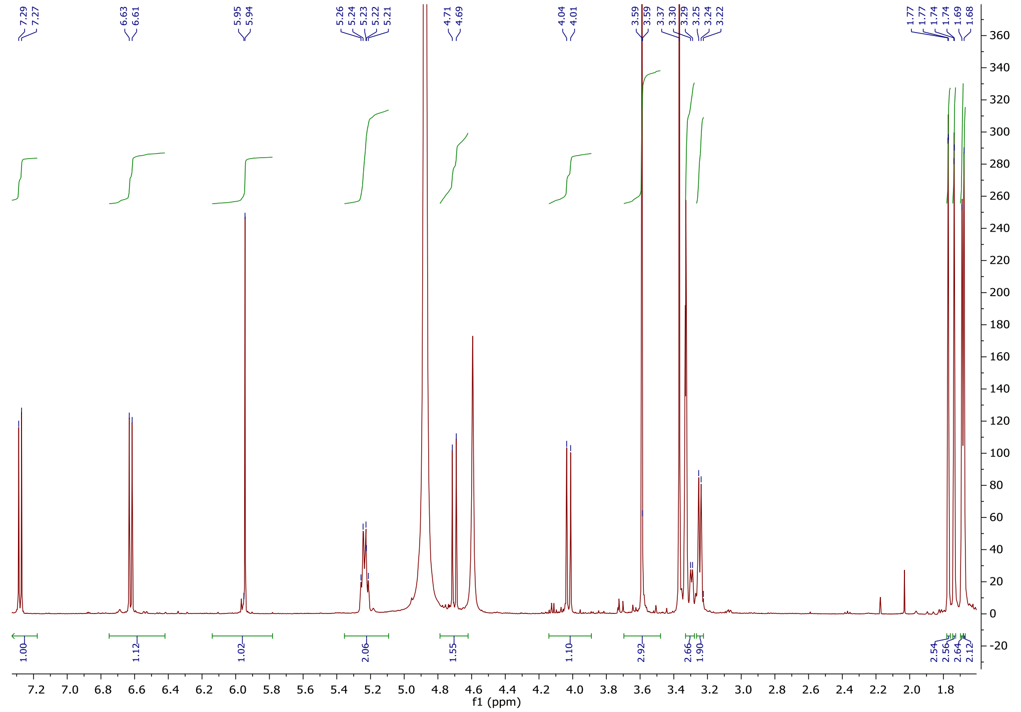

Figure S9_1: ^{1}H NMR spectrum (500 MHz, CD_3OD) of tomentosanol B (**9**)

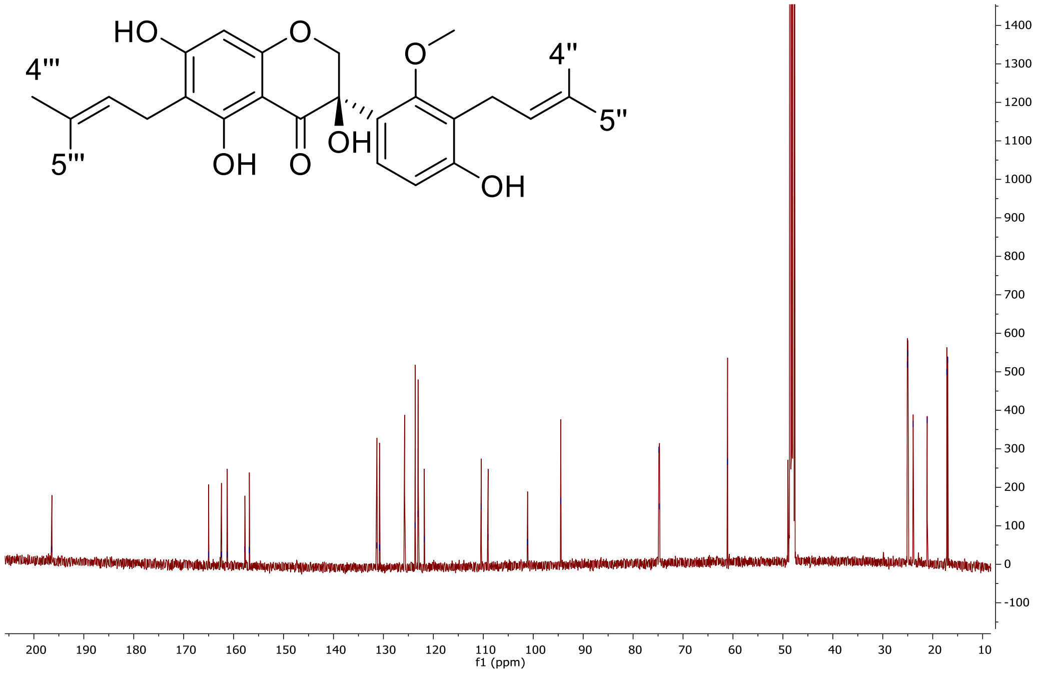

Figure S9_2: ^{13}C NMR spectrum (125 MHz, CD_3OD) of tomentosanol B (**9**)

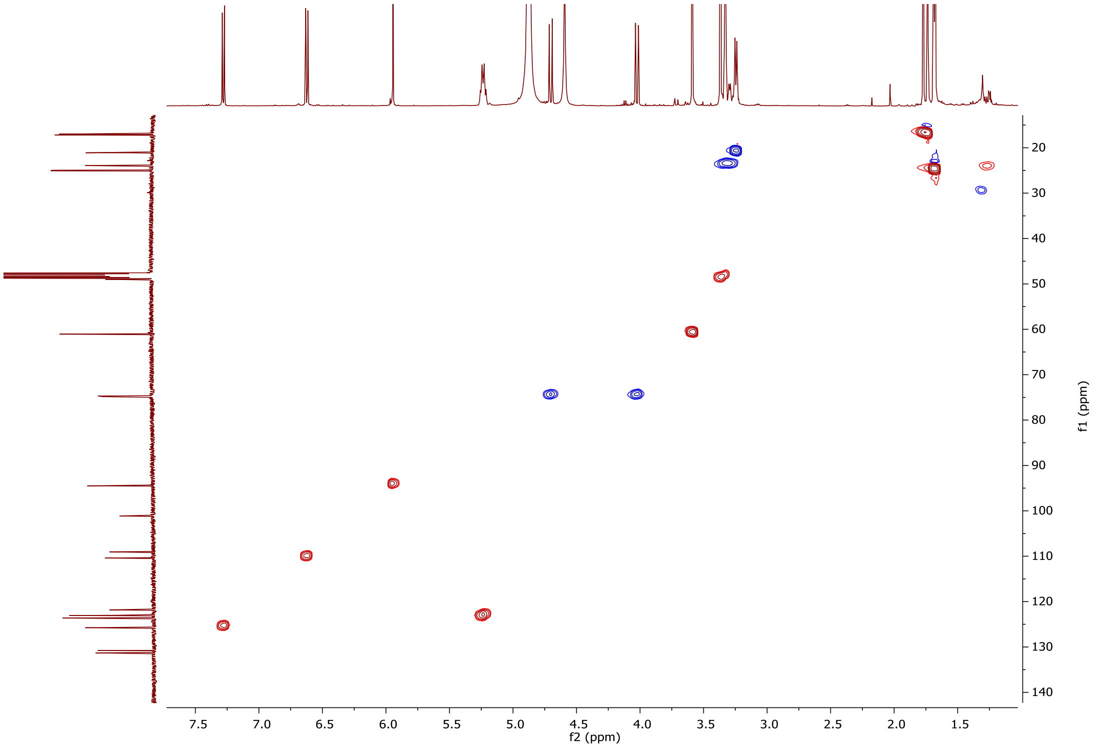

Figure S9_3: HSQCAD spectrum (125 MHz, CD_3OD) of tomentosanol B (**9**)

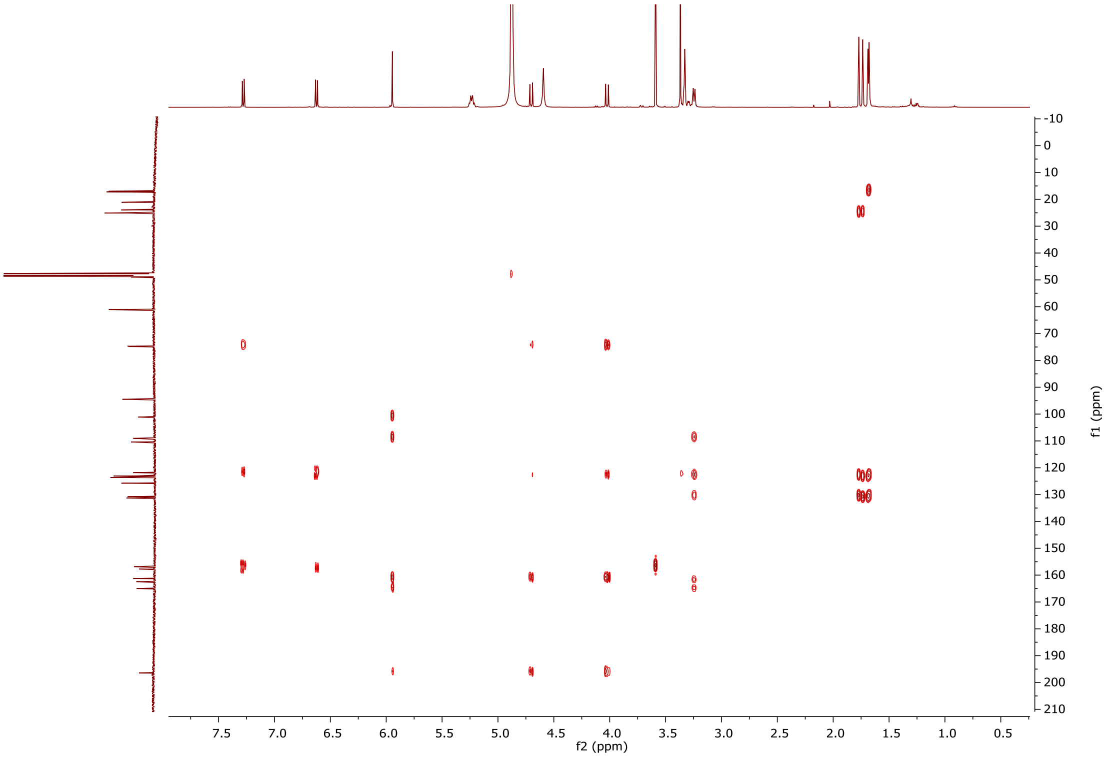

Figure S9_4: HMBC spectrum (125 MHz, CD_3OD) of tomentosanol B (**9**)

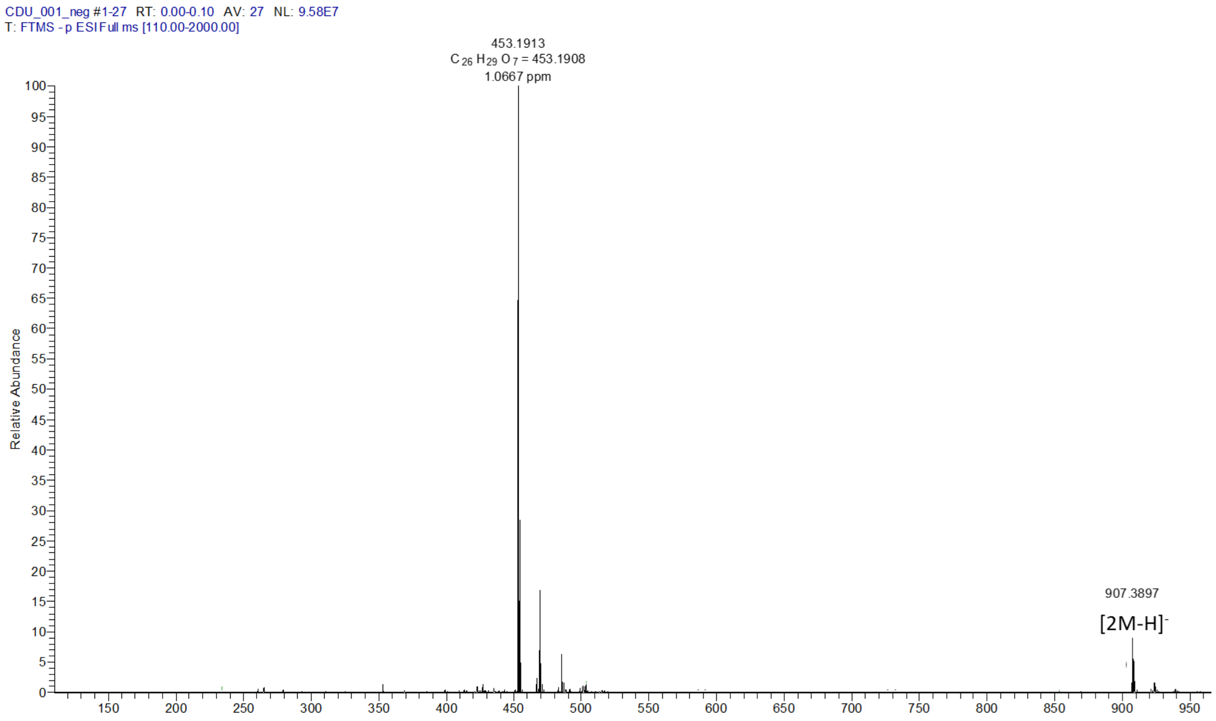

Figure S9_5: Negative ion ESI-HRMS spectrum of tomentosanol B (**9**)

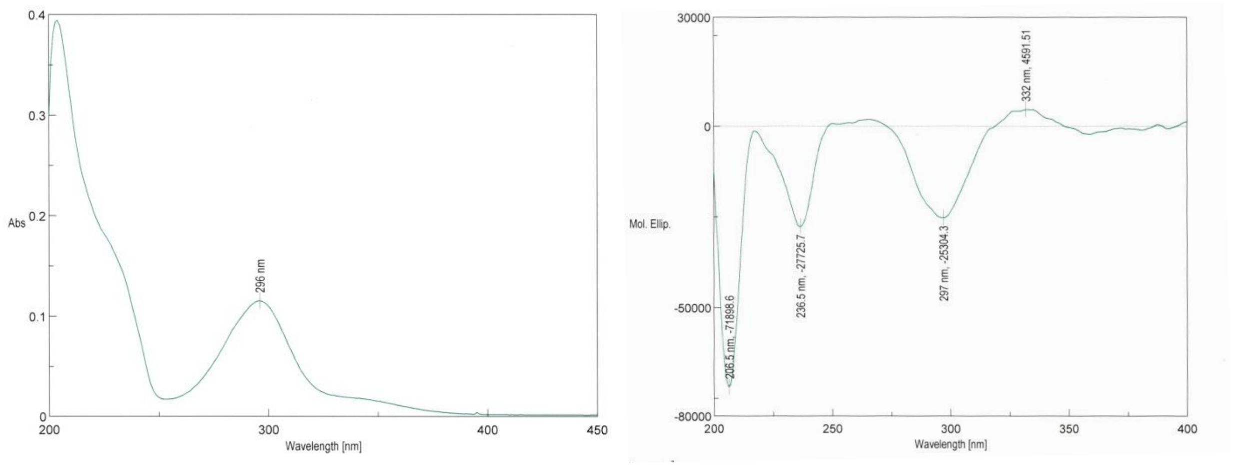

Figure S9_6: UV and CD data of (3*R*)-tomentosanol B (**9**)

Table S9: NMR of tomentosanol B (**9**)

No	δ_H,* *mult* (*J* in Hz)	δ_C		COSY	HMBC
2	A: 4.68, *d* (11.8)	75.6	CH	H2B	C9, C3, C4
	B: 4.01, *d* (11.8)		CH	H2A	C3, C4, C9, C1′
3		75.7	C		
4		197.3	C		
5		163.3	C		
6		109.9	C		
7		165.9	C		
8	5.93, *s*	95.4	CH		C6, C10, C9, C7, C4
9		162.1	C		
10		102.0	C		
1′		122.7	C		
2′		157.7	C		
3′		124.5	C		
4′		158.6	C		
5′	6.60, *d* (8.5)	111.3	CH	H6′	C1′, C6′
6′	7.26, *d* (8.5)	126.6	CH	H5′	C5′, C1′, C2′
1″	A: 3.33, m	24.8	CH_2	H2″	
	B: 3.26, dd (15.2/5.8)				
2″	5.23, *m*	124.0	CH	H1‴	
3″		132.2	C		
4″	1.67, *s*	25.9	CH_3		C2″, C3″, C5″
5″	1.75, *s*	17.9	CH_3		C2″, C3″, C4″
1‴	3.23, br *d* (7.3)	22.0	CH_2	H2‴	C5, C6, C3‴, C2‴
2‴	5.24, *m*	123.9	CH	H1‴	
3‴		131.6	C		
4‴	1.68, *s*	25.8	CH_3		C2‴, C3‴, C5‴
5‴	1.72, *s*	18.0	CH_3		C2‴, C3‴, C4‴
2′-OCH_3	3.57, *s*	61.9			C2′
5-OH	12.30, *s*				

*referenced to methanol-*d*4 solvent signal

Sophoraisoflavanone A (10)

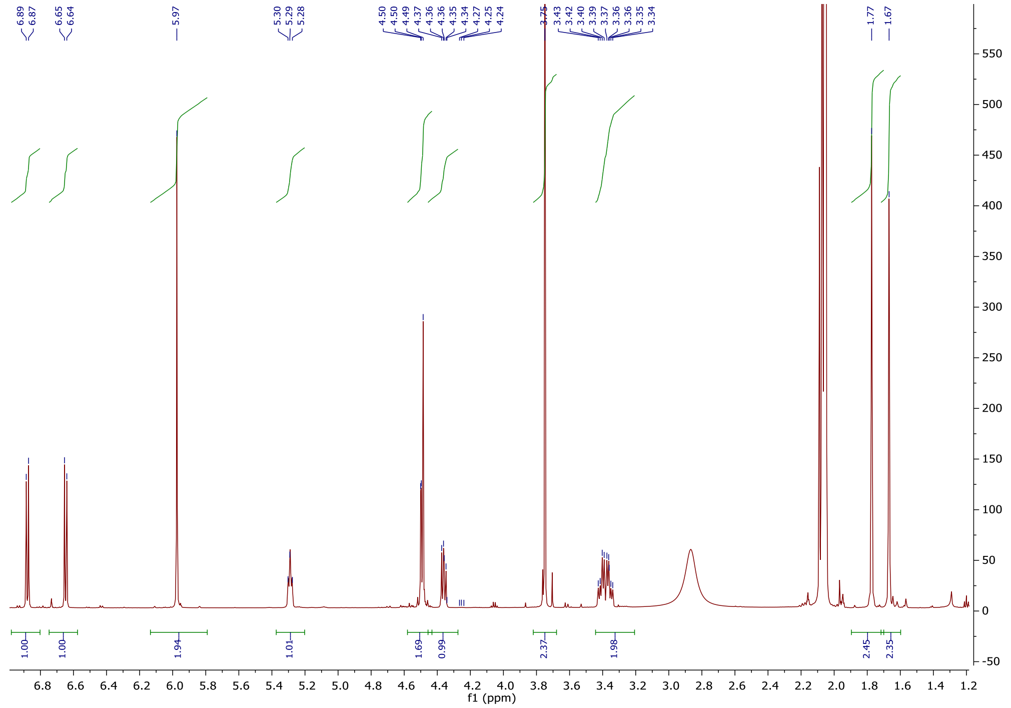

Figure S10_1: ^{1}H NMR (125 MHz, acetone-d6) of sophoraisoflavanone A (**10**)

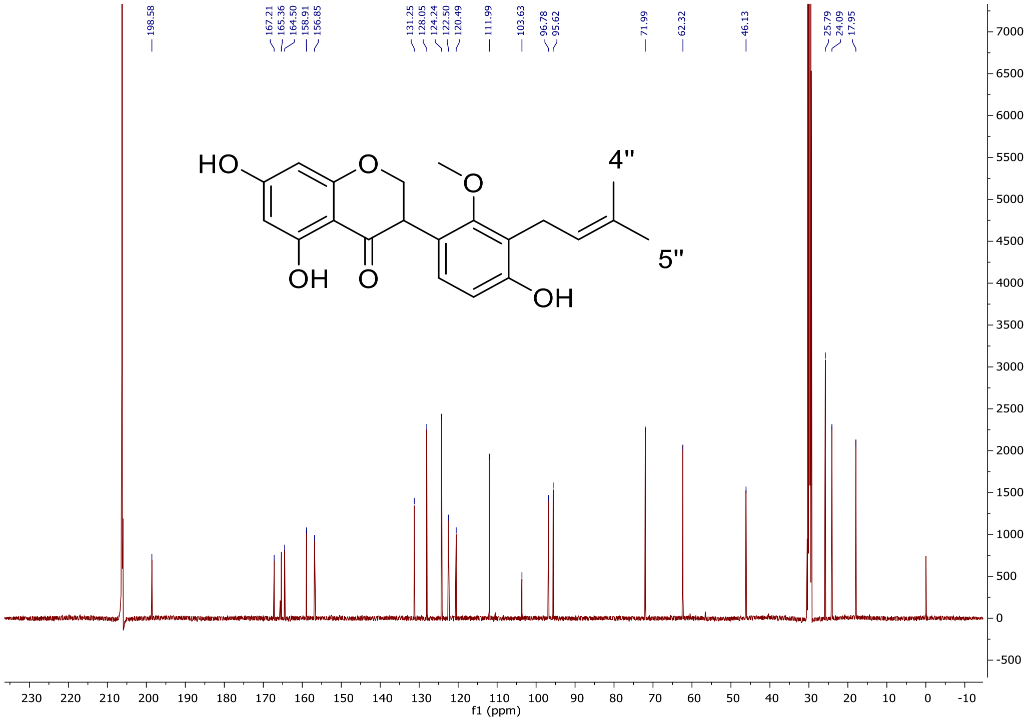

Figure S10_2: ^{13}C NMR spectrum (125 MHz, acetone-d6) of sophoraisoflavanone A (**10**)

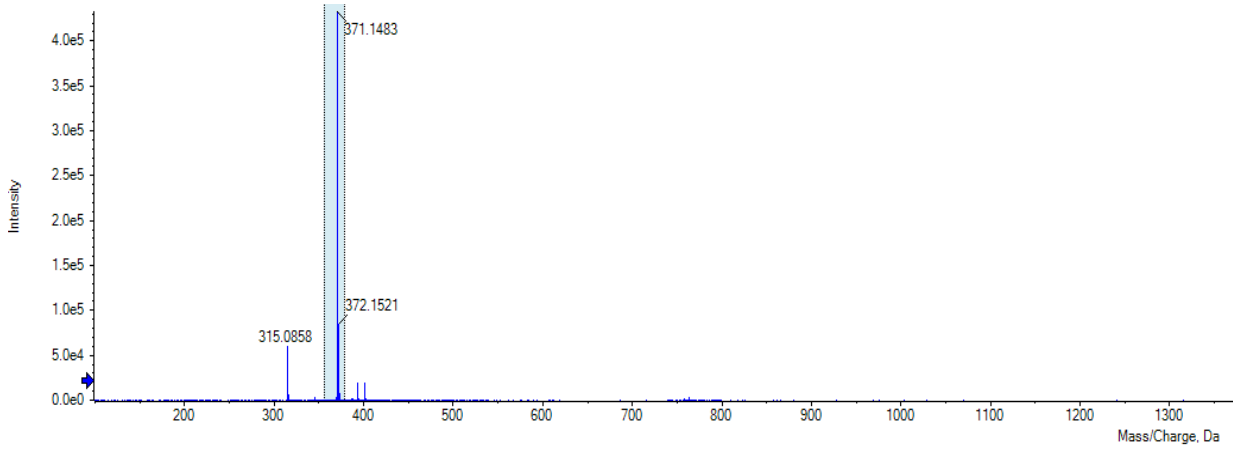

Figure S10_3: Positive ion ESI-HRMS of sophoraisoflavanone A (**10**)

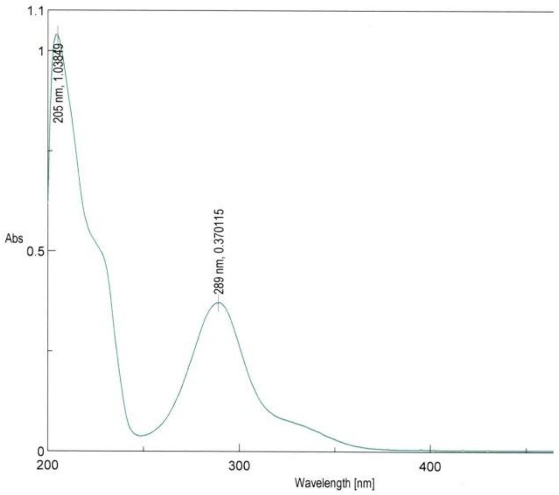

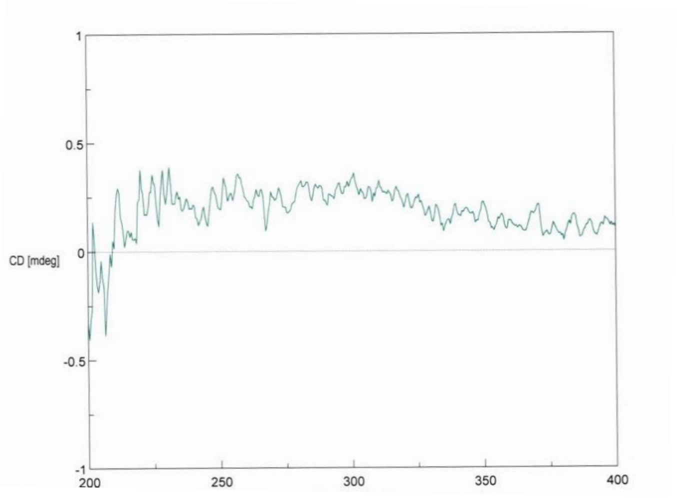

Figure S10_4: UV and CD data of sophoraisoflavanone A (**10**) racemate

Table S10: NMR data of sophoraisoflavanone A (**10**)

No	**10**		Kinoshita et al.,1990	Iinuma et al., 1992	
	δ_H, *mult* (*J* in Hz)	δ_C	δ_C	δ_H	δ_C
2	A: 4.495, *d* (8.8)<br>B: 4.492, *d* (6.8)	72.0	72.0	4.50	72.1
3	4.36, *dd* (8.9, 6.8)	46.1	46.2	4.47	46.0
4		198.6	198.2		197.8
5		165.4	165.5		165.9
6	5.97, *br s*	96.8	97.0	5.97	97.8
7		167.2	167.2		170.7
8	5.97, *br s*	95.6	95.7	5.97	96.6
9		164.5	164.3		164.4
10		103.6	103.7		103.0
1′		122.5	122.5		124.6*
2′		156.8	156.8		157.1
3′		120.5	120.5		121.0
4′		158.9	158.8		159.0
5′	6.64, *d* (8.4)	112.0	112.1	6.64	112.3
6′	6.88, *d* (8.4)	128.0	127.9	6.88	128.2
1″	A: 3.41, *dd* (14.4/6.8)<br>B: 3.35, *dd* (14.4/6.5)	24.1	24.3	3.38	24.3
2″	5.29, *br t* (6.8 Hz)	124.2	124.2	5.29	122.6*
3″		131.2	131.2		131.3
4″	1.67, br *s*	25.8	25.9	1.67	24.3
5″	1.77, br *s*	18.0	18.1	1.77	18.2
2′-OCH_3	3.75, *s*	62.3	62.4	3.75	62.5
5-OH	12.30			12.30	

*assignment may be exchanged

References

Kinoshita, T., Ichinose, K., Takahashi, C., Wu, J.-B., Sankawa, U., 1990. Chemical studies on *Sophora tomentosa*: The isolation of a new class of isoflavonoid. Chem. Pharm. Bull. 38 (10), 2756-2759. https://doi.org/10.1248/cpb.38.2756.

Iinuma, M., Ohyama, M., Tanaka, T., Mizuno, M., Soon-Keun, H., 1992. Three 2’,4’,6’-trioxygenated flavanones in roots of *Echinosophora koreensis*. Phytochemistry 31 (2), 665–669. https://doi.org/10.1016/0031-9422(92)90056-V.

Methyl dalbergin (11)

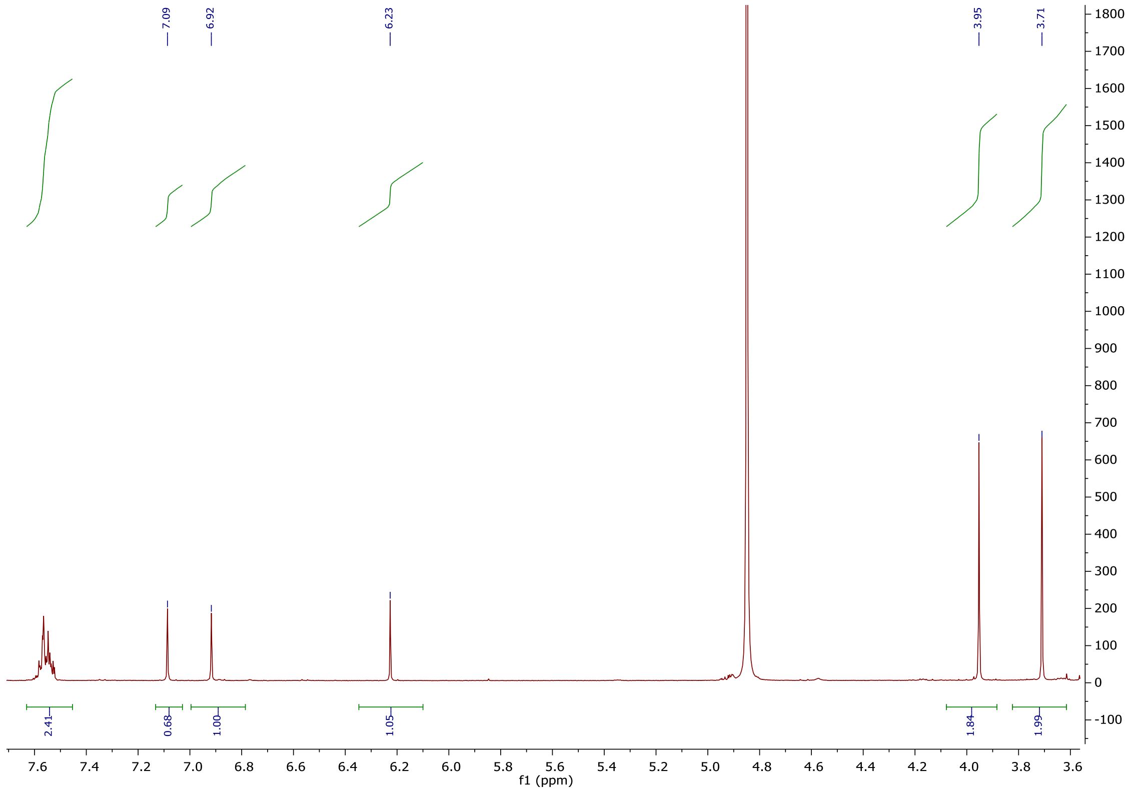

Figure S11_1: ^{1}H NMR spectrum (400 MHz, CD_3OD) of methyl dalbergin (**11**)

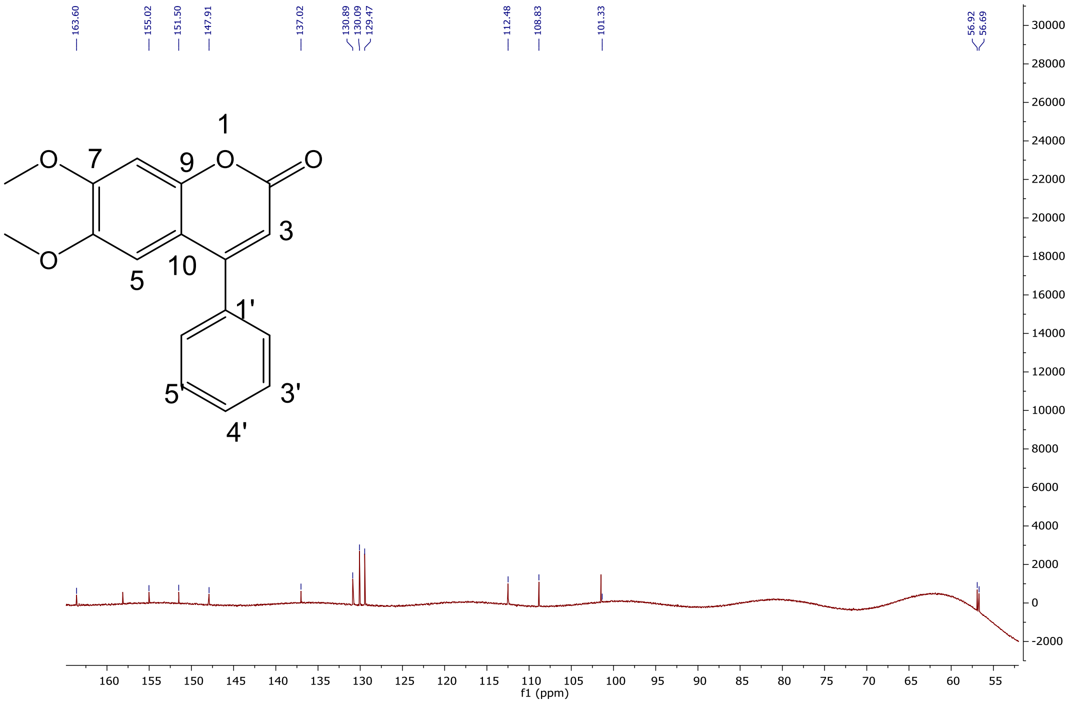

Figure S11_2: ^{13}C NMR spectrum (125 MHz, CD_3OD) of methyl dalbergin (**11**)

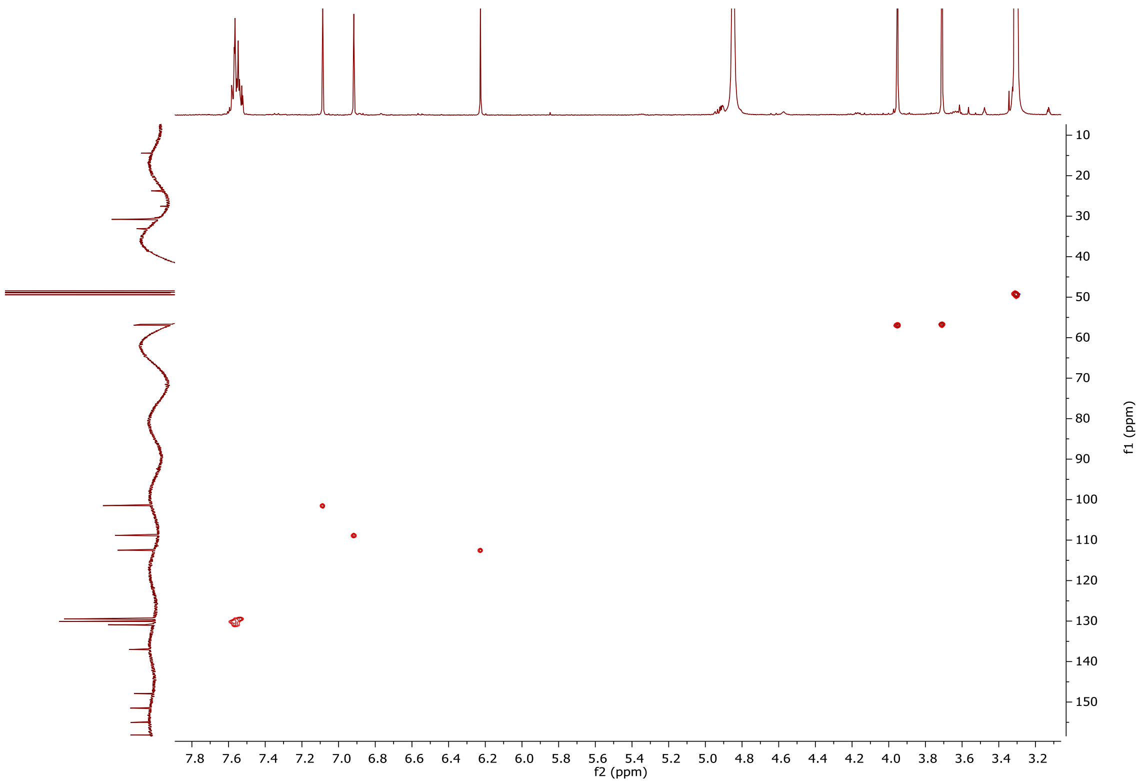

Figure S11_3: HSQCAD spectrum (125 MHz, CD_3OD) of methyl dalbergin (**11**)

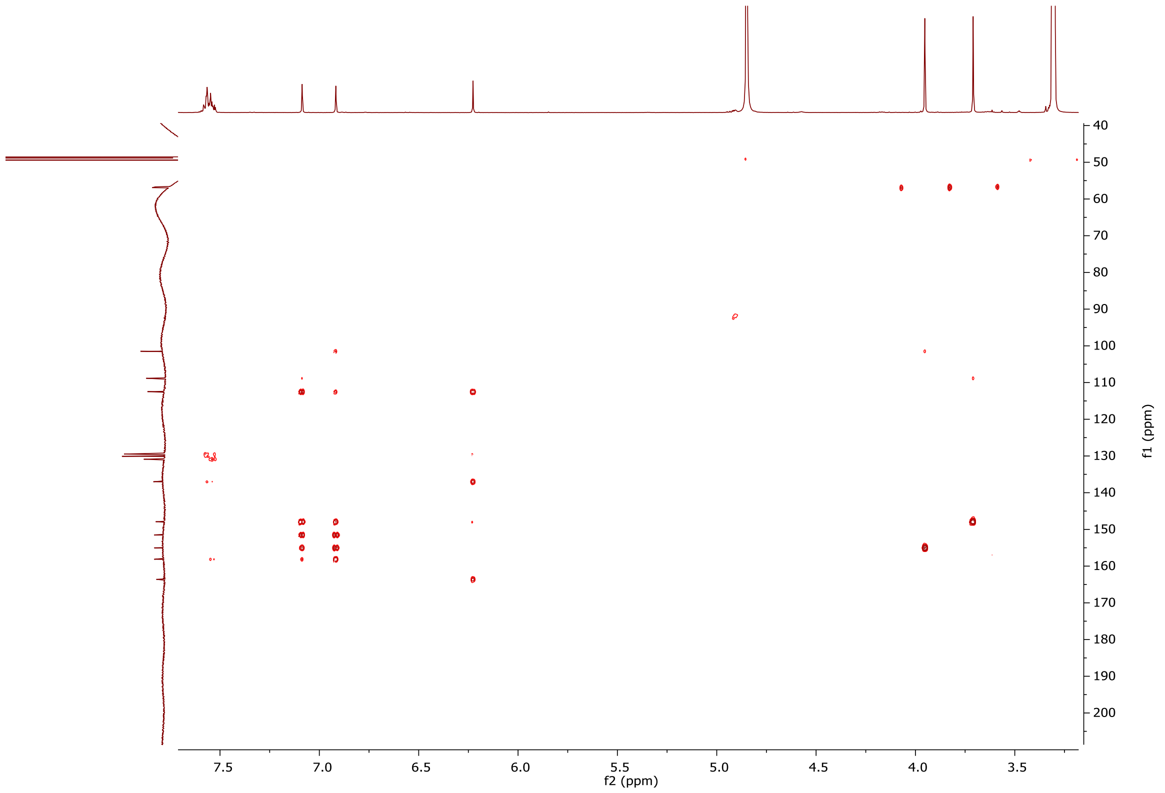

Figure S11_4: HMBC spectrum (125 MHz, CD_3OD) of methyl dalbergin (**11**)

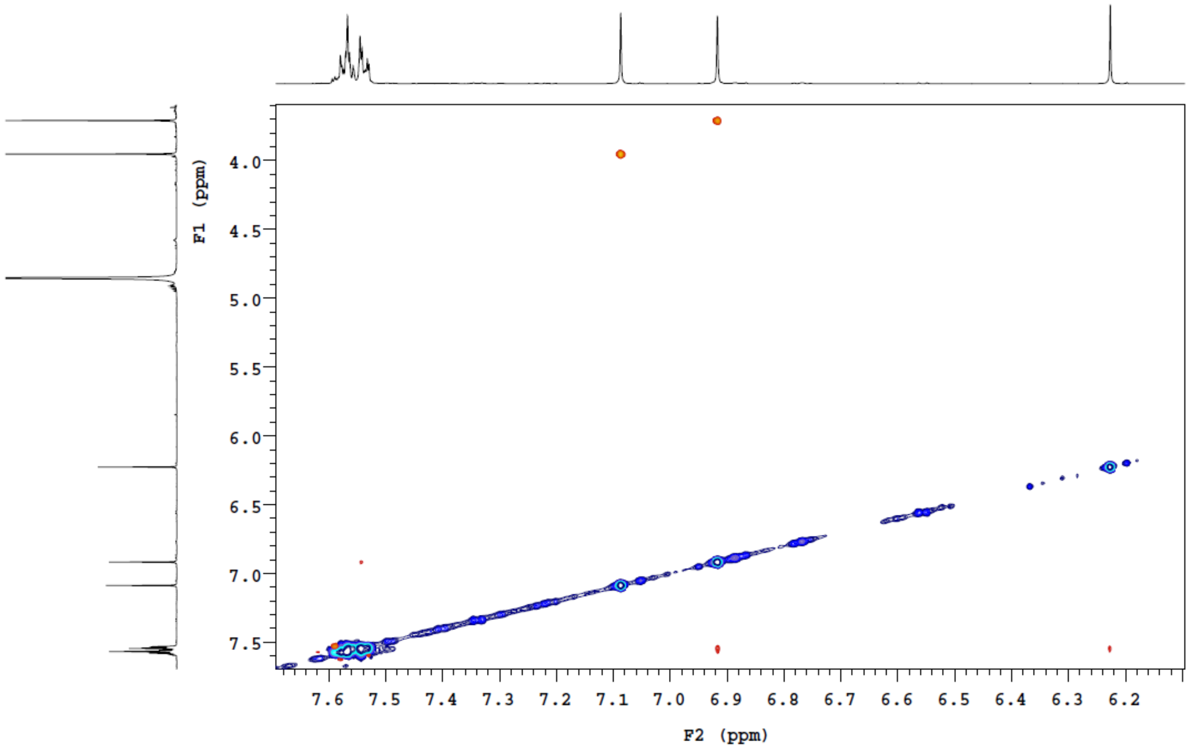

Figure S11_5: ROESY spectrum (125 MHz, CD_3OD) of methyl dalbergin (**11**)

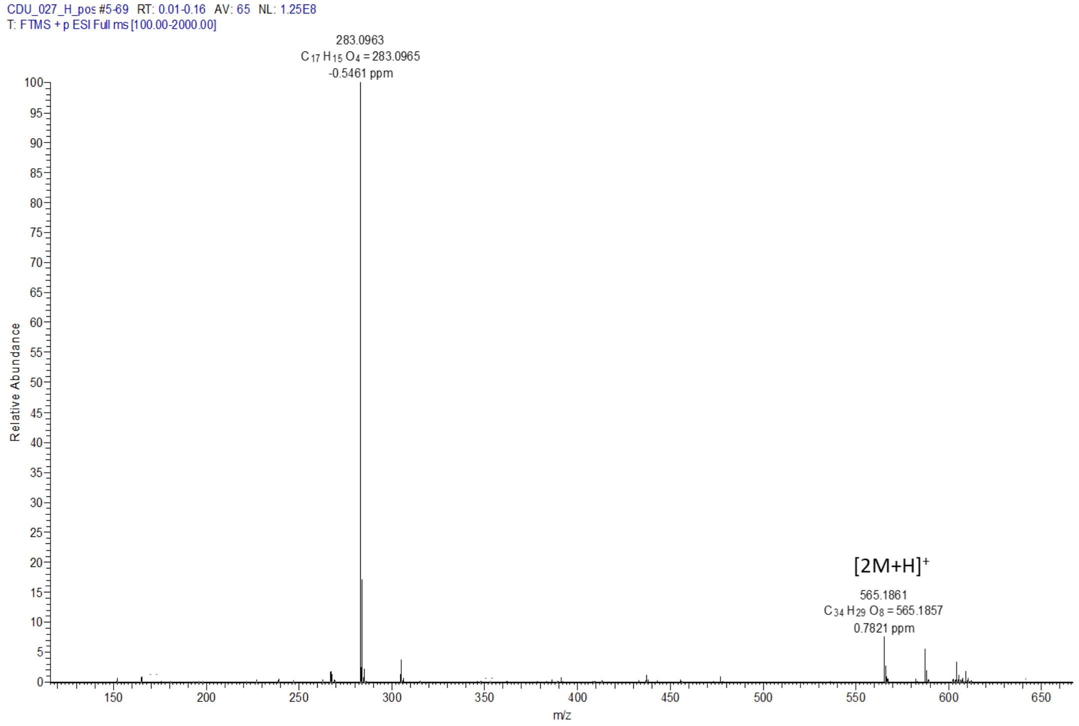

Figure S11_6: Positive ESI-HRMS spectrum of methyl dalbergin (**11**)

Table S11: NMR data (400 MHz, CD_3OD) of methyl dalbergin (**11**)

**No**	**δ_H, *mult* (*J* in Hz)**	**δ_C**	**HMBC**	**ROESY**
2		163.6		
3	6.23, *s*	112.5	C2, C1′, C10	H2′/6′
4		151.5		
5	6.92, *s*	108.8	C10, C7, C9	6-OCH_3, H 2′/6′
6		147.9		
7		155.0		
8	7.10, *s*	101.4	C4, C7, C10	7-OCH_3
9		158.5		
10		112.5		
1′		137.0		
2′/6′	7.54, *m*	129.5		H3, H5
3′/5′	7.57, *m*	130.1		
4′	7.57, *m*	130.9		
6-OCH_3	3.71, *s*	56.7	C6, C5	H5
7-OCH_3	3.96, *s*	56.9	C7, C8	H8

Dalbergin (12)

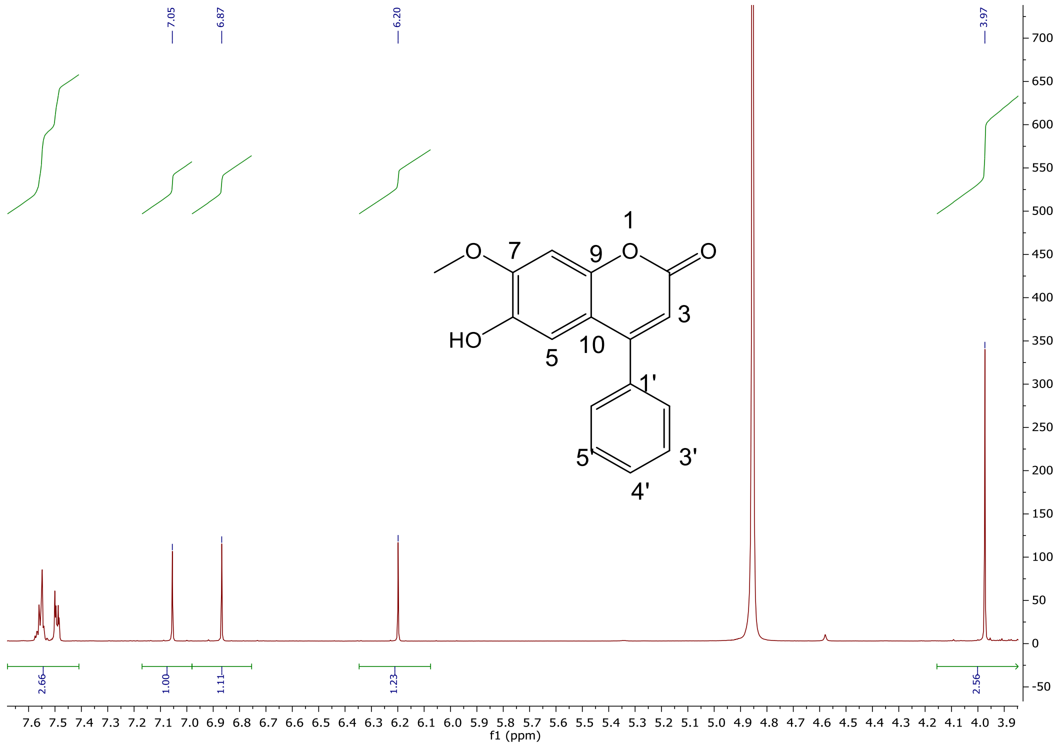

Figure S12_1: ^{1}H NMR spectrum (400 MHz, CD_3OD) of dalbergin (**12**)

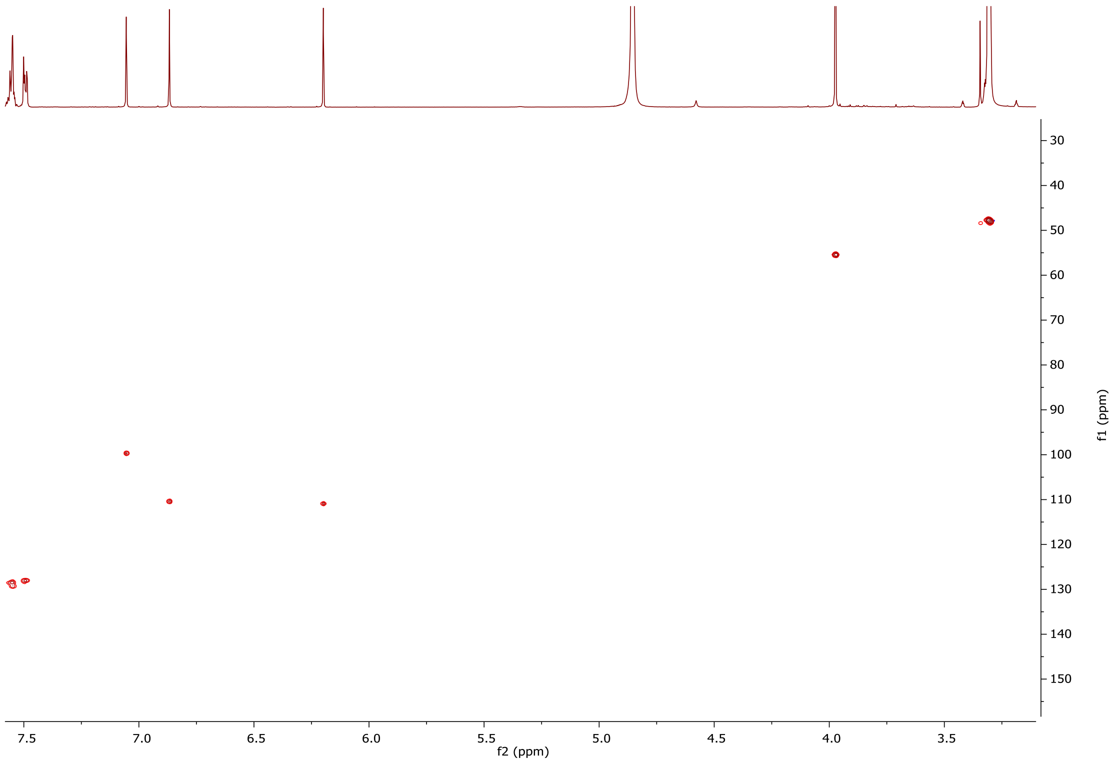

Figure S12_2: HSQC spectrum (400 MHz, CD_3OD) of dalbergin (**12**)

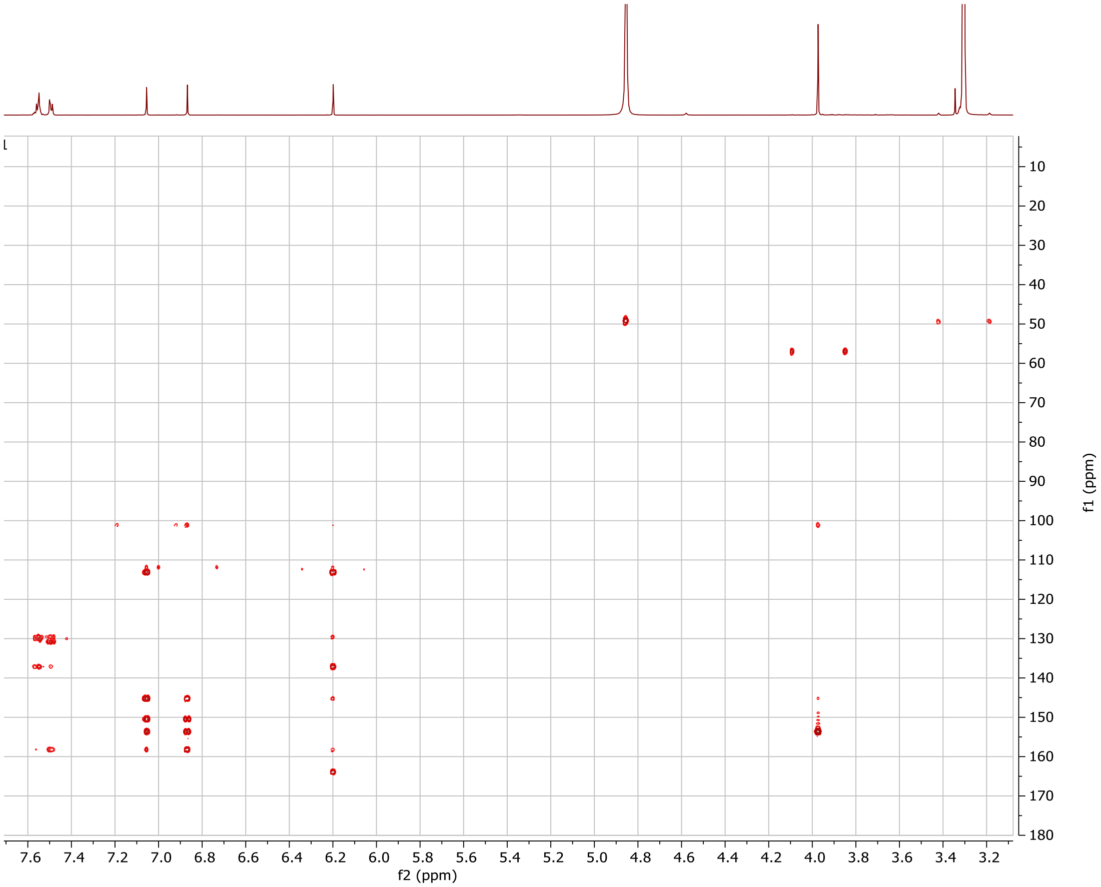

Figure S12_3: HMBC spectrum (400 MHz, CD_3OD) of dalbergin (**12**)

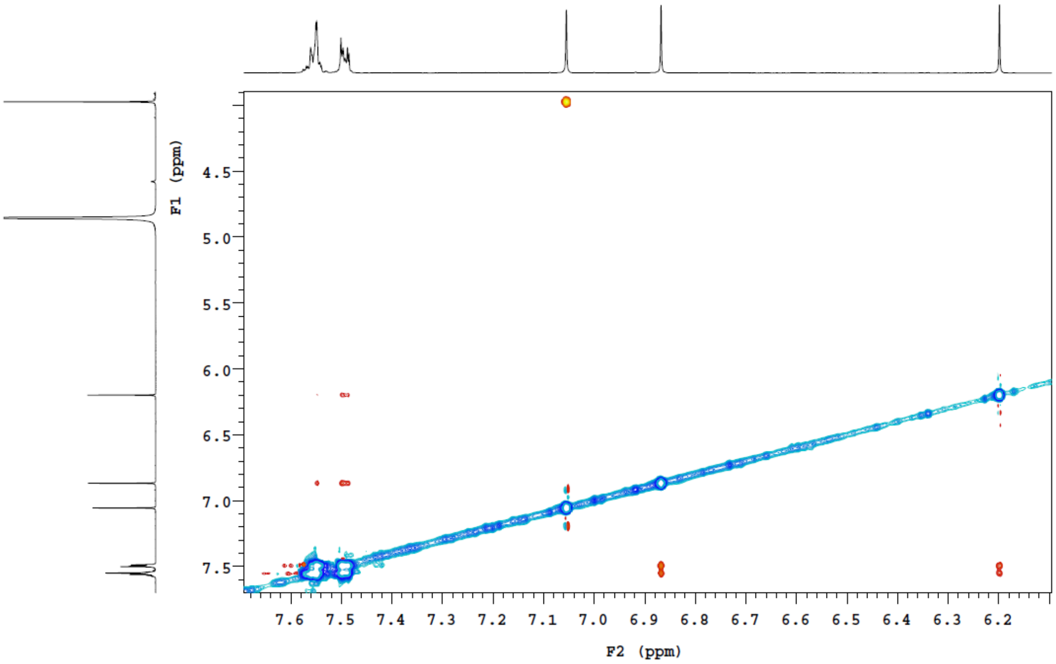

Figure S12_4: ROESY spectrum (400 MHz, CD_3OD) of dalbergin (**12**)

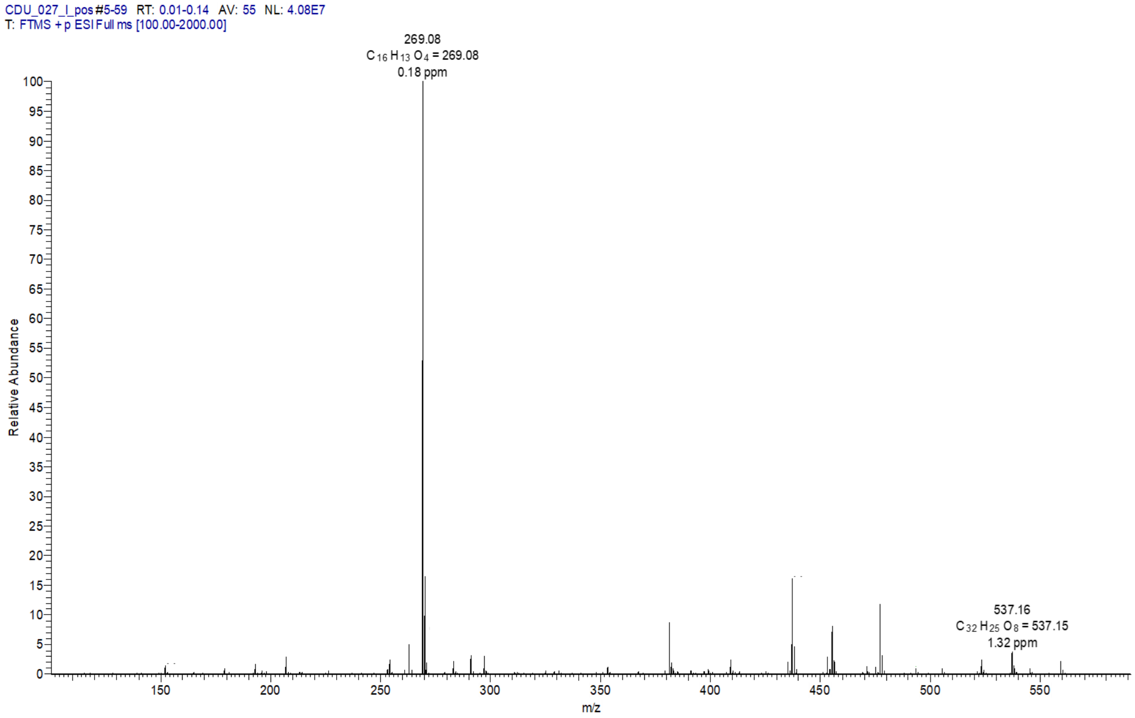

Figure S12_5: Positive ESI-HRMS spectrum of dalbergin (**12**)

Table S12: NMR data (400 MHz, CD_3OD) of dalbergin (**12**)

No	δH, *mult*	δC	HMBC	ROESY	δC (Chan et al. 1997)*
2		163.9			160.85
3	6.20, *s*	112.4	C10, C2, C1′, C4, C6, C2′/6′	H2′/6′, (H 3′/5′)	111.70
4		158.2			155.55
5	6.87, *s*	111.9	C6, C7, C4, C10, C9, C4	H2′/6′	110.75
6		145.2			143.96
7		153.6			152.35
8	7.06, *s*	101.2	C6, C7, C9, C10, C4	7-OCH_3	100.91
9		150.4			148.83
10		113.1			111.32
1′		137.1			135.72
2′/6′	7.50, *m*	129.5	C4′, C2′/6′	H5, H3	128.71
3′/5′	7.55, *m*	130.0	C1′, C2′/6′,		129.24
4′	7.55, *m*	130.8	C3′/5′		129.95
7-OCH_3	3.97, *s*	56.9	C7, C8	H8	56.67

*Chan, S.C., Chang, Y.S., Wang, J.P., Chen, S.C., Kuo, S.C., 1998. Three new flavonoids and antiallergic, anti-inflammatory constituents from the heartwood of *Dalbergia odorifera*. Planta Med. 64 (2), 153–158. https://doi.org/10.1055/s-2006-957394.

Melannein (13)

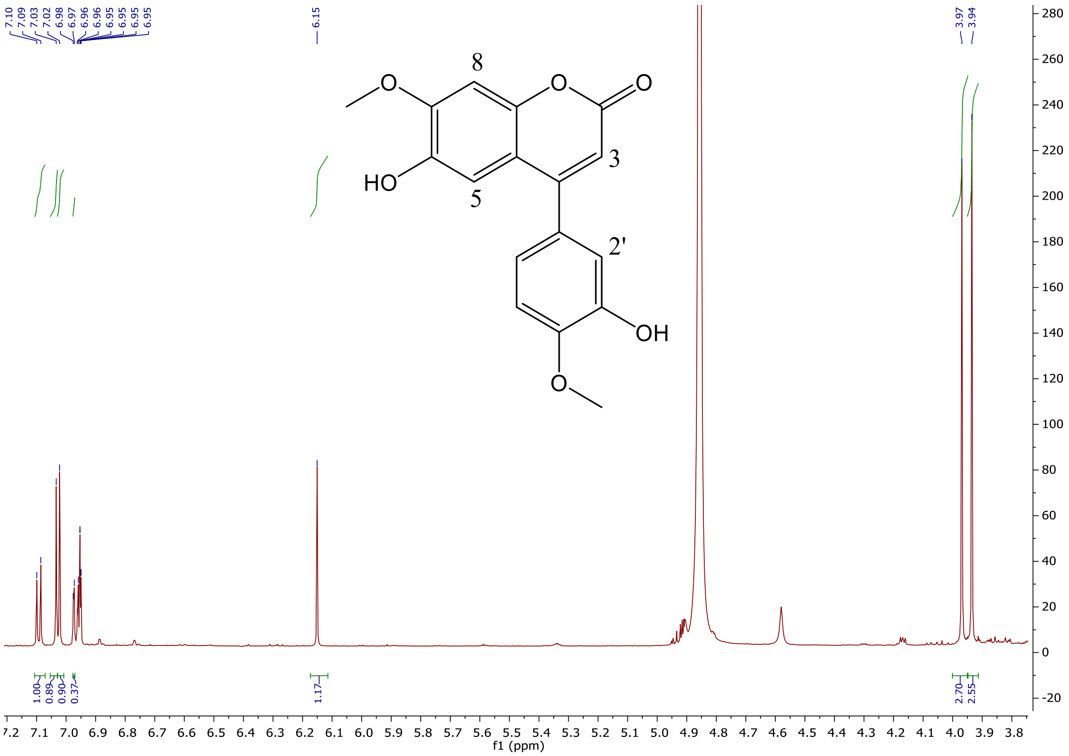

Figure S13_1: ^{1}H NMR spectrum (400 MHz, CD_3OD) of melannein (**13**)

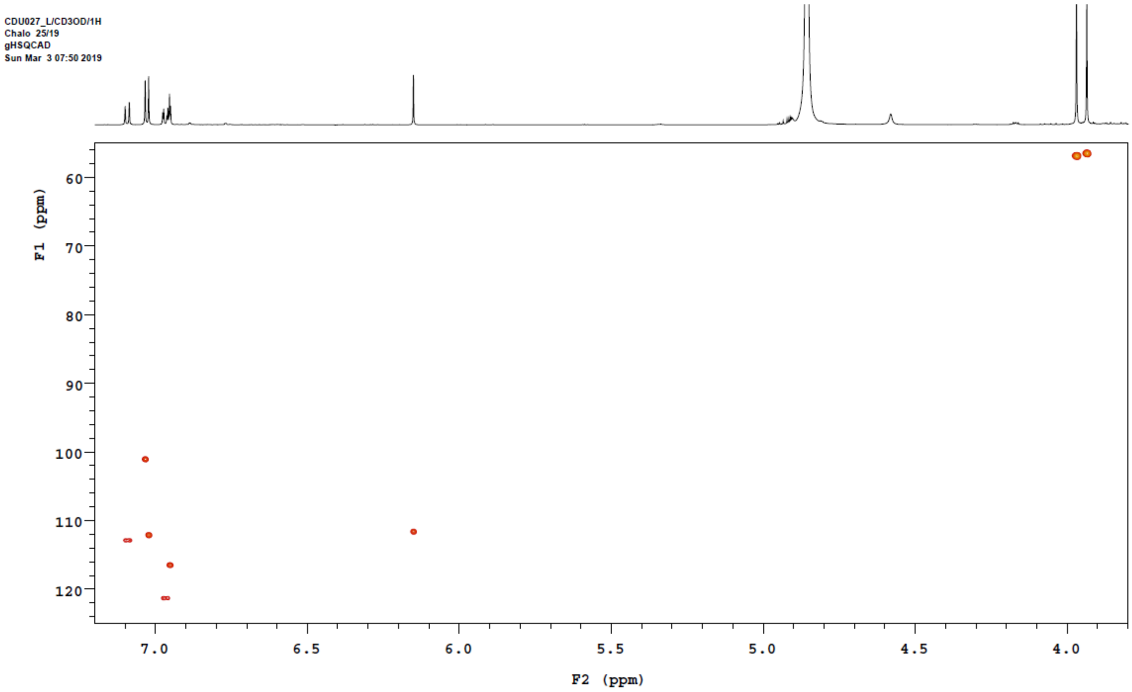

Figure S13_2: HSQCAD spectrum (125 MHz, CD_3OD) of melannein (**13**)

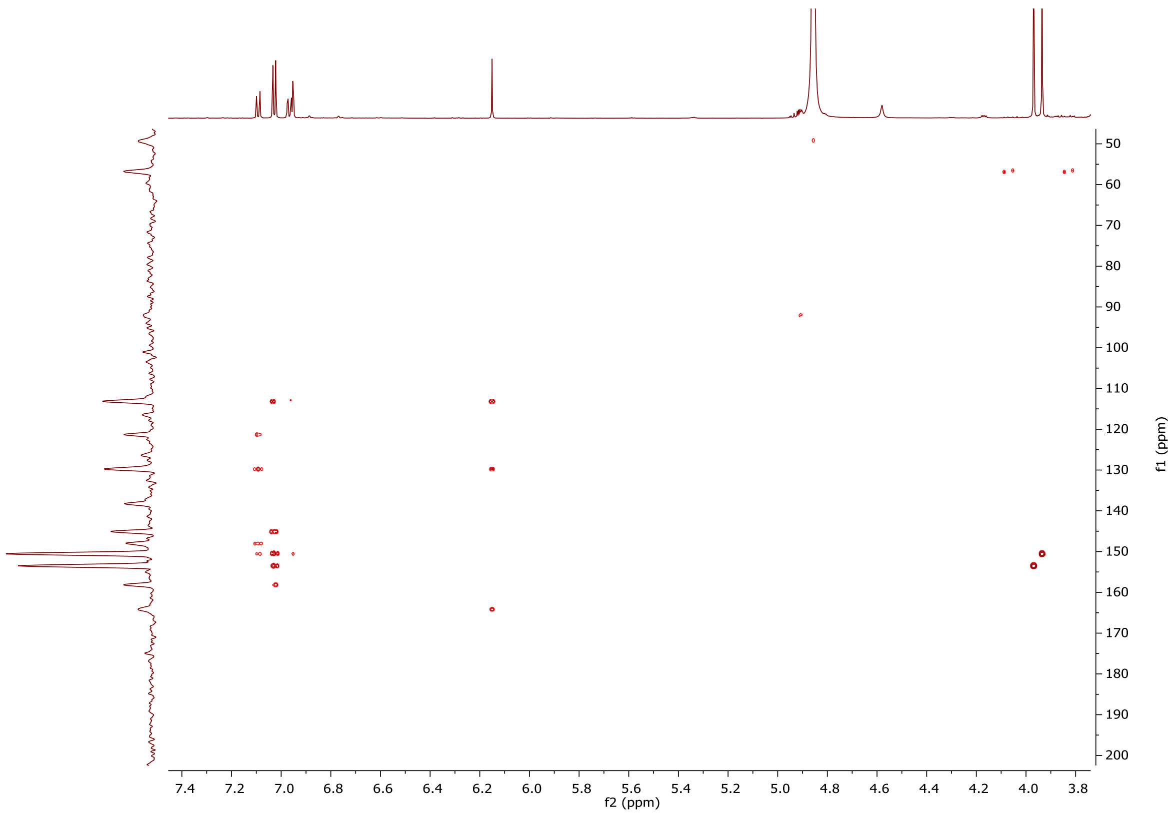

Figure S13_3: HMBC spectrum (125 MHz, CD_3OD) of melannein (**13**)

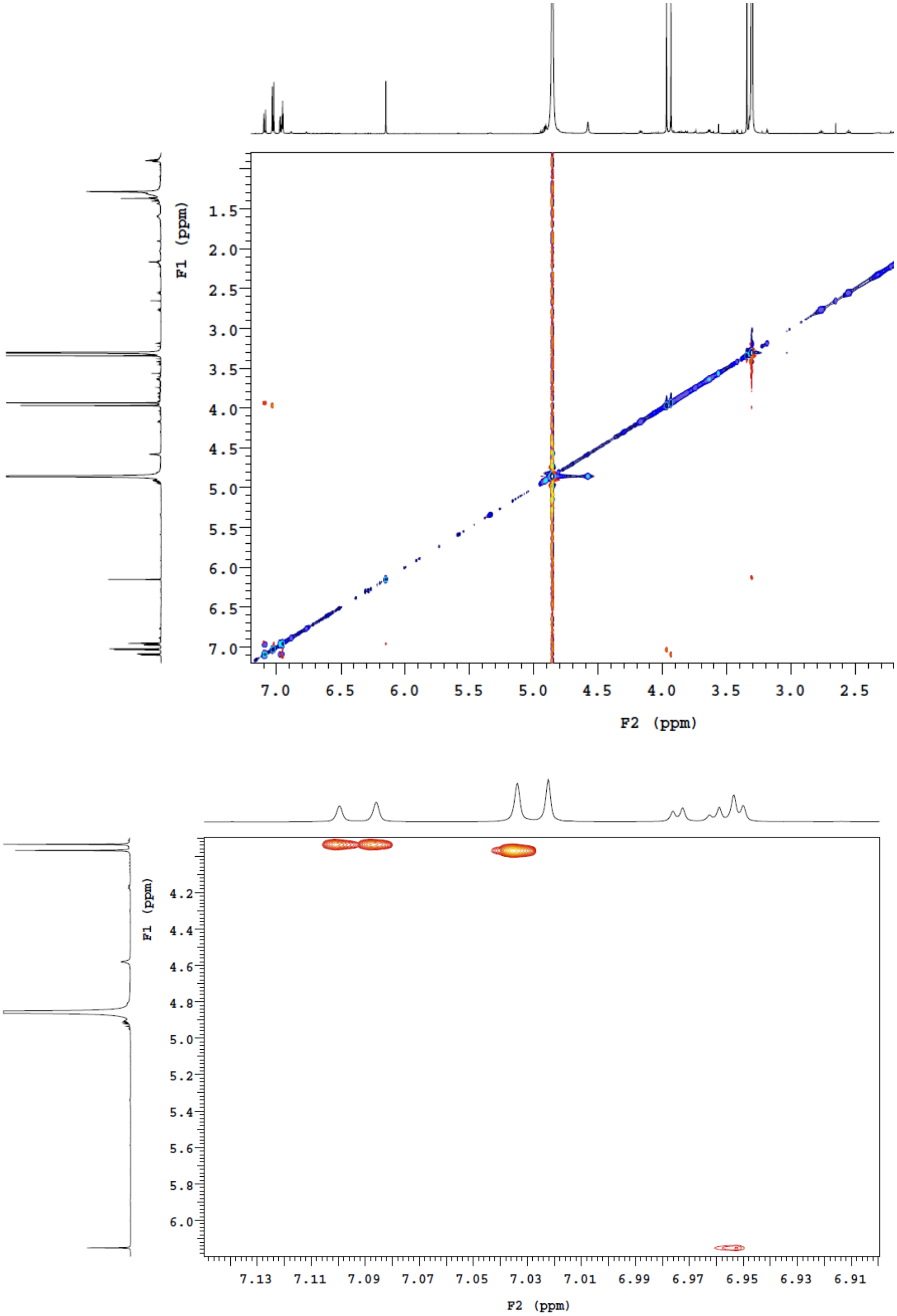

Figure S13_4: ROESY spectrum (125 MHz, CD_3OD) of melannein (**13**)

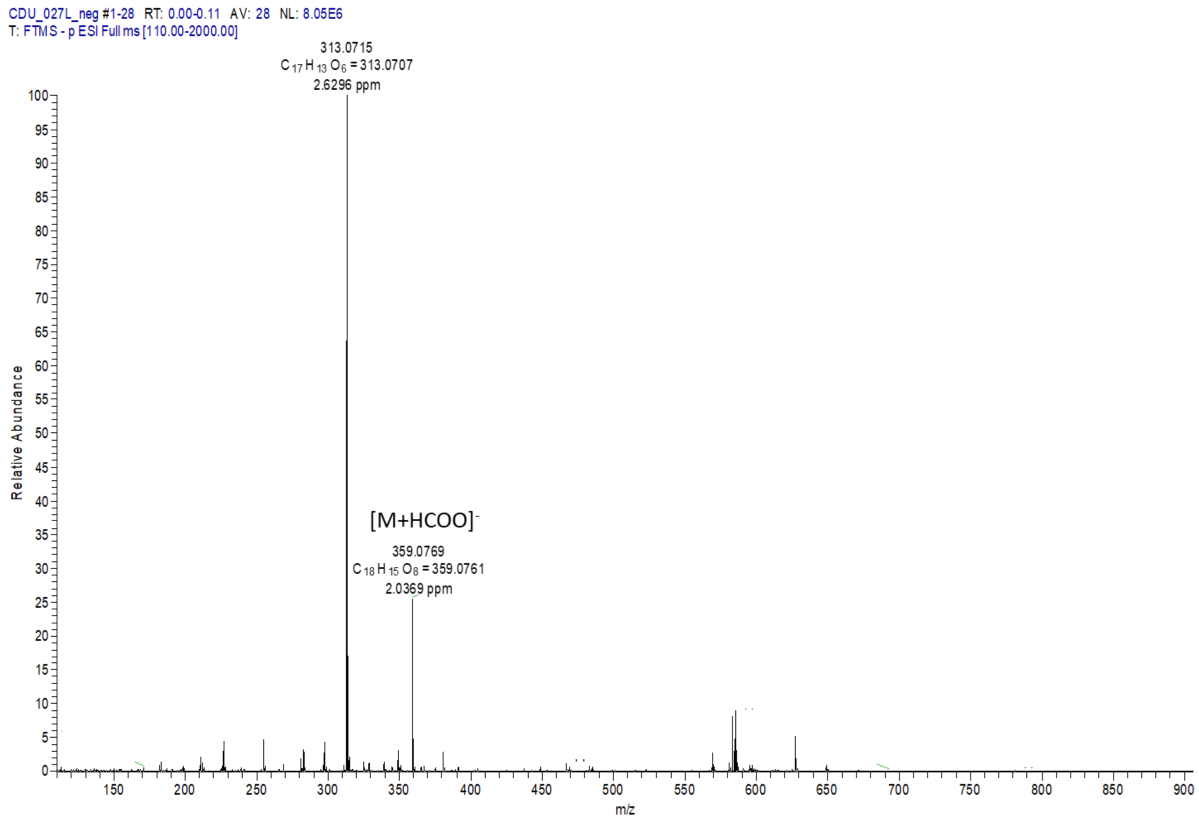

Figure S13_5: Negative ion ESI-HRMS spectrum of melannein (**13**)

Table S13: NMR data (400 MHz, CD_3OD) of melannein (**13**)

No	δ_H, *mult* (*J* in Hz)	δ_C	HMBC	ROESY
2		164.2		
3	6.15, *s*	111.6	C10, C2, C1′	H6′
4		158.2		
5	7.02, *s*	112.1	C4, C6, C7	
6		145.1		
7		153.5		
8	7.03, *s*	101.2	C10, C6, C9, C7	7-OCH_3
9		150.5		
10		113.2		
1′		129.8		
2′	6.97, *d* (2.2)	121.4	C4′	
3′		148.0		
4′		150.6		
5′	7.09, *d* (8.6)	113.0	C1′, C2′, C3′, C4′	H6′, 4′-OCH_3
6′	6.95, *dd* (8.6, 2.2)	116.5	C4′	H3, H5′
7-OCH_3	3.97, *s*	56.9	C7	H8
4′-OCH_3	3.94, *s*	56.5	C4′	H5′

3′,4′-Dihydroxy-*trans*-cinnamic acid octacosylester (14)

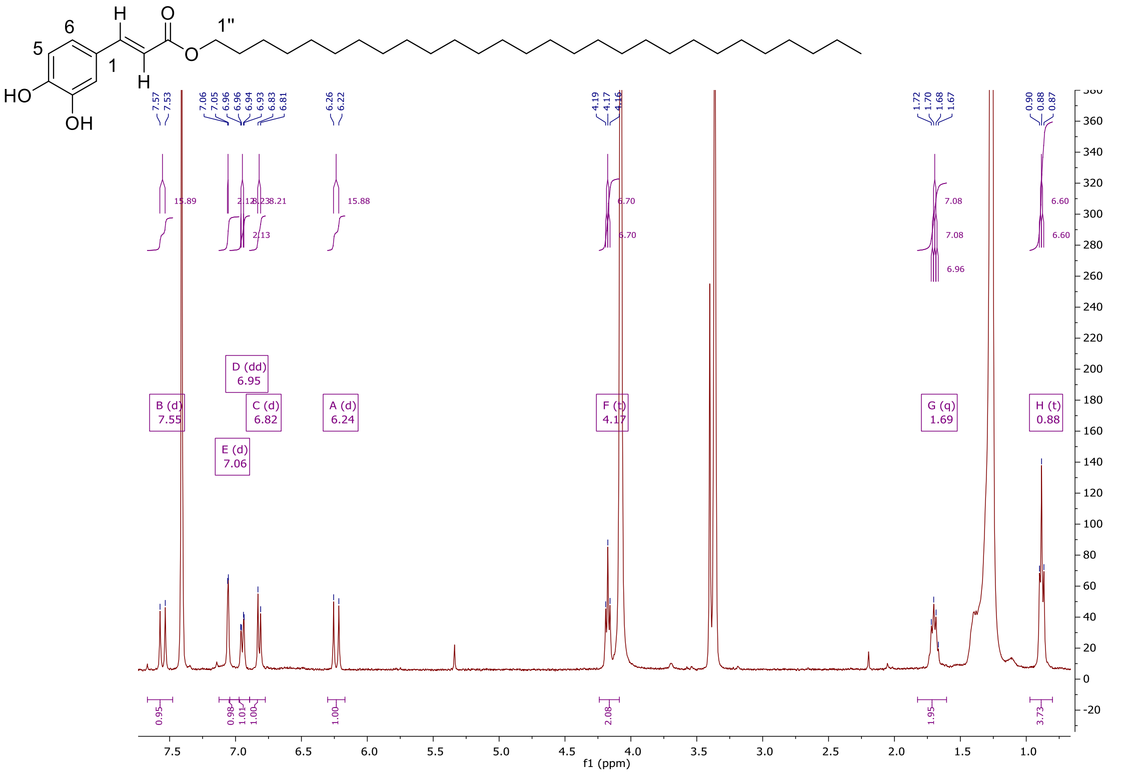

Figure S14_1: ^{1}H NMR spectrum (400 MHz, $CDCl_3$ and few drops CD_3OD) of compound **14**

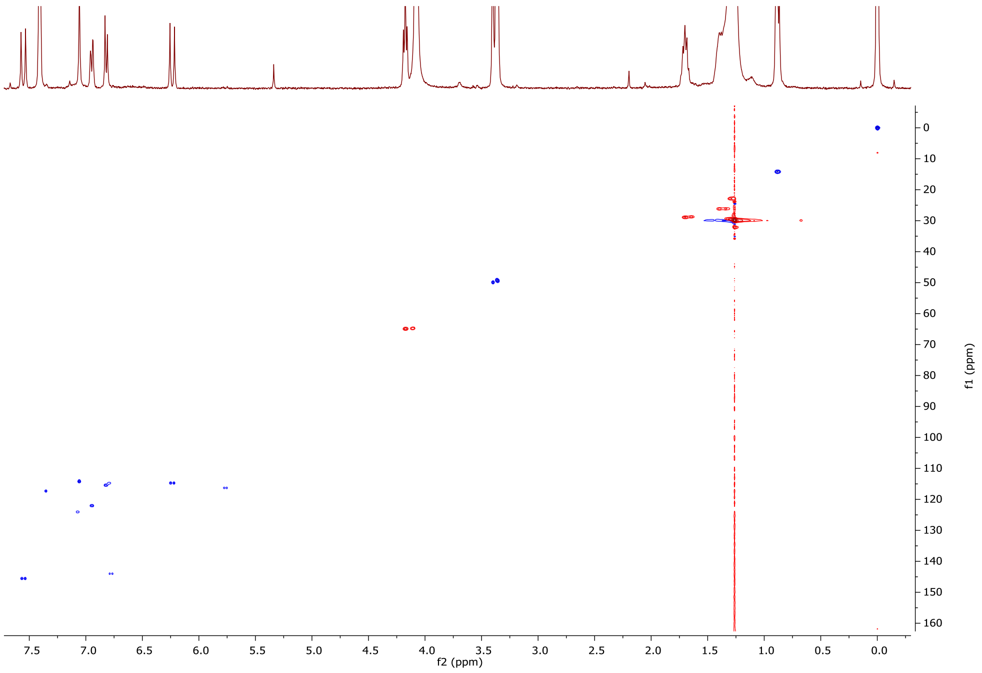

Figure S14_2: HSQCAD spectrum (400 MHz, $CDCl_3$ and few drops CD_3OD) of compound **14**

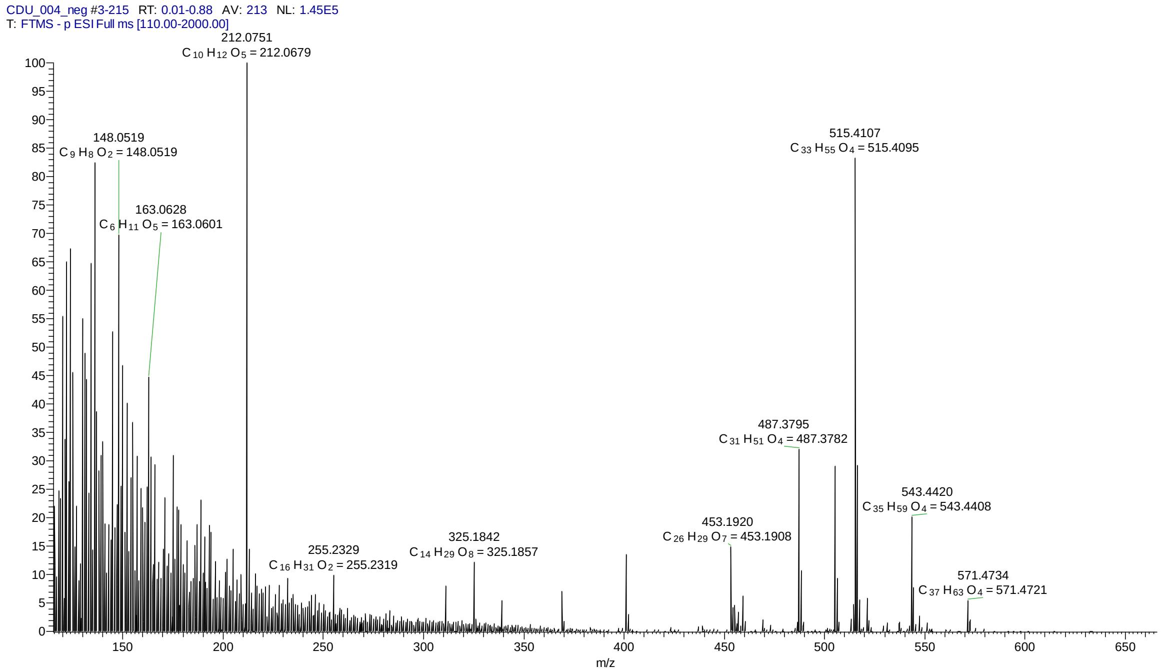

Figure S14_3: Negative ion ESI-HRMS spectrum of compound **14**

Table S14: NMR data of compound **14**

No	δ$_H$, *mult* (*J* in Hz)	δ$_C$ (HSQC)	
1			
2	7.06, *d* (2.1)	114.2	CH
3			
4			
5	6.82, *d* (8.2)	115.4	CH
6	6.95, *dd* (8.2, 2.1)	122.0	CH
1′	7.55, *d* (15.9)	145.5	CH
2′	6.24, *d* (15.9)	114.7	CH
3′			
1″	4.17, *t* (6.7)	65.0	CH_2
2″	1.69, *m*	28.9	CH_2
3″	1.39	26.1	CH_2
4″	1.30	29.3	CH_2
5″-20″	1.26	29.9	CH_2
21″	1.26	29.9	CH_2
22″	1.26	32.1	CH_2
23″	1.26	23.9	CH_2
24″	0.88, *t* (6.6)	14.2	CH_3

Table S15: Cytotoxic activities of crude extract of *D. melanoxylon* against human cancer cell lines determined by MTT (cell viability) and CV (cell growth) assays. Values show survival [%].

	**PC-3**				**HT-29**			
	**MTT**		**CV**		**MTT**		**CV**	
**Crude extract**	50 µg/ml	0.05 µg/ml	50 µg/ml	0.05 µg/ml	50 µg/ml	0.05 µg/ml	50 µg/ml	0.05 µg/ml
	1 ± 37	96 ± 4	1 ± 18	104 ± 4	0 ± 25	98 ± 4	0 ± 5	98 ± 2
Positive control*	0 ± 29		-1 ± 7		0 ± 40		0 ± 7	
Negative control	100 ± 4		100 ± 7		100 ± 5		100 ± 3	

* Digitonin, 125 µg/ml

Table S16: Antifungal activity of compounds from *D. melanoxylon* against human pathogens determined by agar diffusion assay (inhibition zone [mm]) and microdilution (MIC [µg/ml])

**compounds** (1mg/ml)	*S. salmonicolor* 549 H4 [mm]	*C. albicans* H8 [mm]	*P.* notatum JP36 P1 [mm]	*A. fumigatus* ATCC204305 [µg/ml]
**1**	0	11P	12P	n.t.
**2**	17	0	11P	n.t.
**7**	0/A	12	14	32
**9**	0	0/A	15(p)	>128
**10**	17	12p	15	>128
Ampho B[a]	19p	21	18p	n.t.
DMSO[b]	13P	0	12P	>128

[a]Amphotericin B, positive control tested at concentration of 10 µg/ml
[b]negative control
